# Supplementary material for: Integrative computational, synthetic, experimental evaluation of targeted inhibitors against matrix metalloproteinase-9: Toward precision modulation of proteolytic activity
Source: PLoS One. 2026 Feb 17;21(2):e0337544. doi: 10.1371/journal.pone.0337544 (PMC12912705; doi:10.1371/journal.pone.0337544)
Supplement: S1 File — 1H-NMR, 13C-NMR, and HRMS (ESI) spectra of synthesized compounds. (DOCX) [file pone.0337544.s001.docx]

**Supplementary File**

**Supplementary data charts (S#1)**

**Compounds (5)**

**
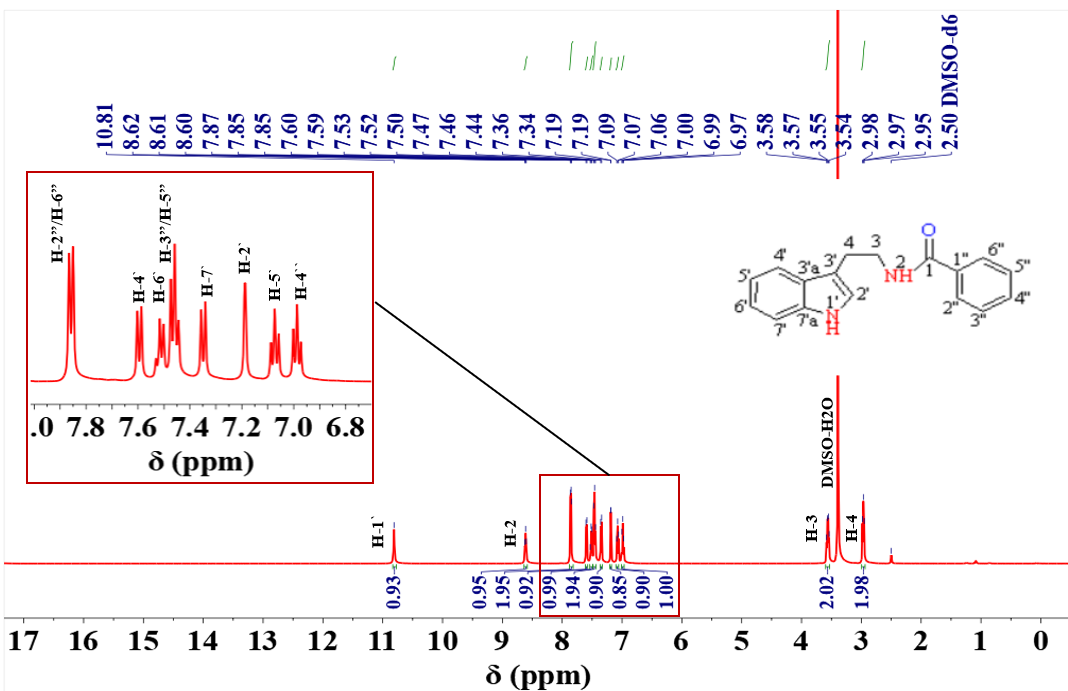
**

**The ^1^H-NMR spectrum of compound 5.**

**
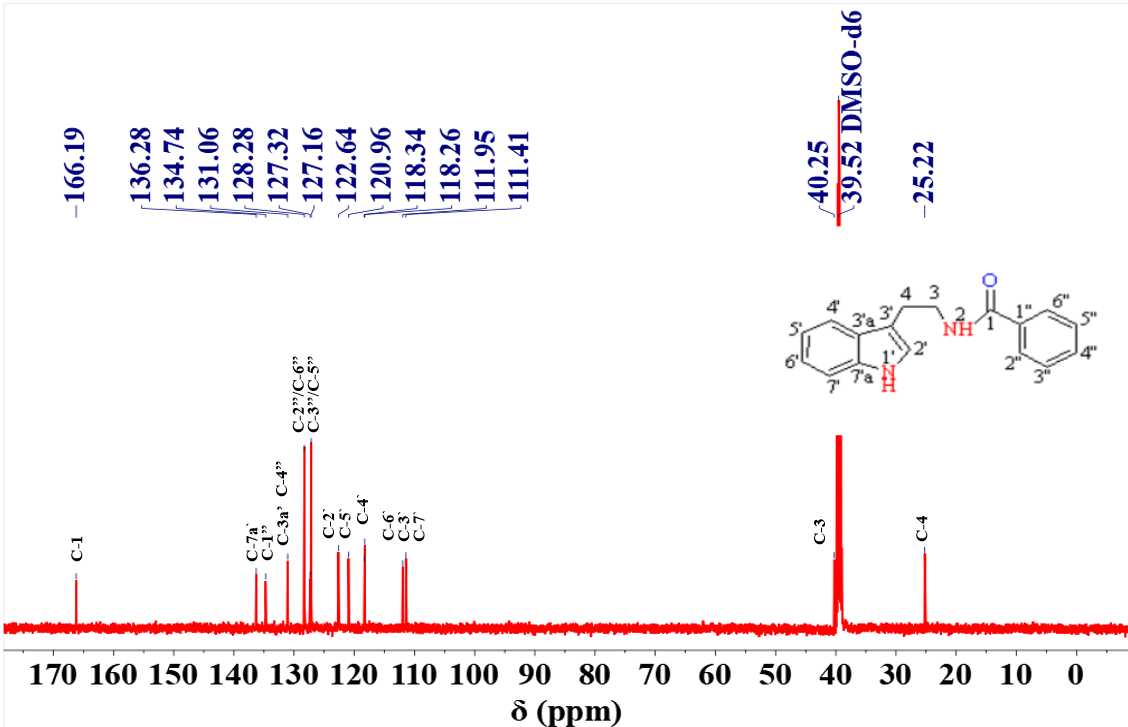
**

**The** **^13^C-NMR spectrum of compound 5.**

**
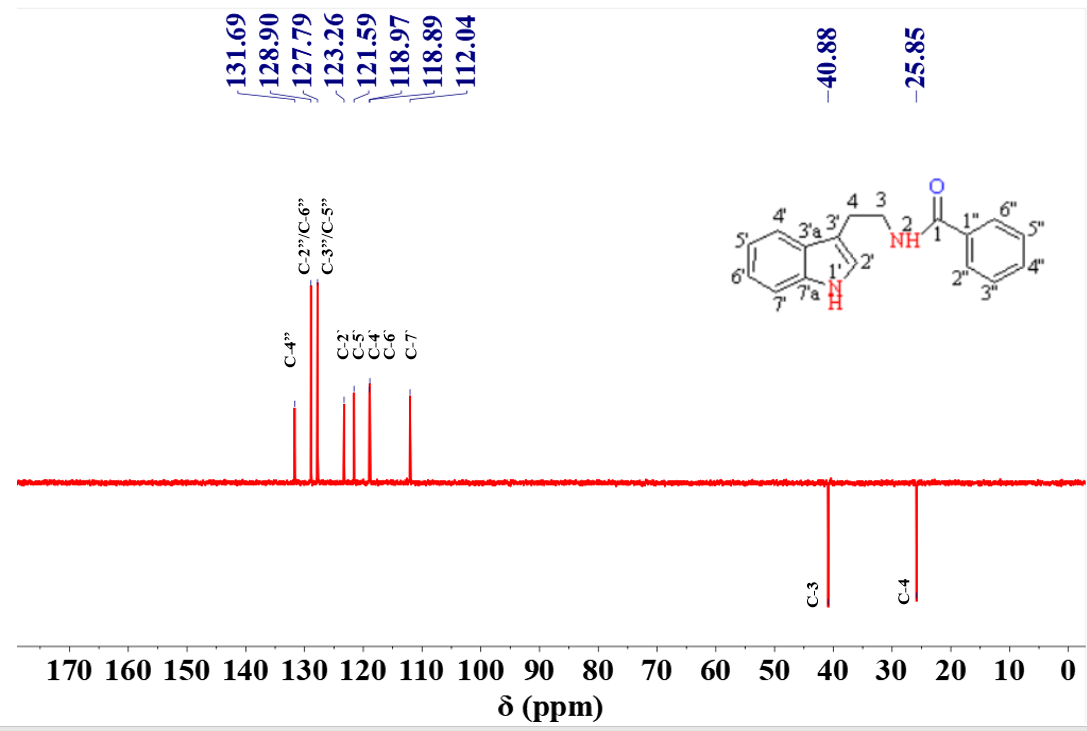
**

**The** **DEPT** **^13^C-NMR spectrum of compound 5.**


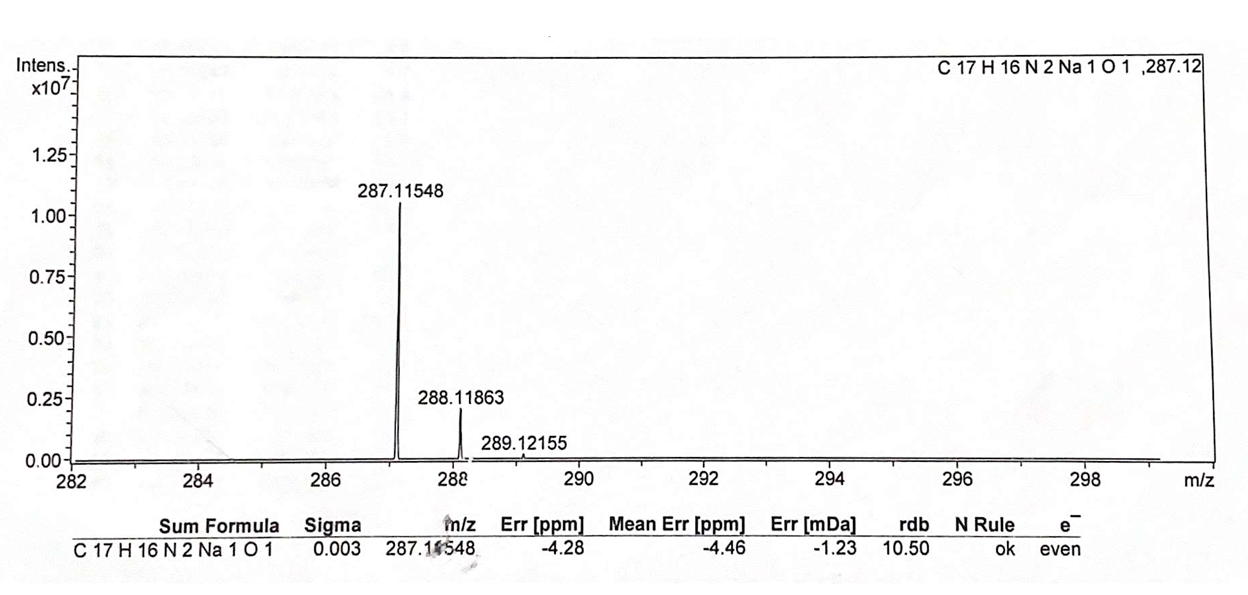


**The** **HRMS (ESI) spectrum of compound 5.**

**Supplementary data charts (S# 2)**

**Compounds (6)**


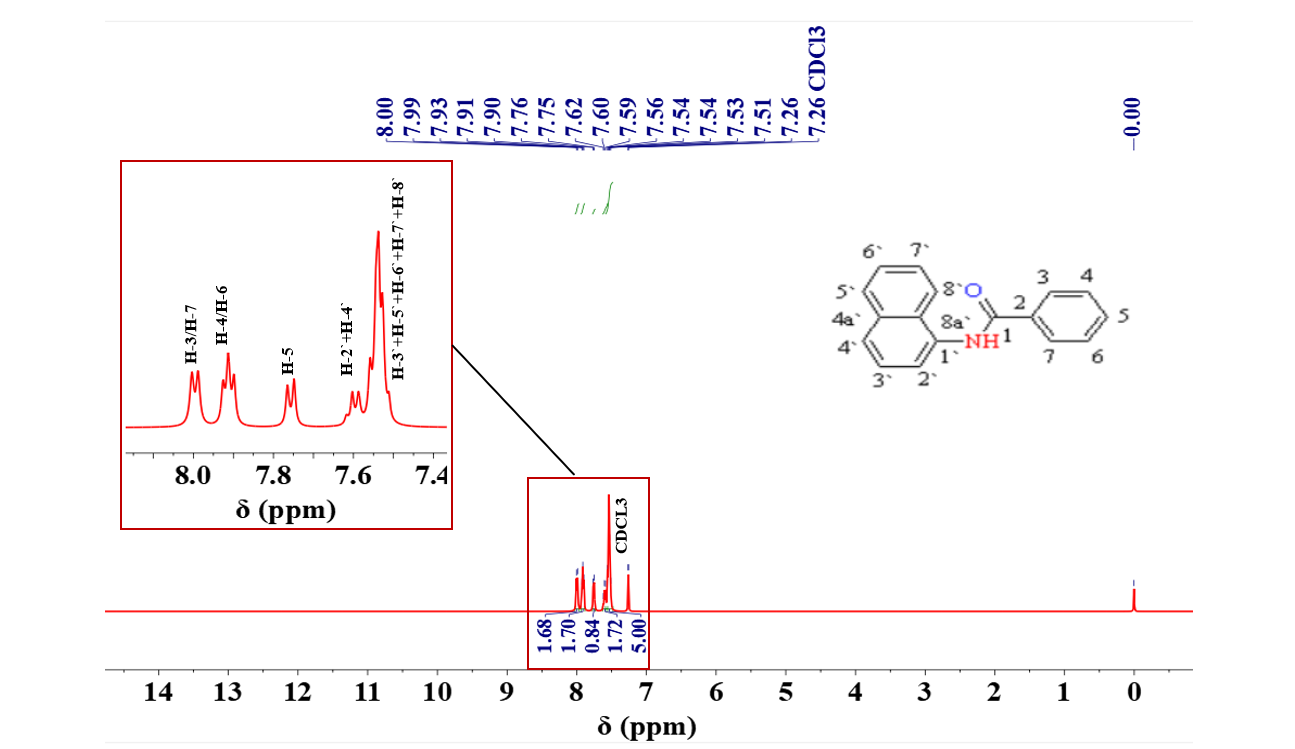


**The ^1^H-NMR spectrum of compound 6.**


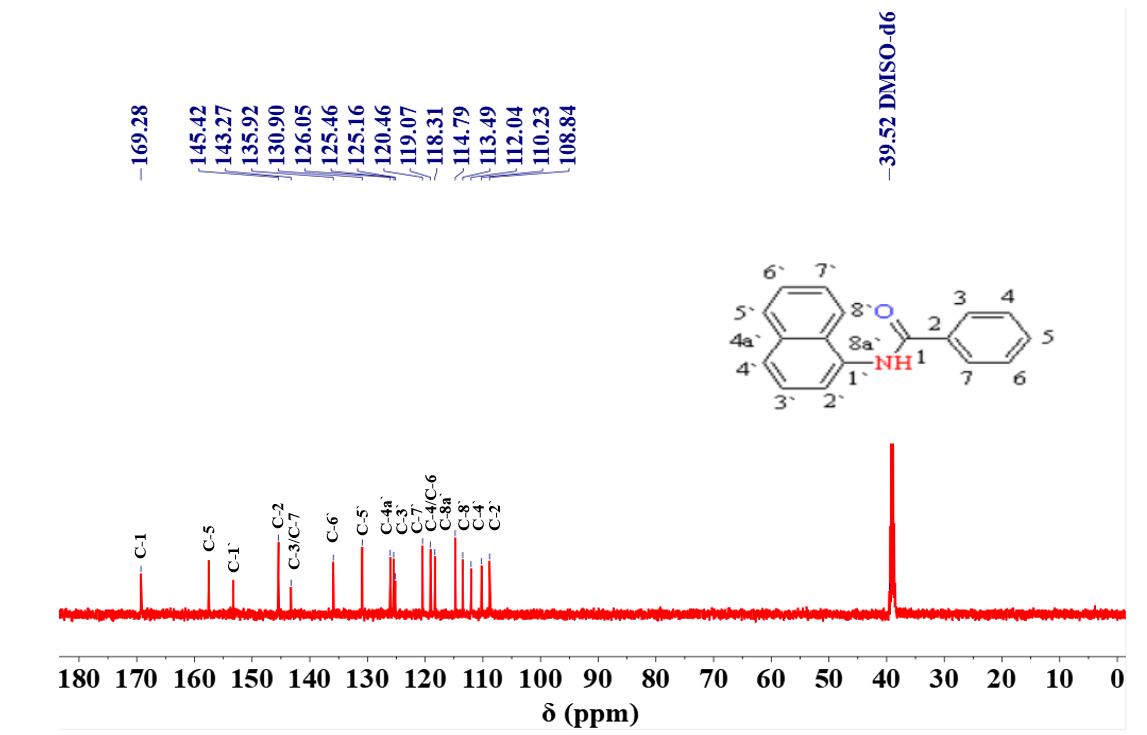


**The** **^13^C-NMR spectrum of compound 6.**

**Supplementary data charts (S# 3)**

**Compounds (7)**


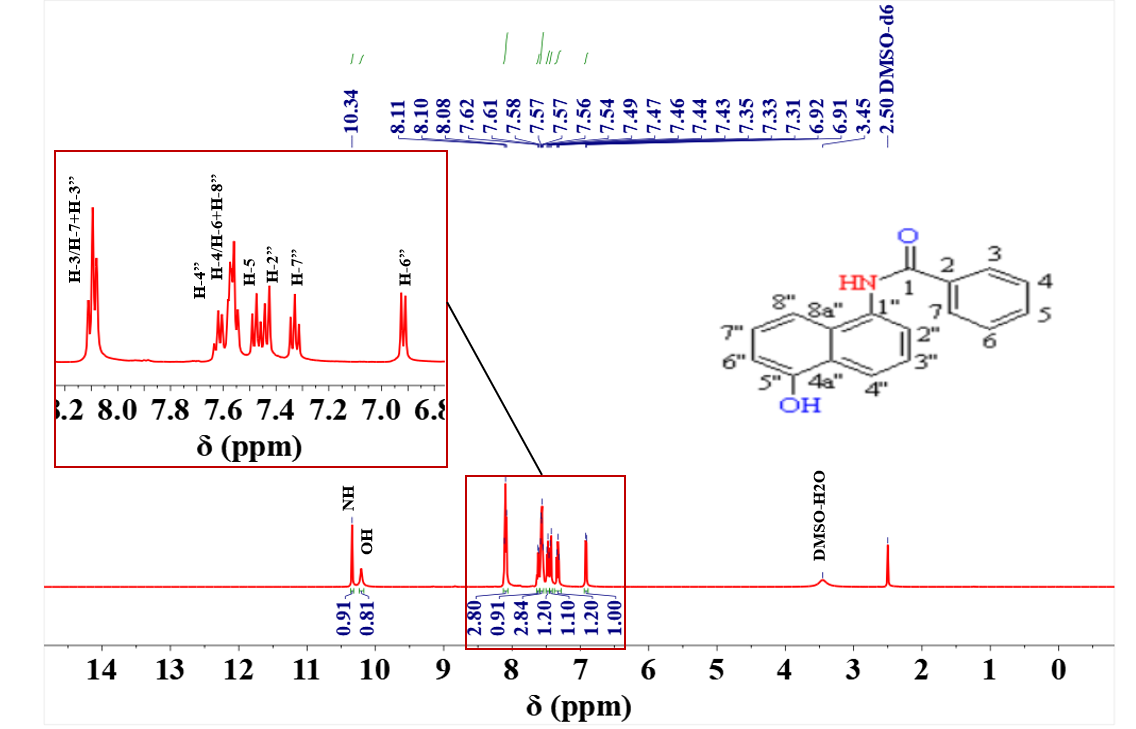


**The ^1^H-NMR spectrum of compound 7.**


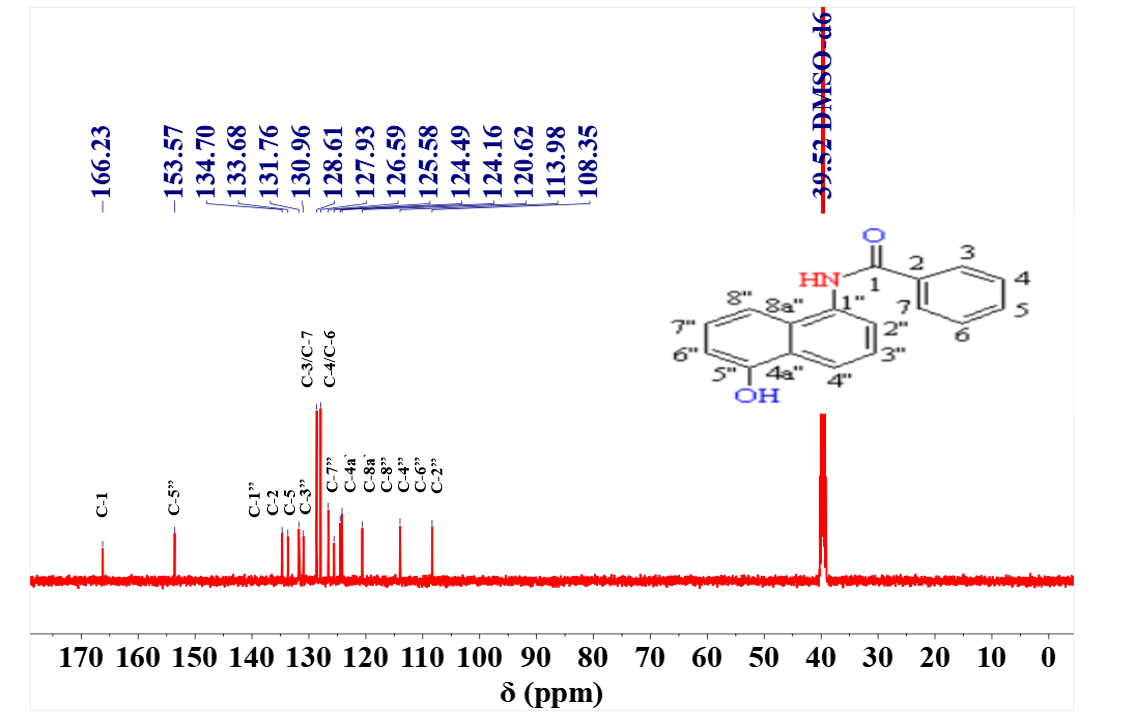


**The** **^13^C-NMR spectrum of compound 7.**

**
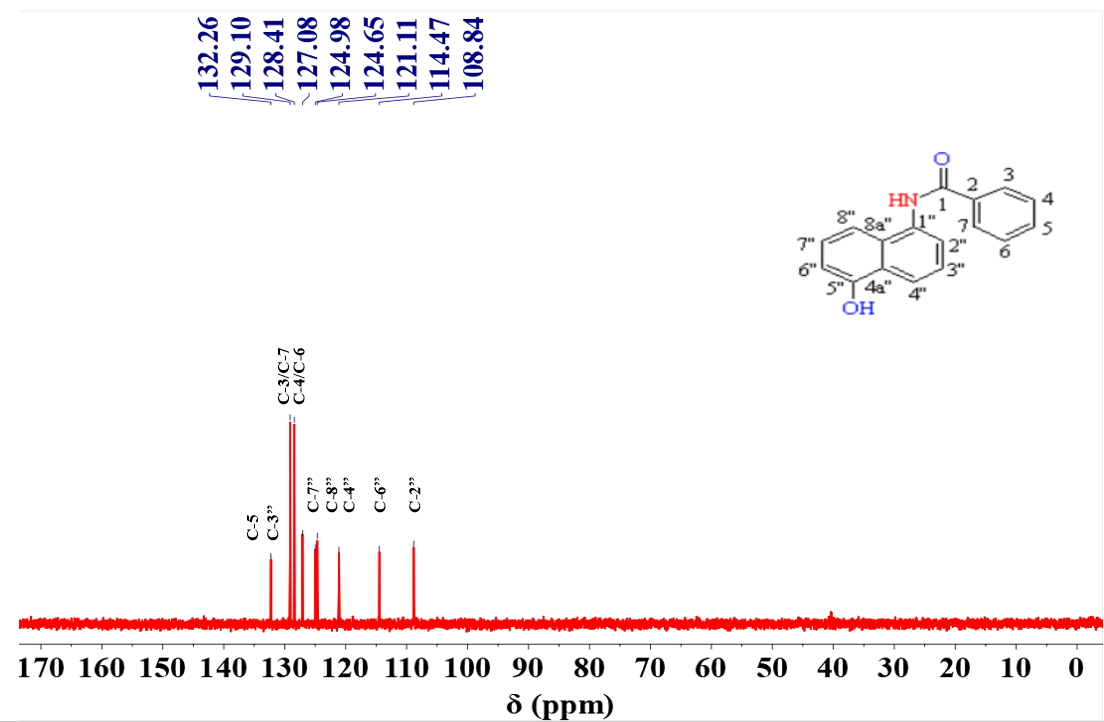
**

**The** **DEPT** **^13^C-NMR spectrum of compound 7.**


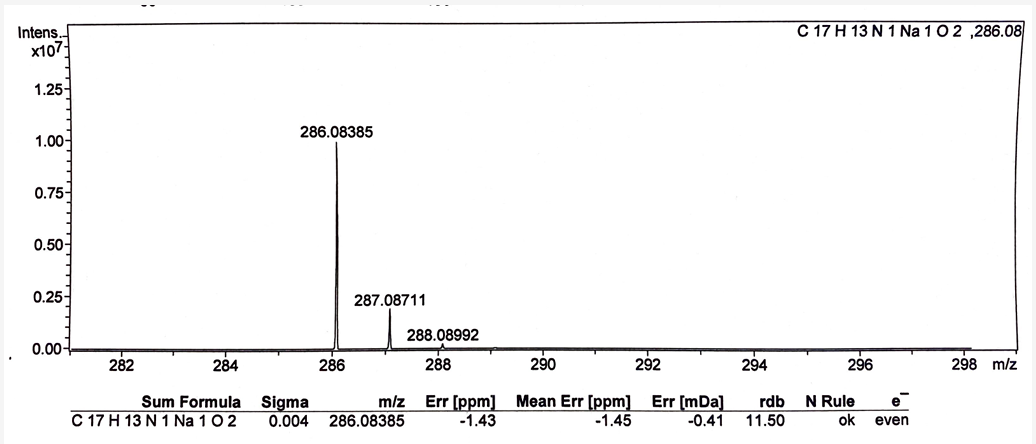


**The** **HRMS (ESI) spectrum of compound 7.**

**Supplementary data charts (S# 4)**

**Compound** (**8**)


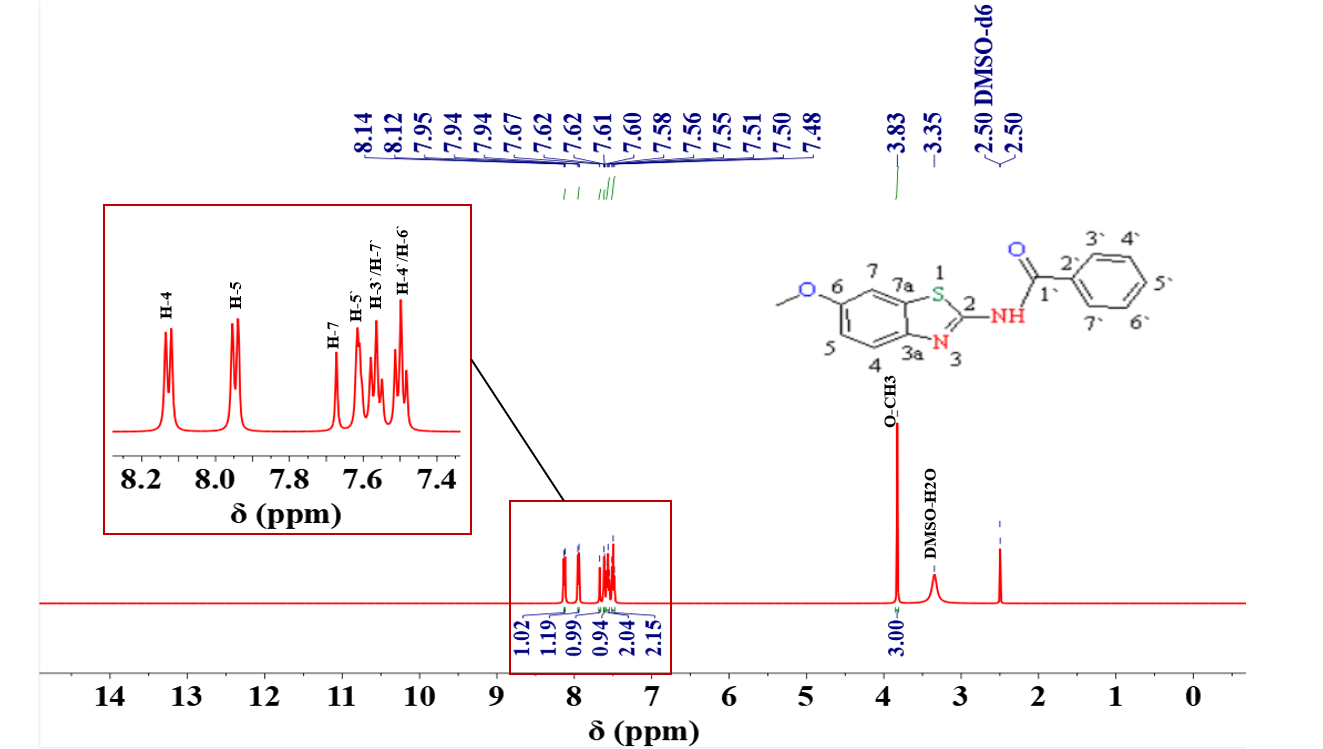


**The ^1^H-NMR spectrum of compound 8.**


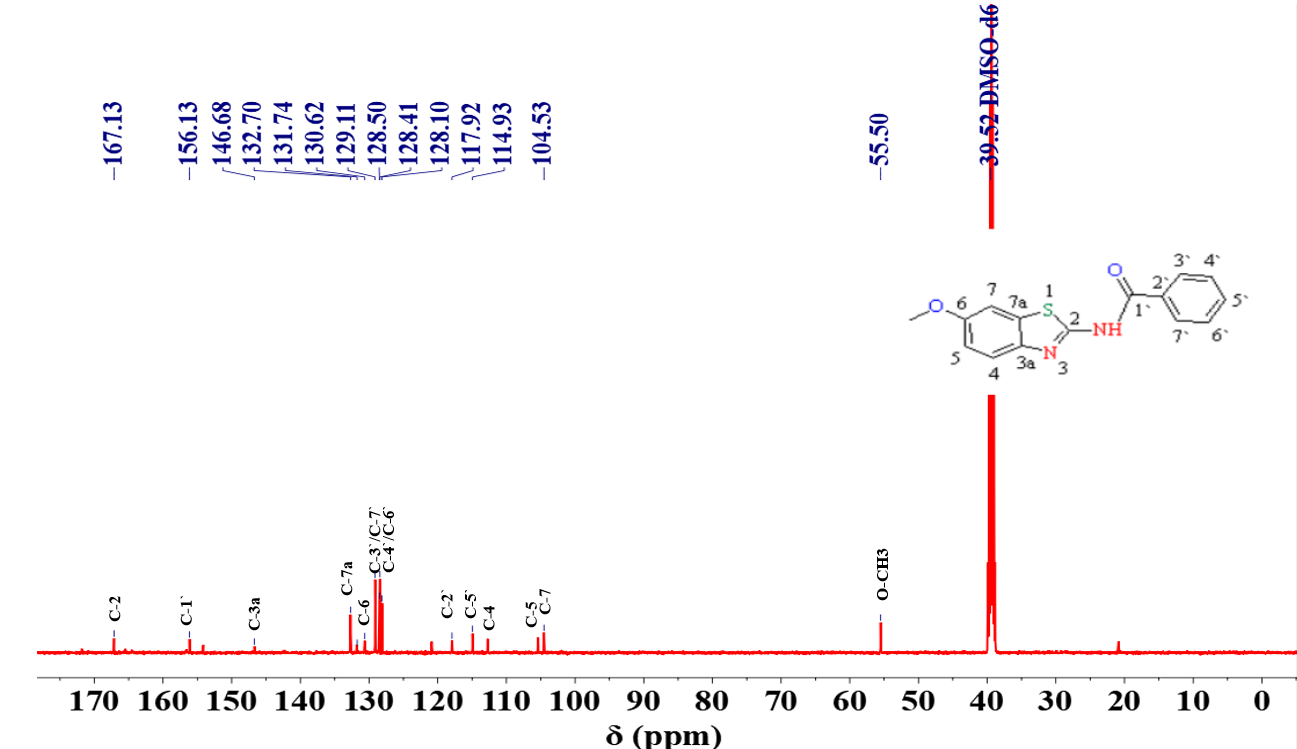


**The** **^13^C-NMR spectrum of compound 8.**

**
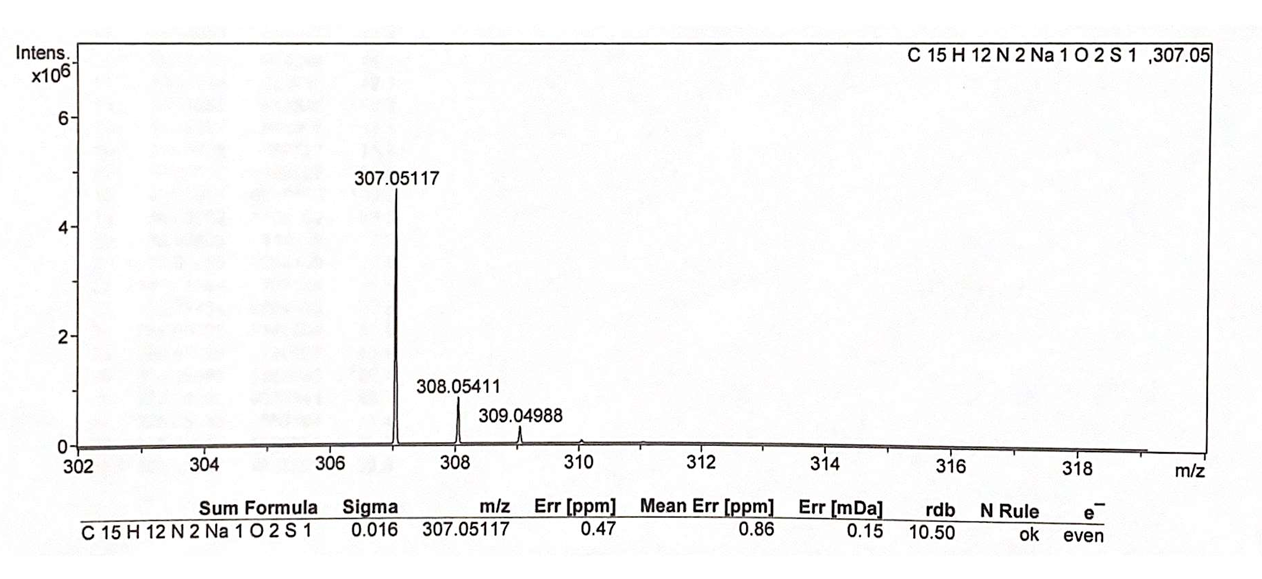
**

**The** **HRMS (ESI) spectrum of compound 8.**

**Supplementary data charts (S# 5)**

**Compounds (9)**


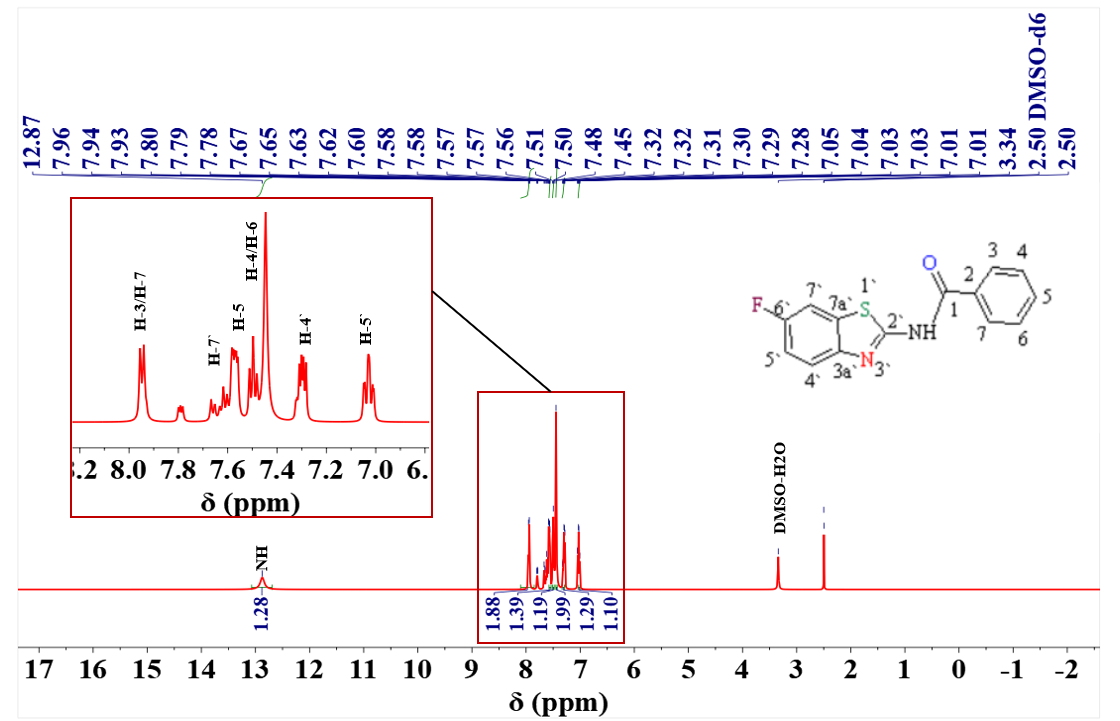


**The ^1^H-NMR spectrum of compound 9.**


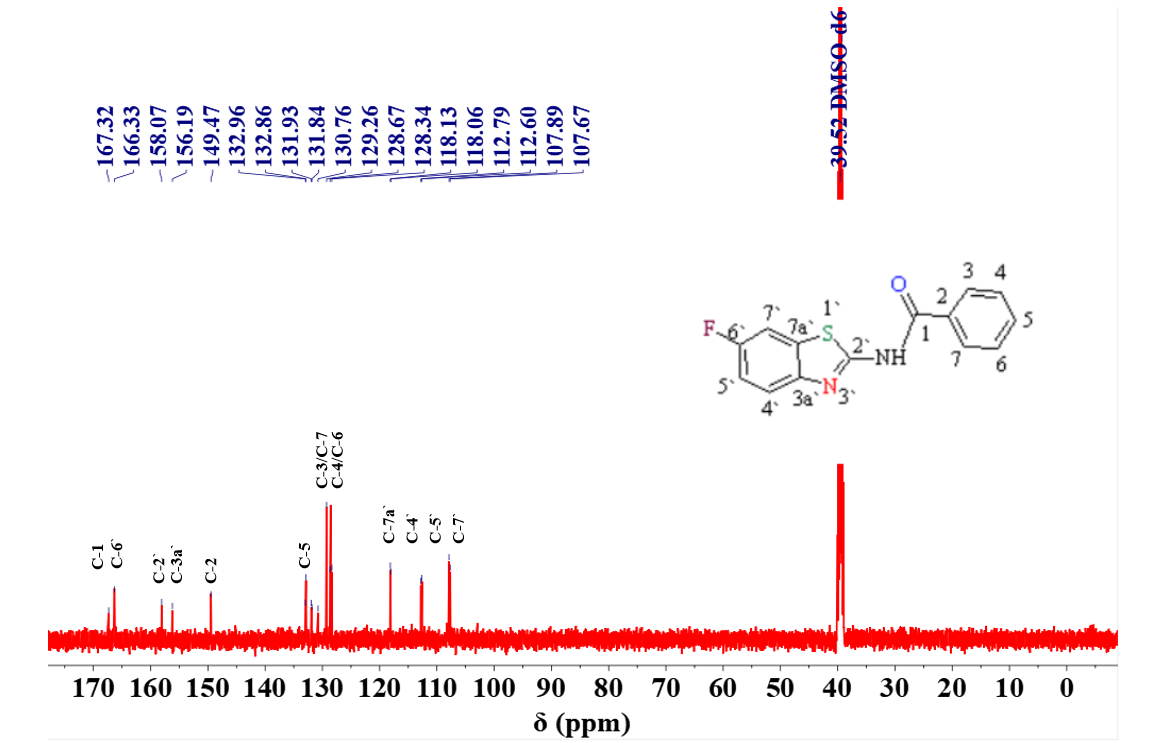


**The** **^13^C-NMR spectrum of compound 9.**


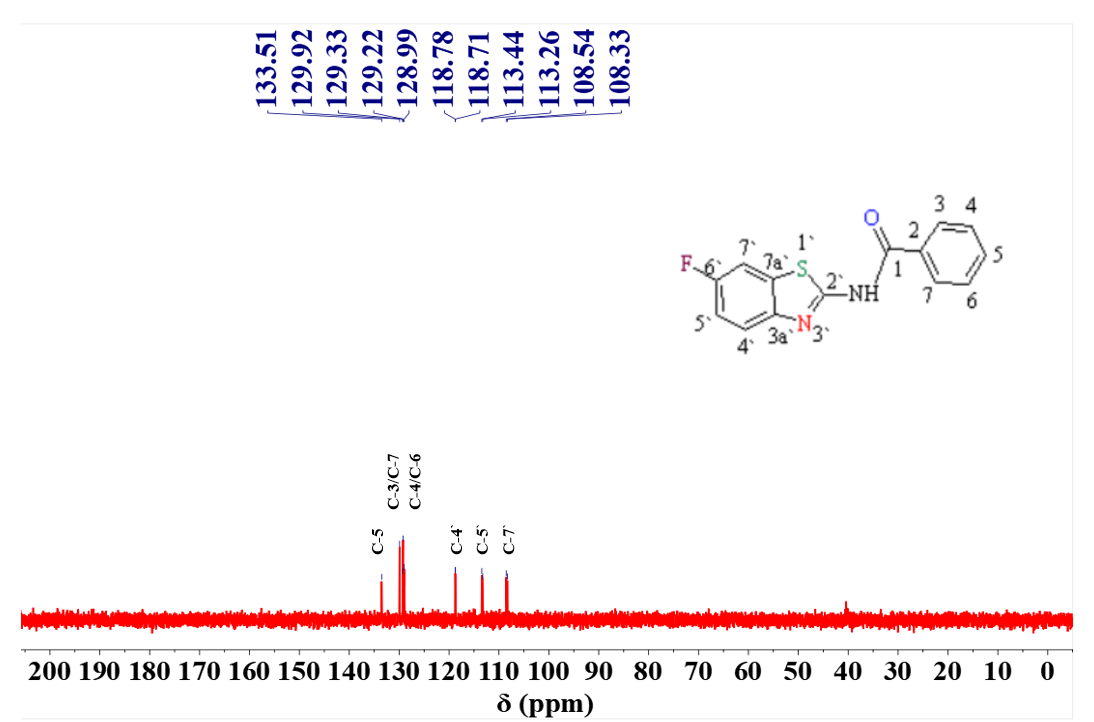


**The** **DEPT** **^13^C-NMR spectrum of compound 9.**

**
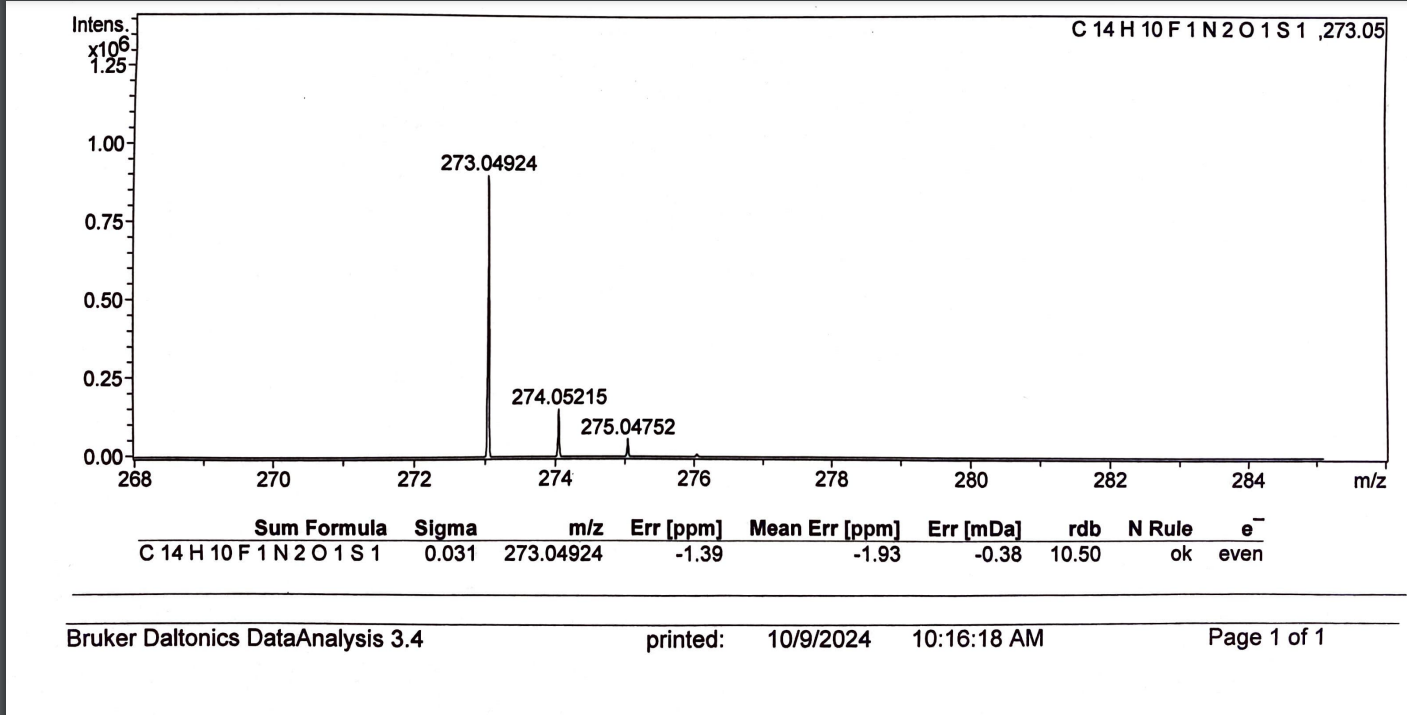
**

**The** **HRMS (ESI) spectrum of compound 9.**

**Supplementary data charts (S# 6)**

**Compounds (10)**


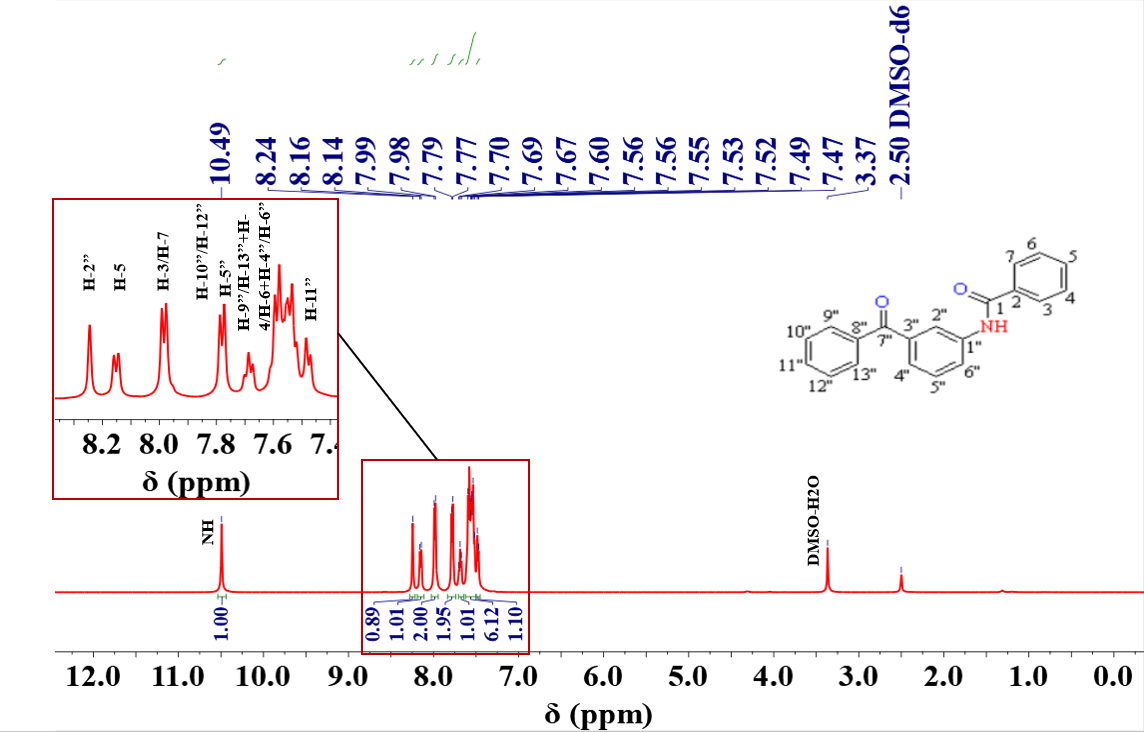


**The ^1^H-NMR spectrum of compound 10.**


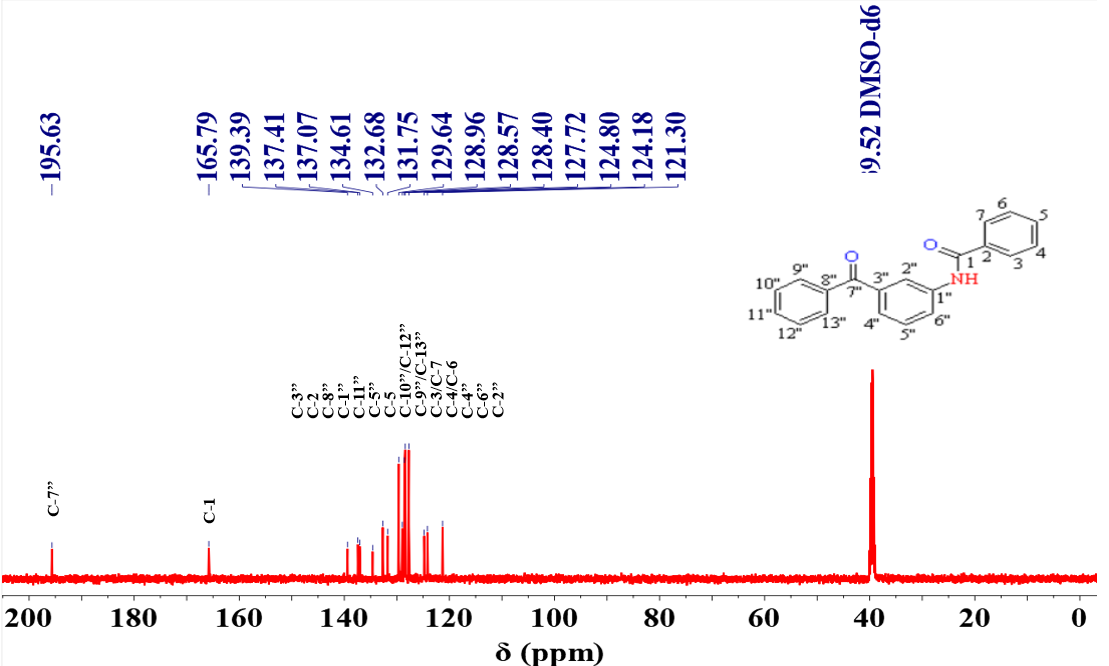


**The** **^13^C-NMR spectrum of compound 10.**


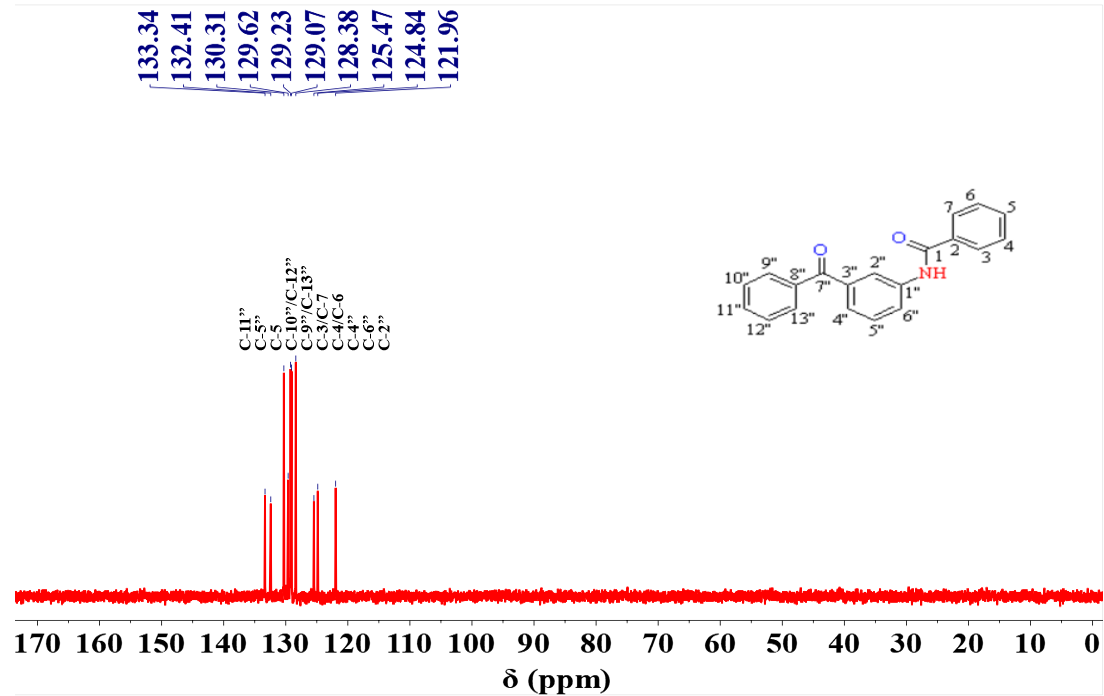


**The** **DEPT** **^13^C-NMR spectrum of compound 10.**


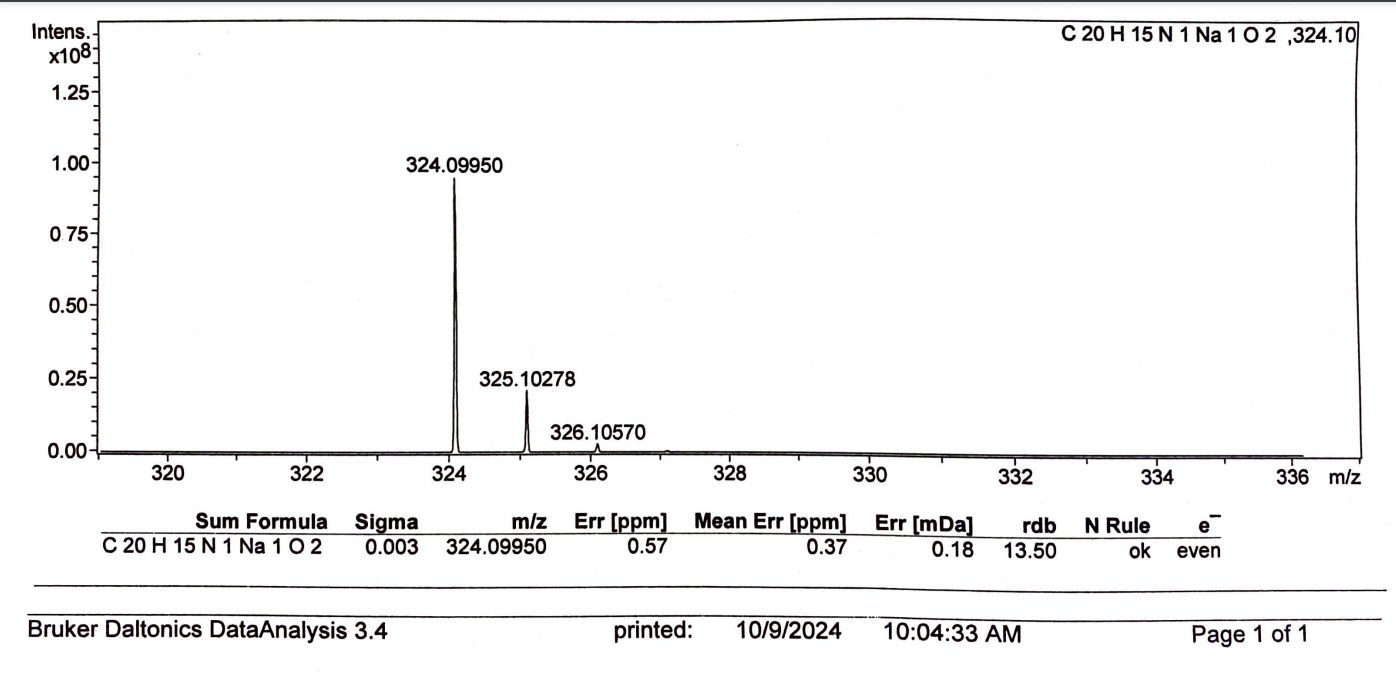


**The** **HRMS (ESI) spectrum of compound 10.**

**Supplementary data charts (S# 7)**

**compounds (11)**

**
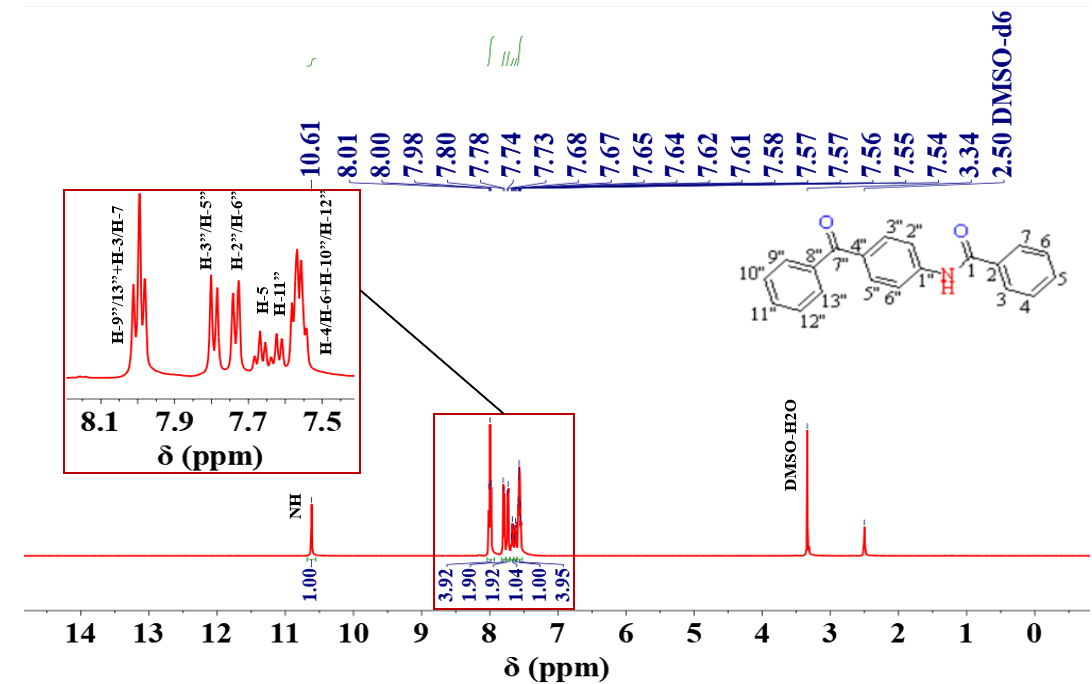
**

**The ^1^H-NMR spectrum of compound 11.**

**
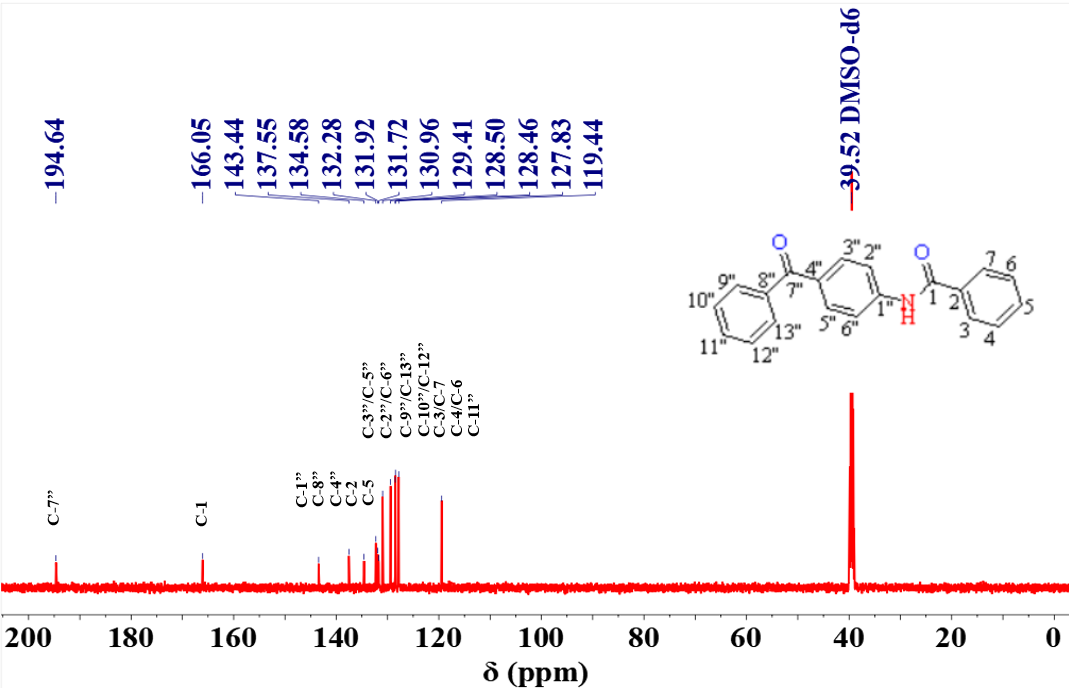
**

**The** **^13^C-NMR spectrum of compound 11.**

**
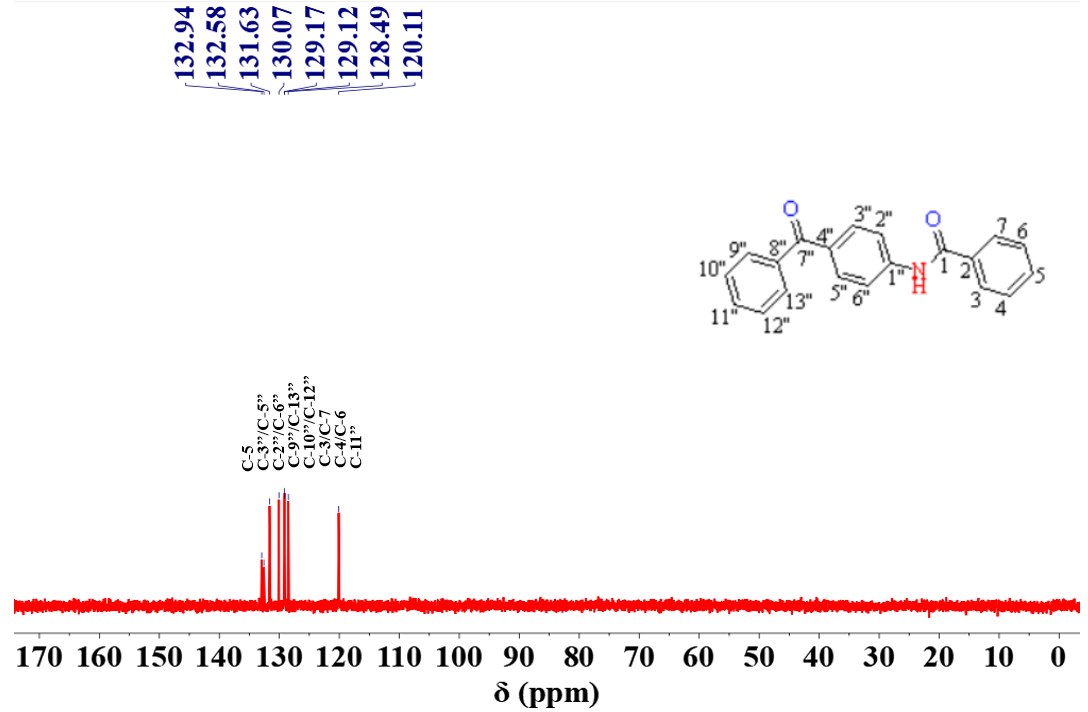
**

**The** **DEPT** **^13^C-NMR spectrum of compound 11.**

**Supplementary data charts (S# 8)**

**Compounds (12)**


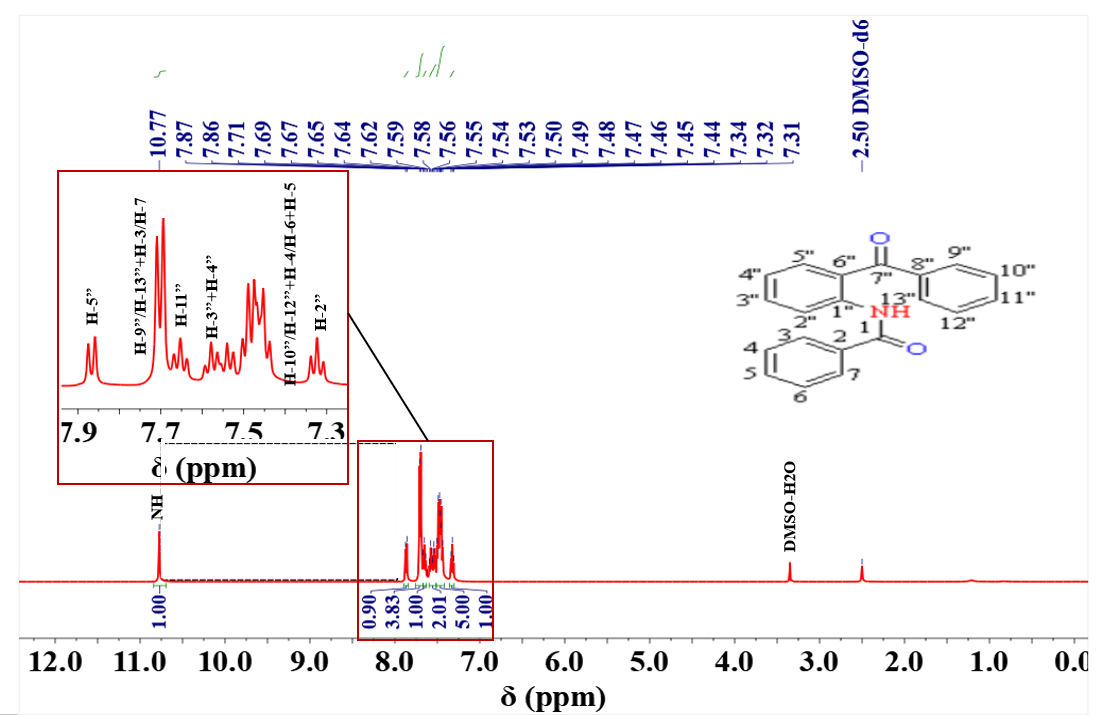


**The ^1^H-NMR spectrum of compound 12.**


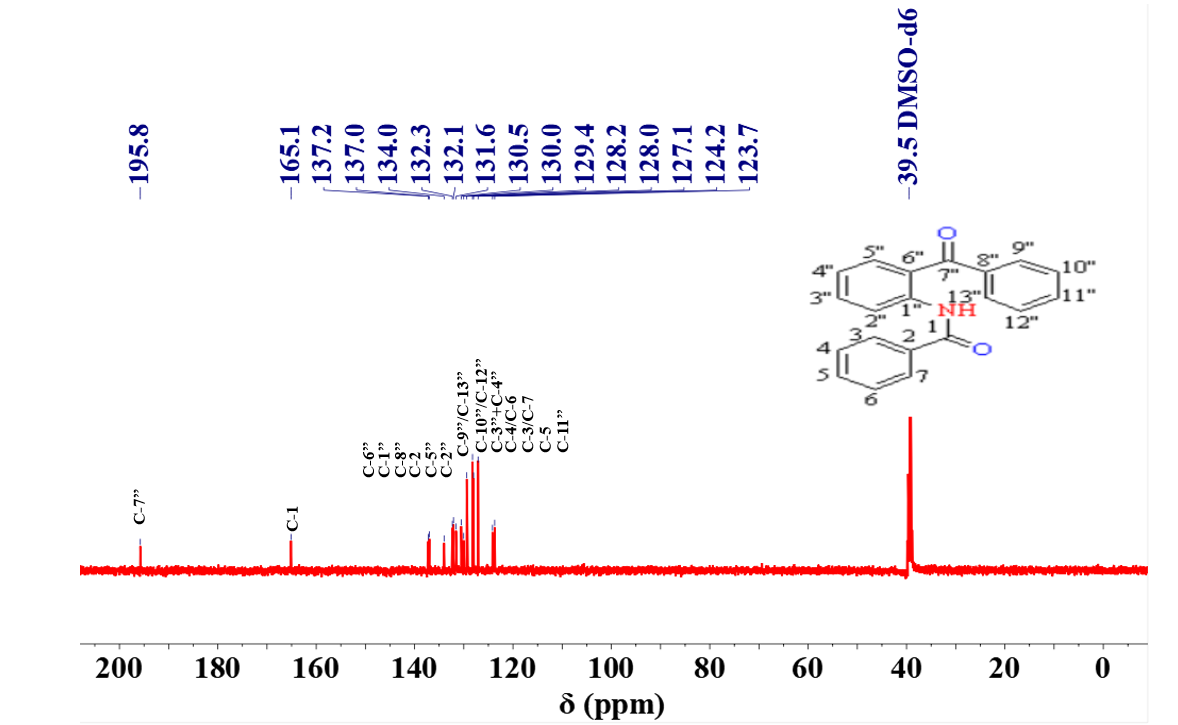


**The** **^13^C-NMR spectrum of compound 12.**

**
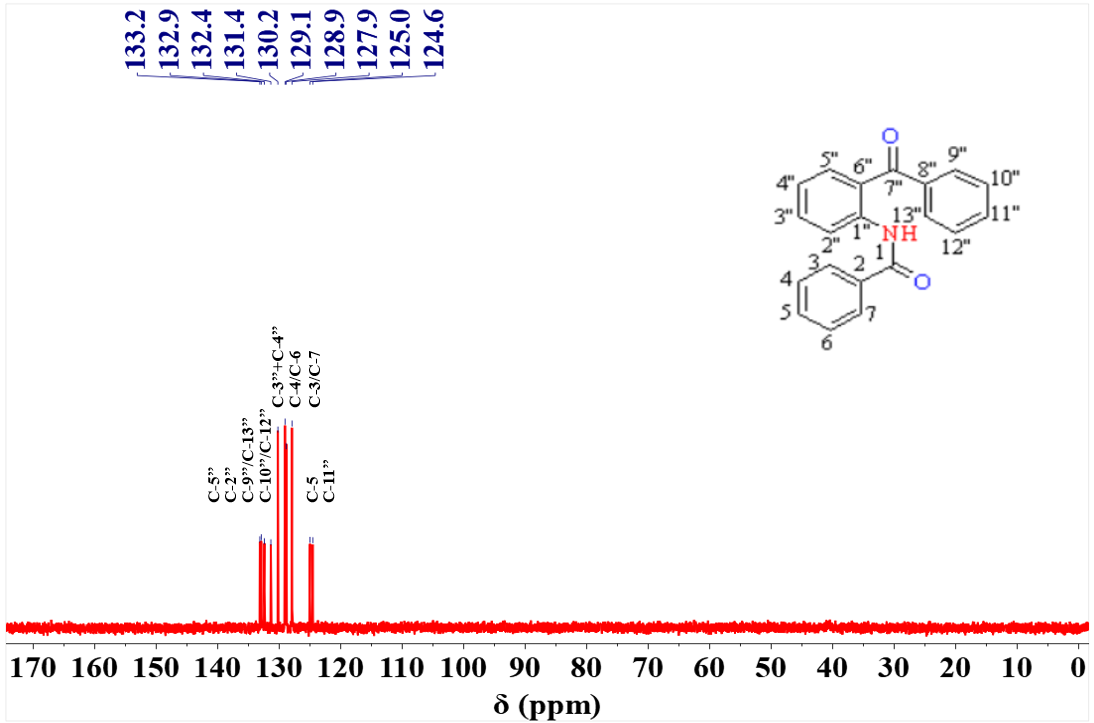
**

**The** **DEPT** **^13^C-NMR spectrum of compound 12.**


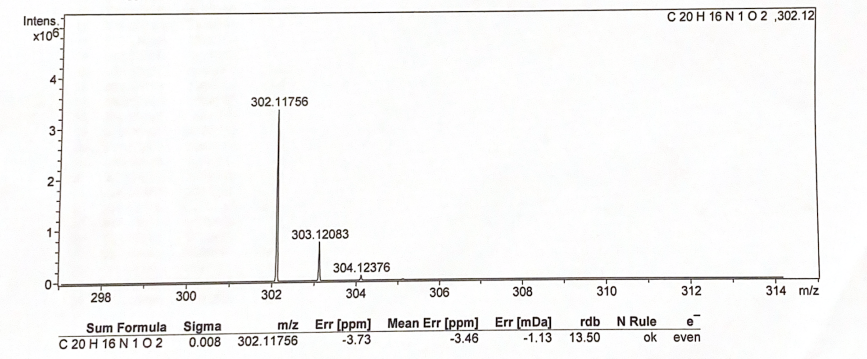


**The** **HRMS (ESI) spectrum of compound 12.**

**Supplementary data charts (S# 9)**

**Compounds (13)**


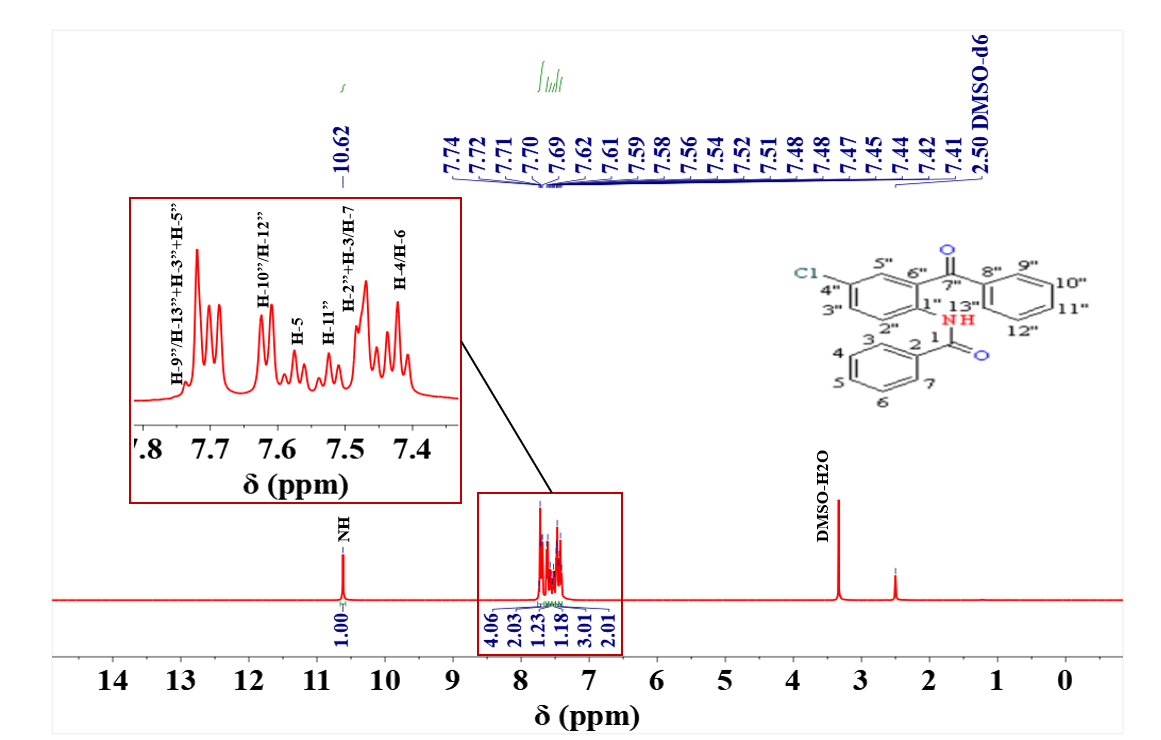


**The ^1^H-NMR spectrum of compound 13.**


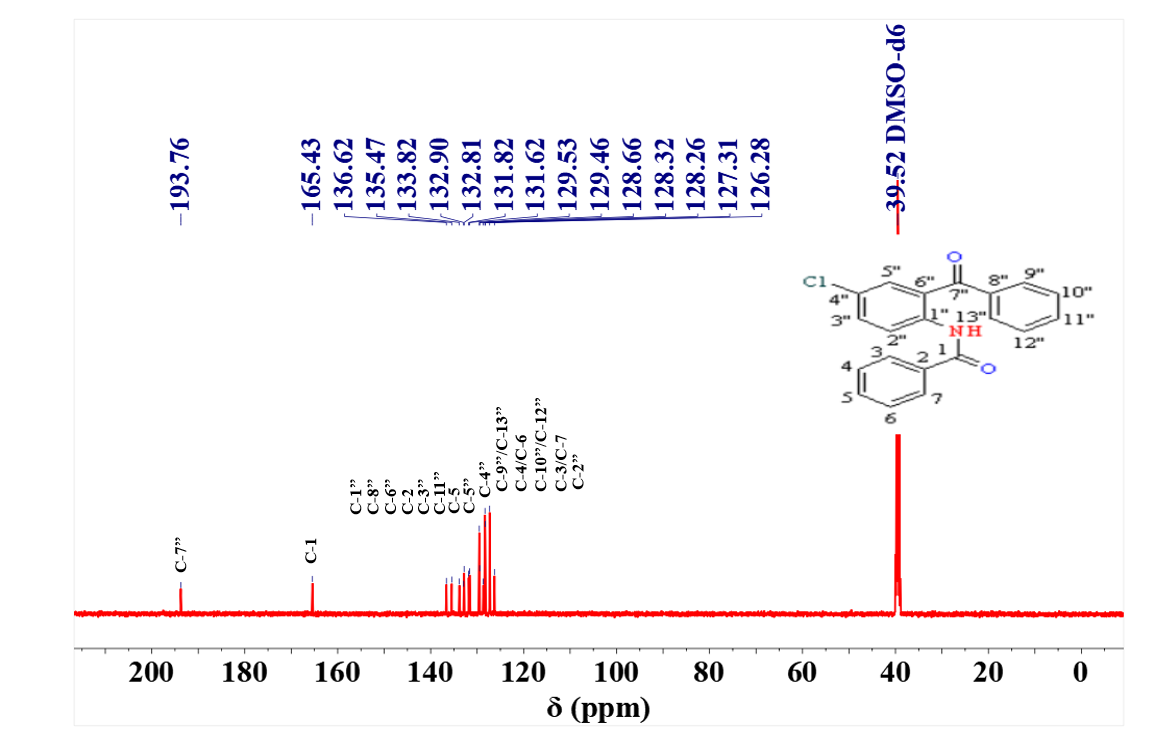


**The** **^13^C-NMR spectrum of compound 13.**

**
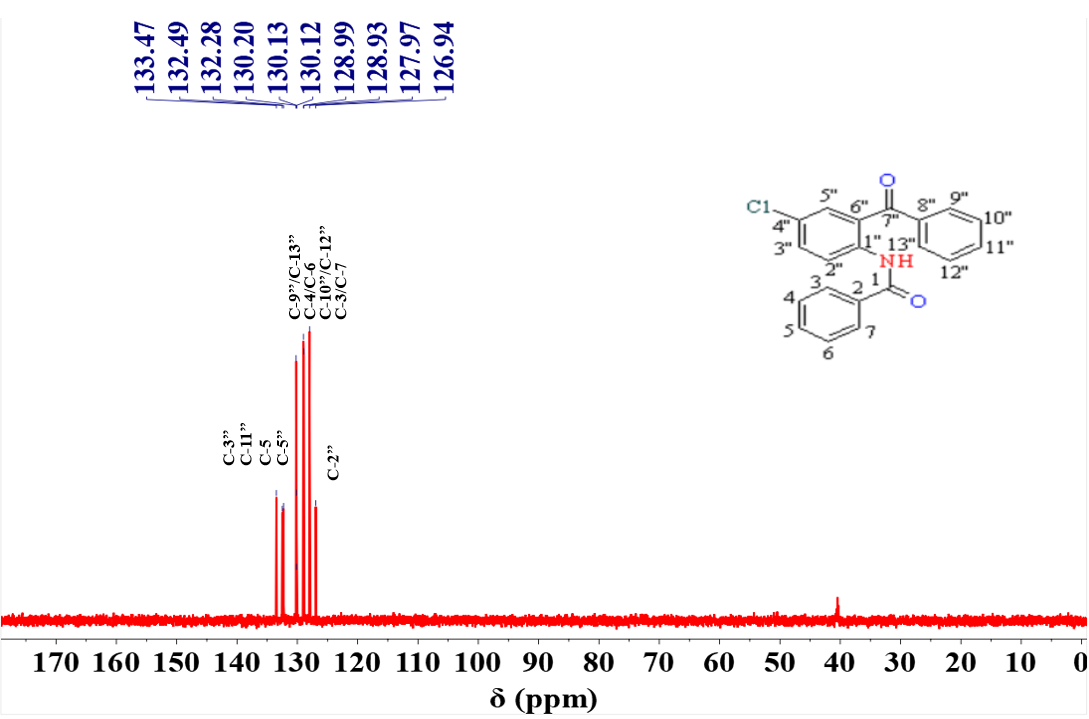
**

**The** **DEPT** **^13^C-NMR spectrum of compound 13.**


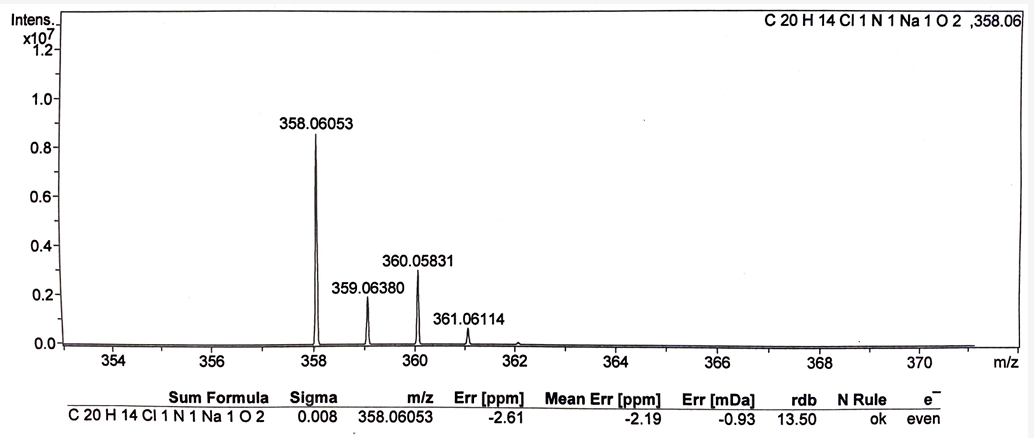


**The** **HRMS (ESI) spectrum of compound 13.**

**Supplementary data charts (S# 10)**

**Compounds (14)**


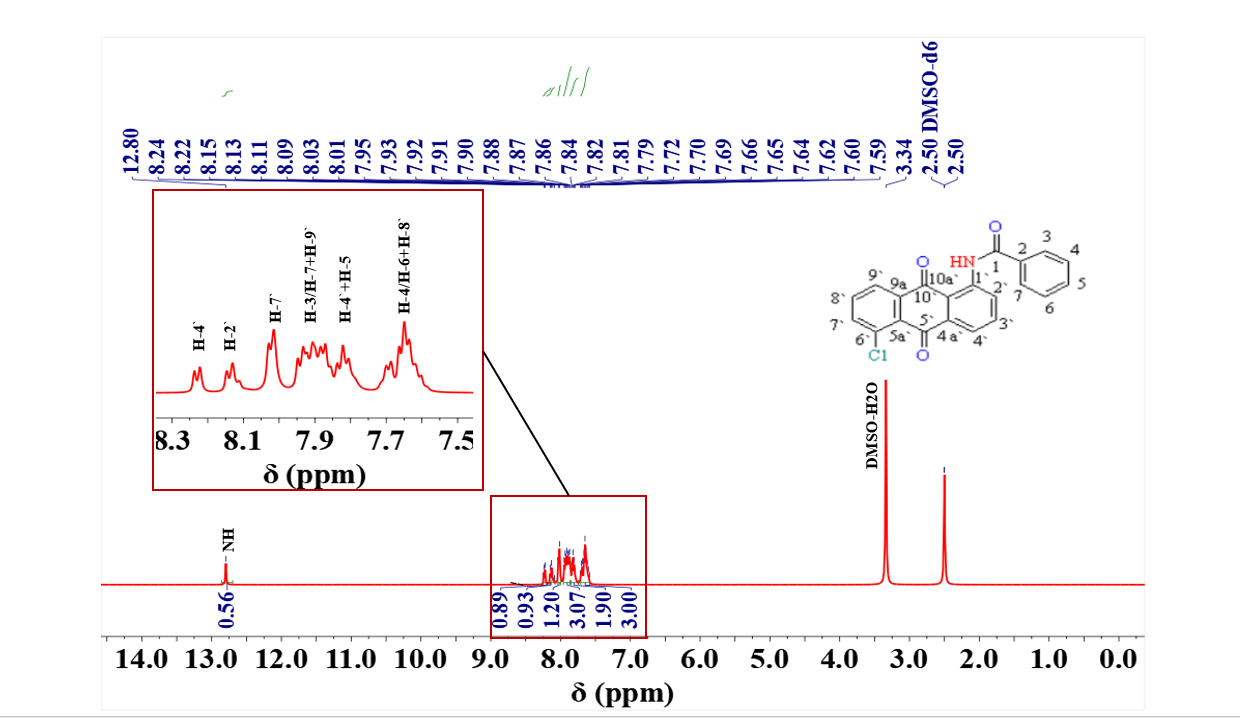


**The ^1^H-NMR spectrum of compound 14.**


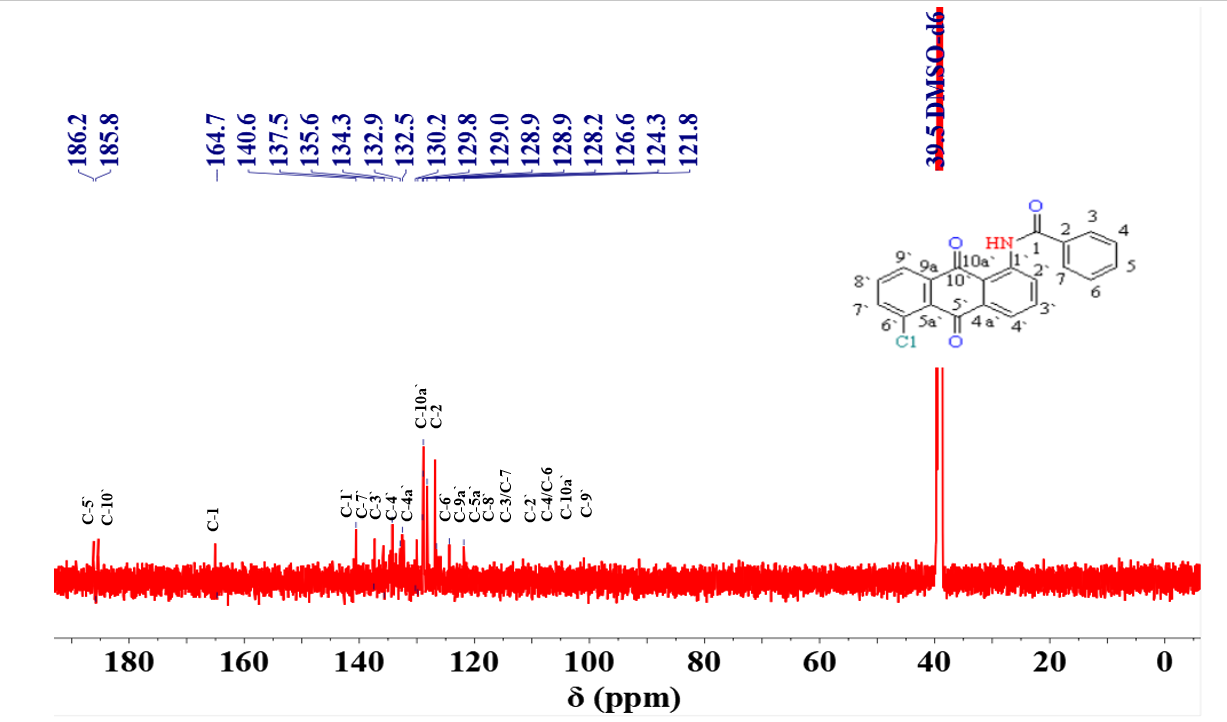


**The** **^13^C-NMR spectrum of compound 14.**

**
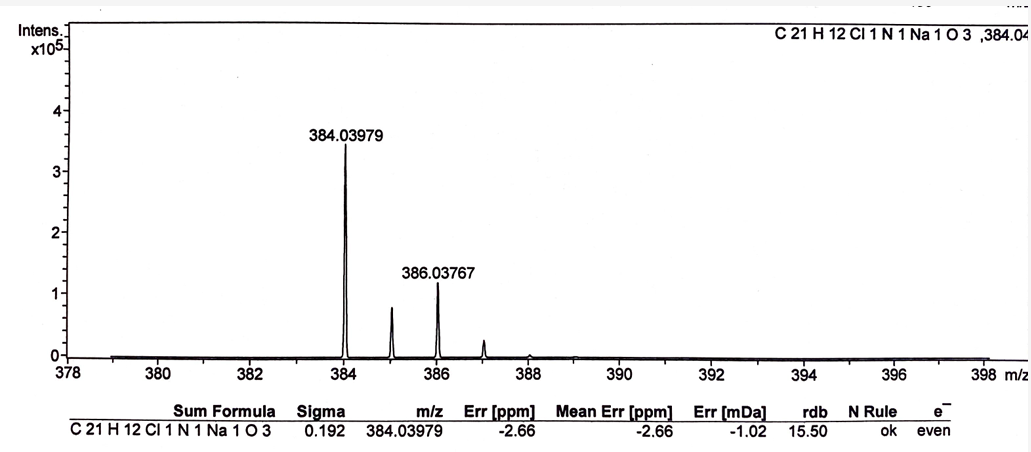
**

**The** **HRMS (ESI) spectrum of compound 14.**

**Supplementary data charts (S# 11)**

**compounds (15)**


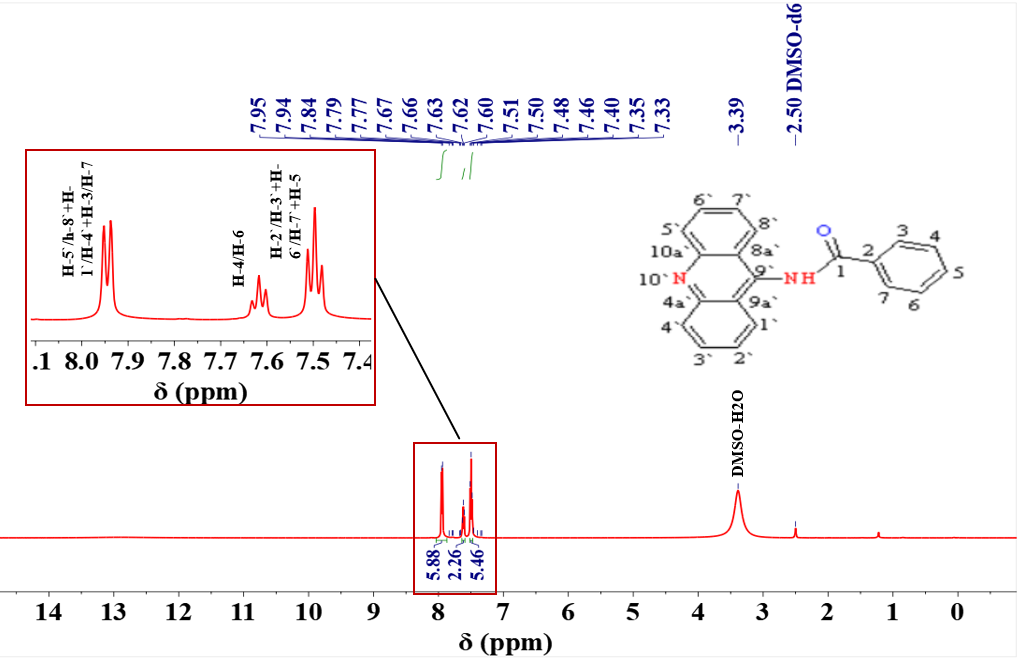


**The ^1^H-NMR spectrum of compound 15.**


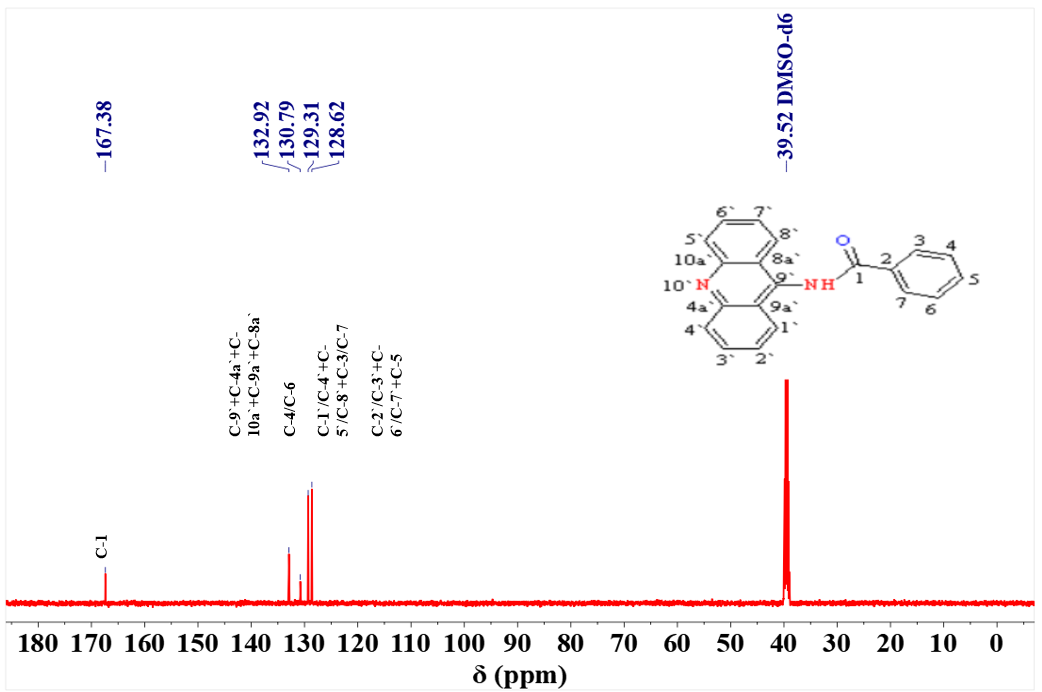


**The** **^13^C-NMR spectrum of compound 15.**


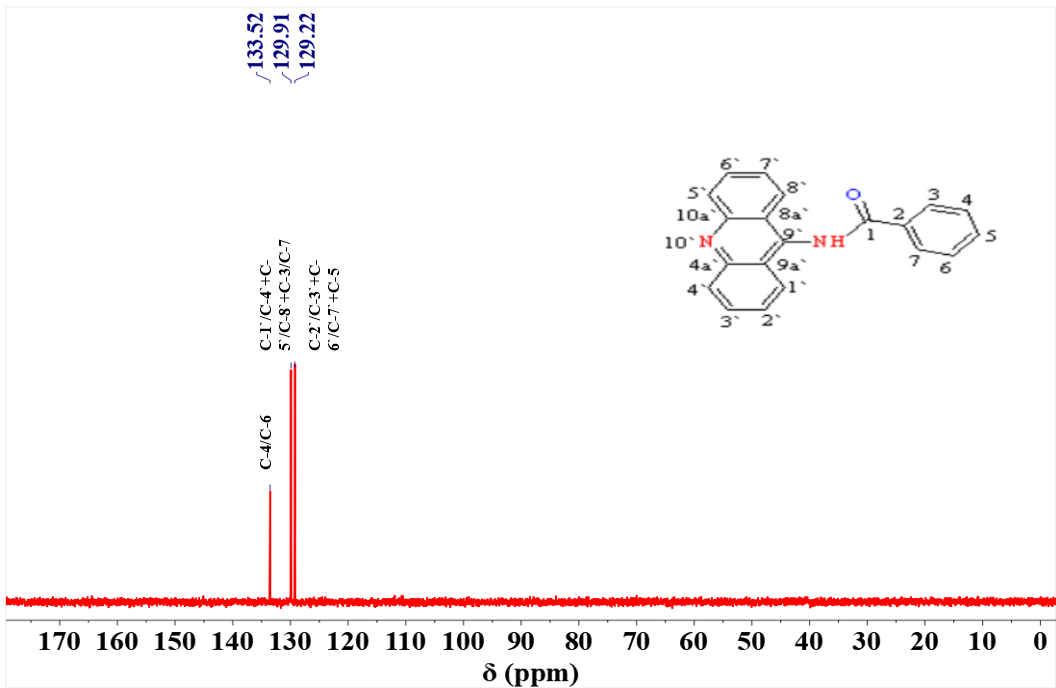


**The** **DEPT** **^13^C-NMR spectrum of compound 15.**

**Supplementary data charts (S# 12)**

**compounds (20)**


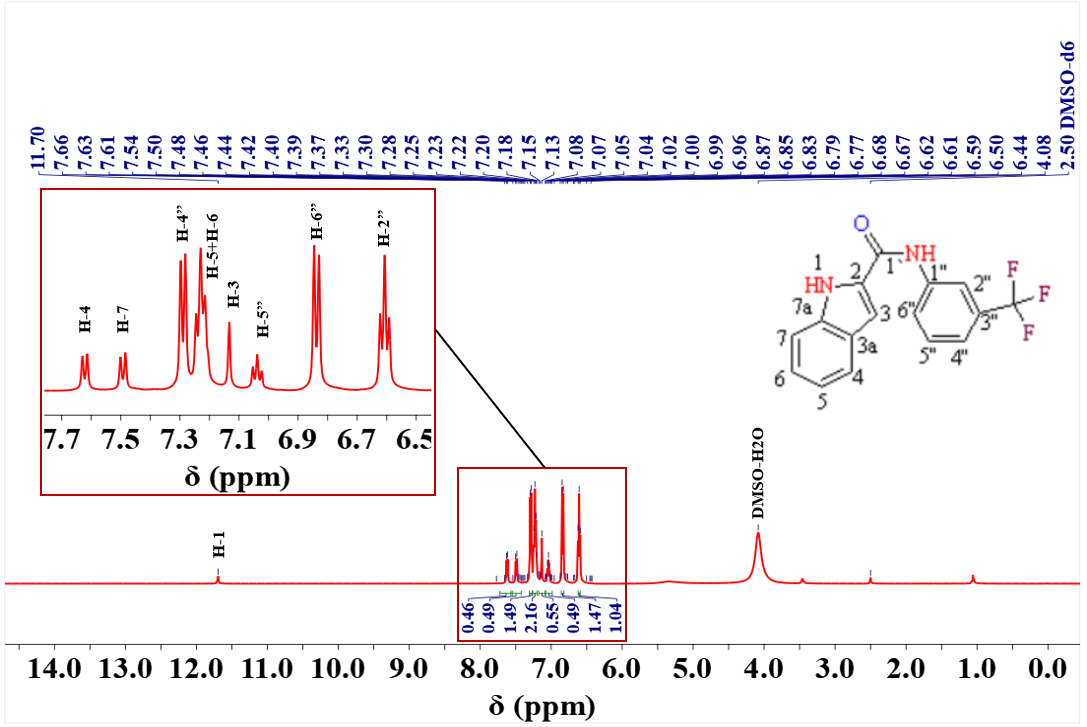


**The ^1^H-NMR spectrum of compound 20.**


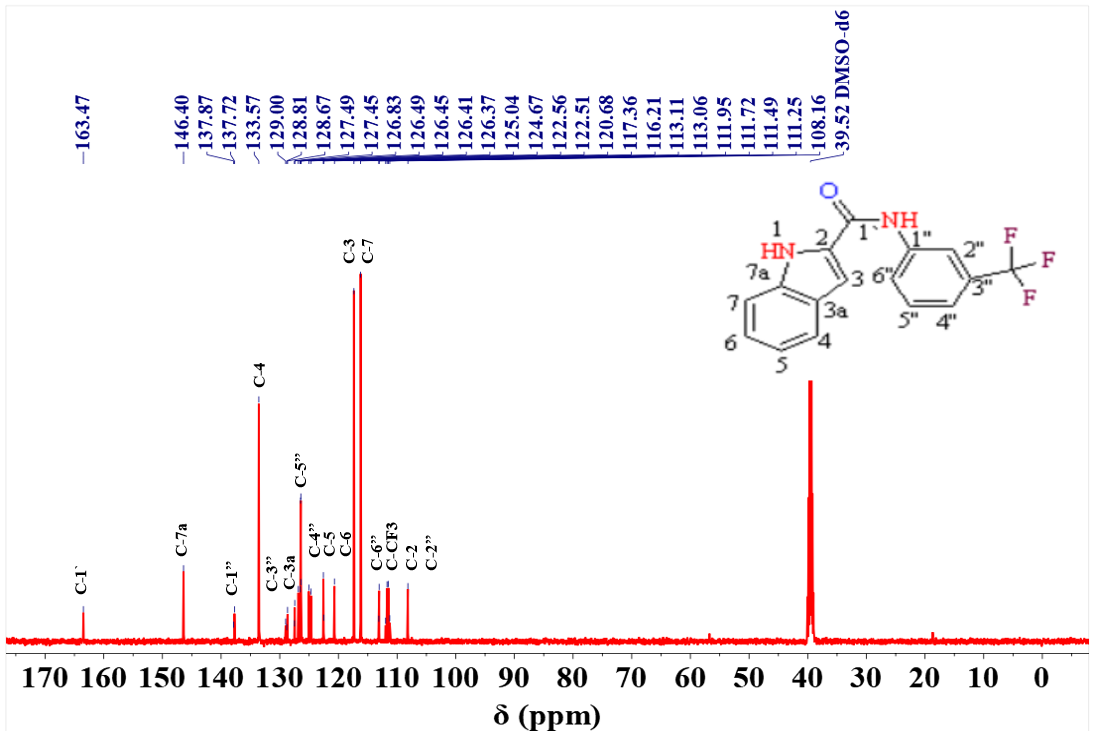


**The** **^13^C-NMR spectrum of compound 20.**


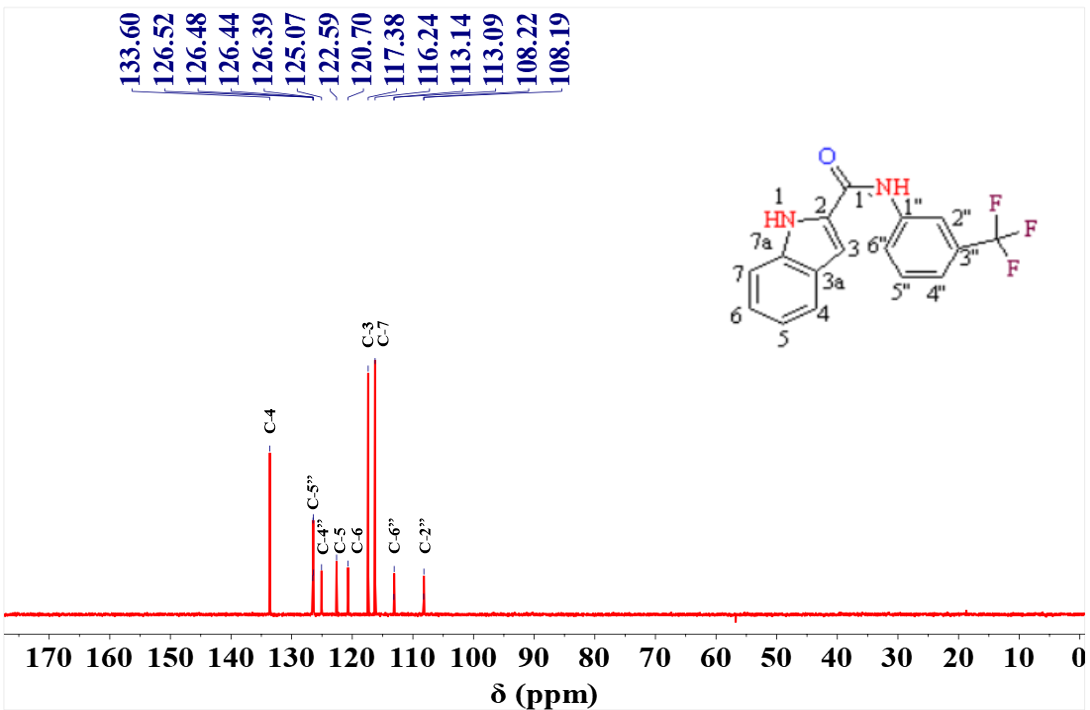


**The** **DEPT** **^13^C-NMR spectrum of compound 20.**

**Supplementary data charts (S# 13)**

**Compound (21)**


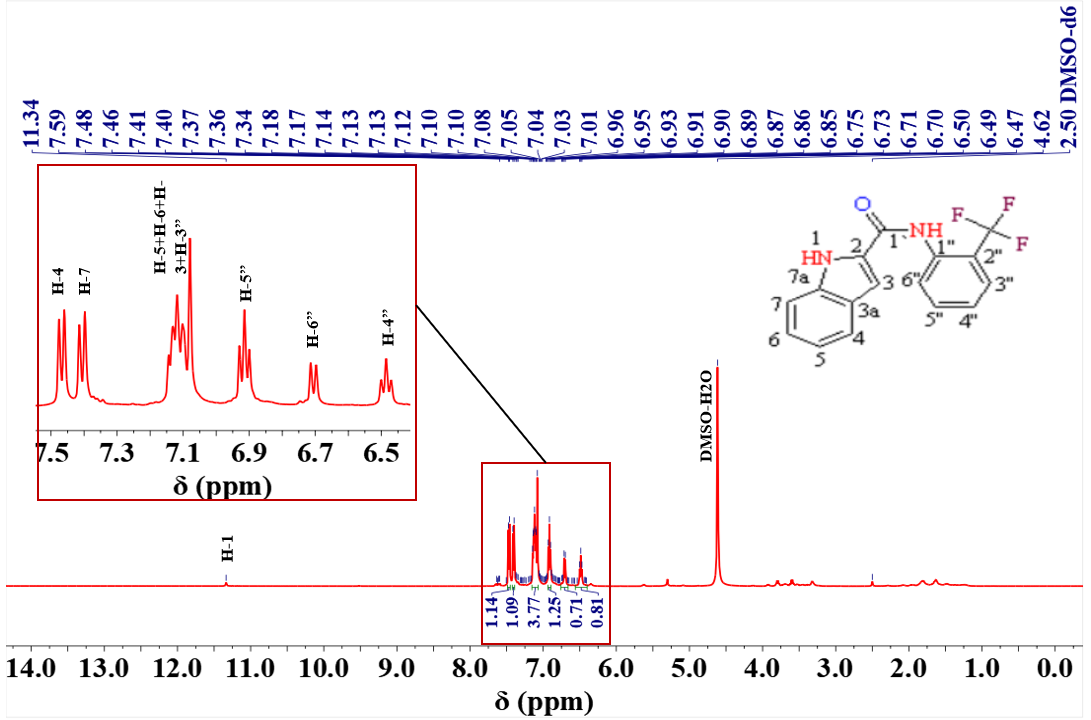


**The ^1^H-NMR spectrum of compound 21.**


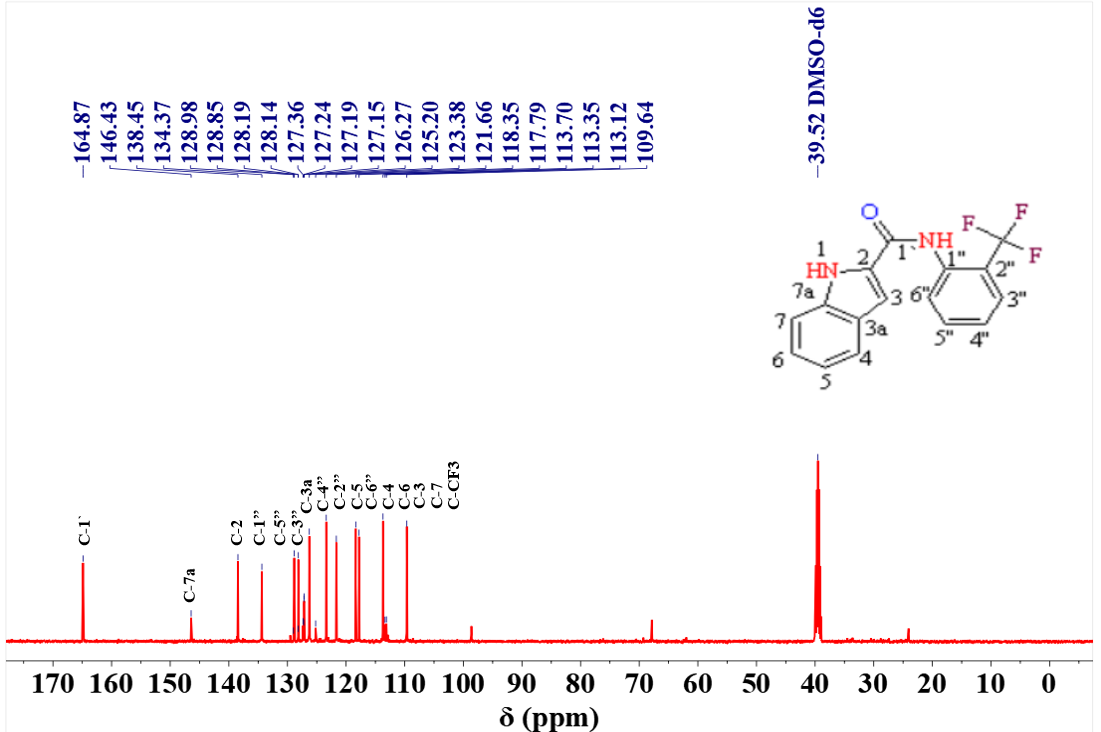


**The** **^13^C-NMR spectrum of compound 21.**

**Supplementary data charts (S# 14)**

**compounds (21)**

**
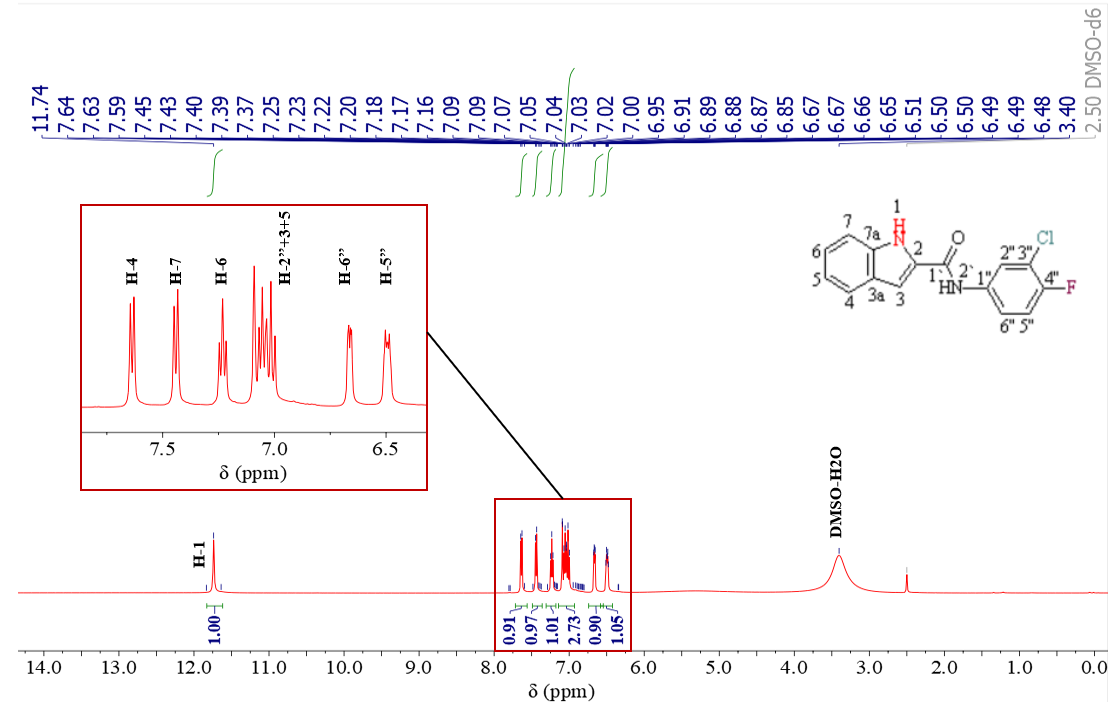
**

**The ^1^H-NMR spectrum of compound 21.**


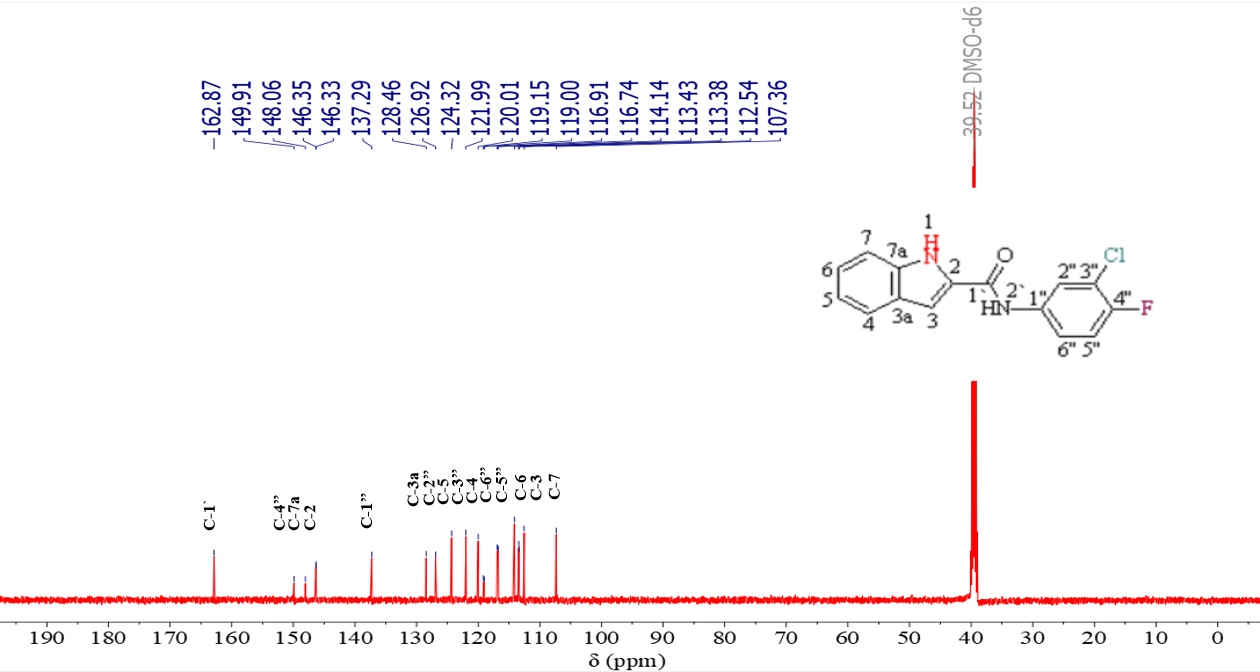


**The** **^13^C-NMR spectrum of compound 21.**

**
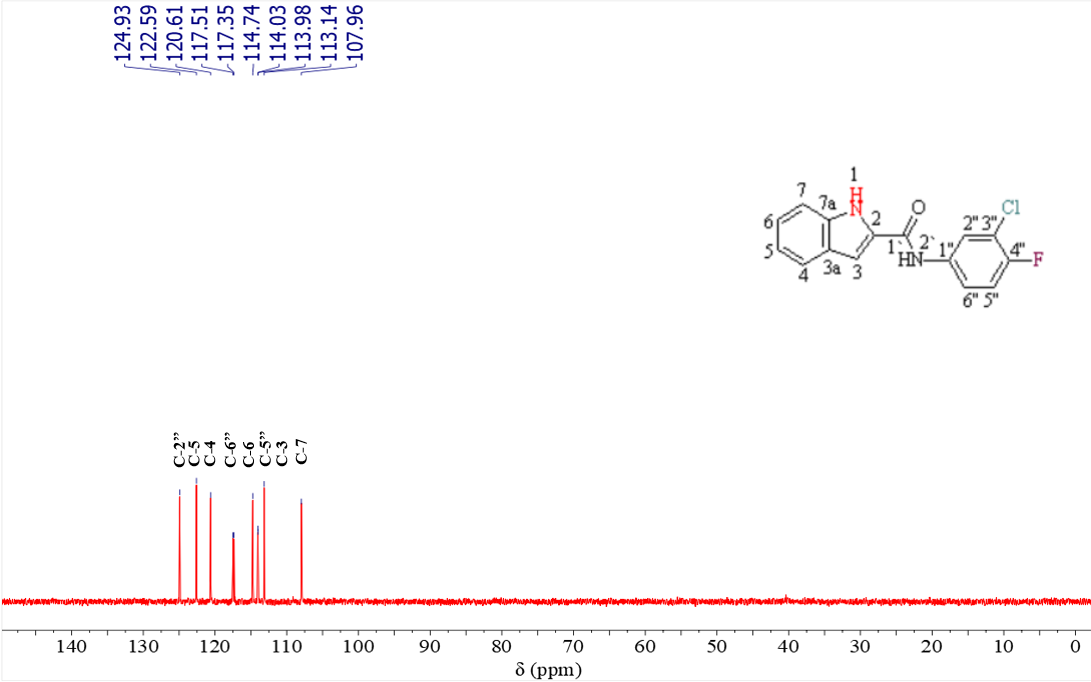
**

**The** **DEPT** **^13^C-NMR spectrum of compound 21.**

**Supplementary data charts (S# 15)**

**compounds (23)**


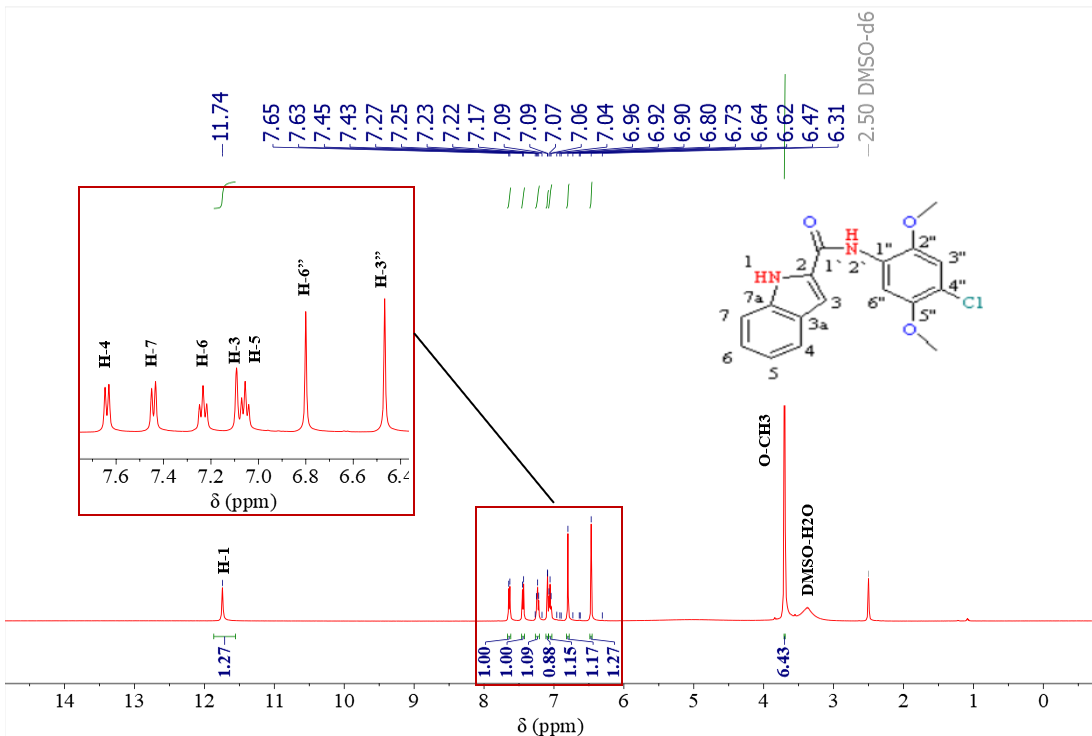


**The ^1^H-NMR spectrum of compound 23.**
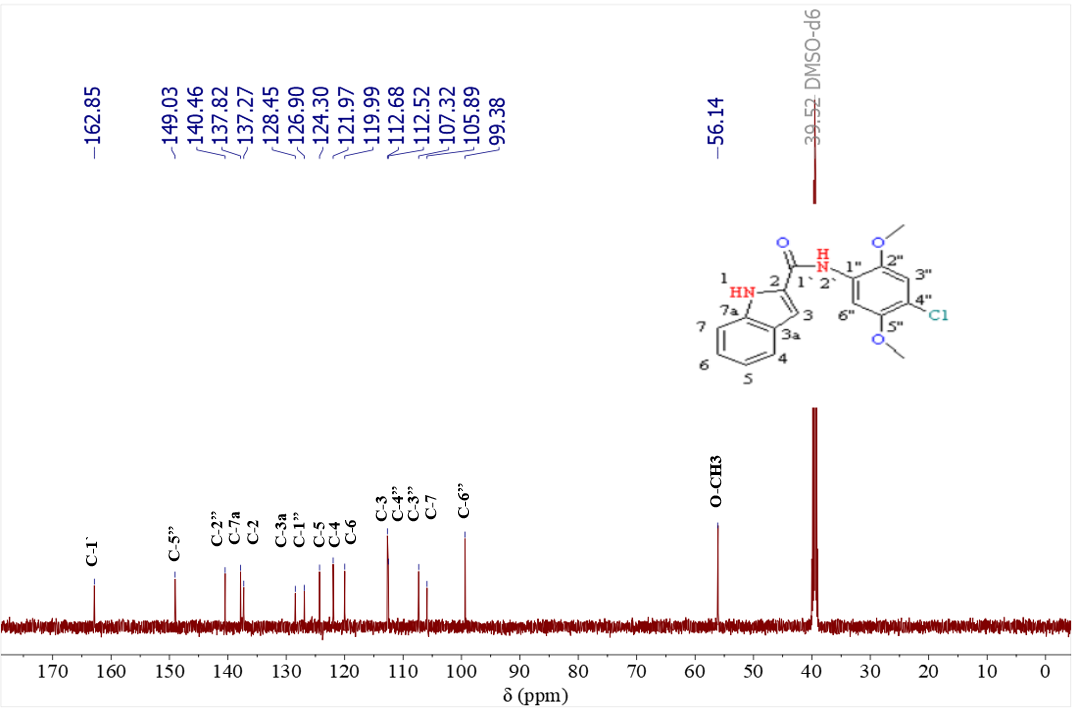


**The** **^13^C-NMR spectrum of compound 23.**


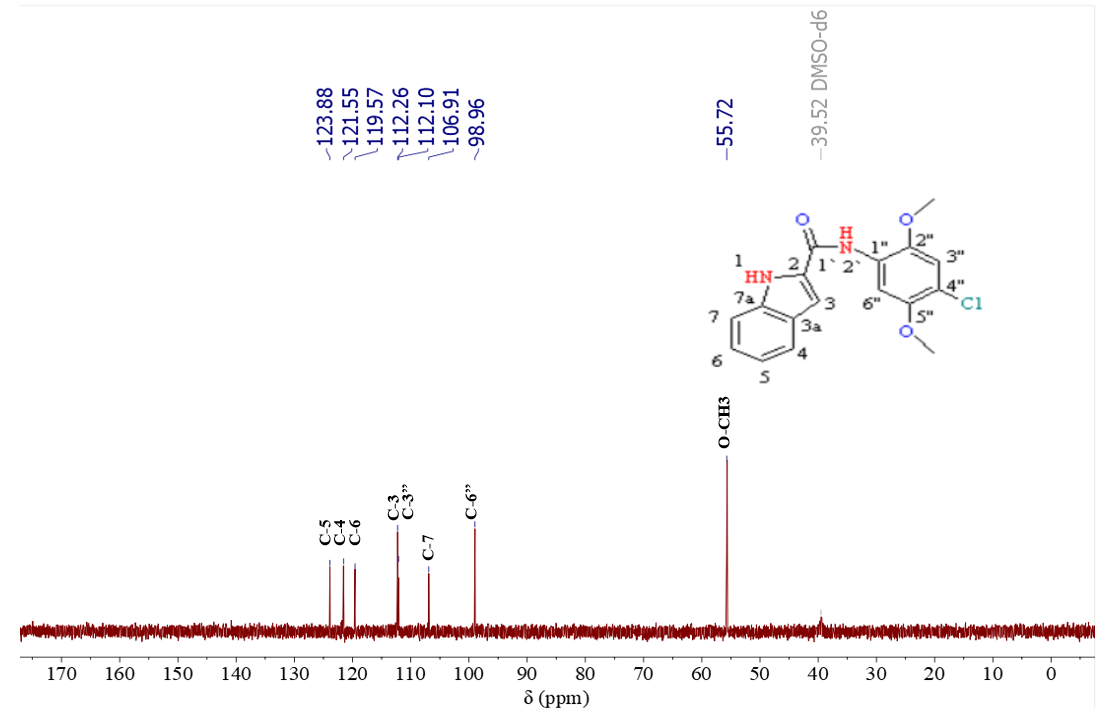


**The** **DEPT** **^13^C-NMR spectrum of compound 23.**

**Supplementary data charts (S# 16)**

**compounds (24)**


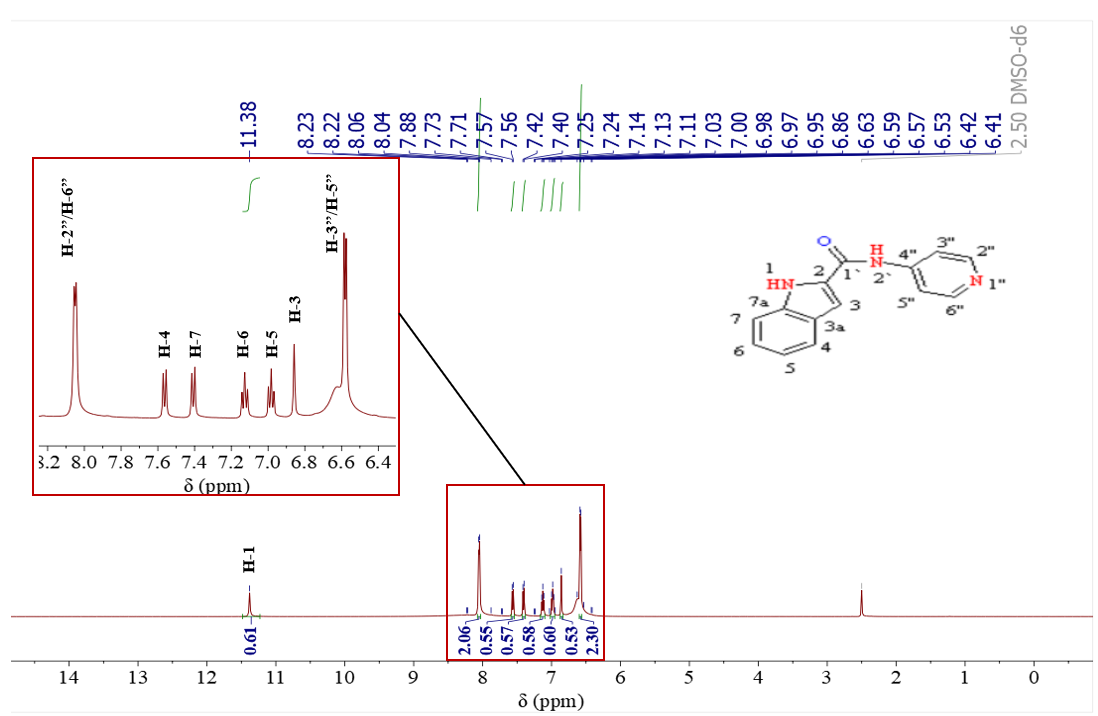


**The ^1^H-NMR spectrum of compound 24.**
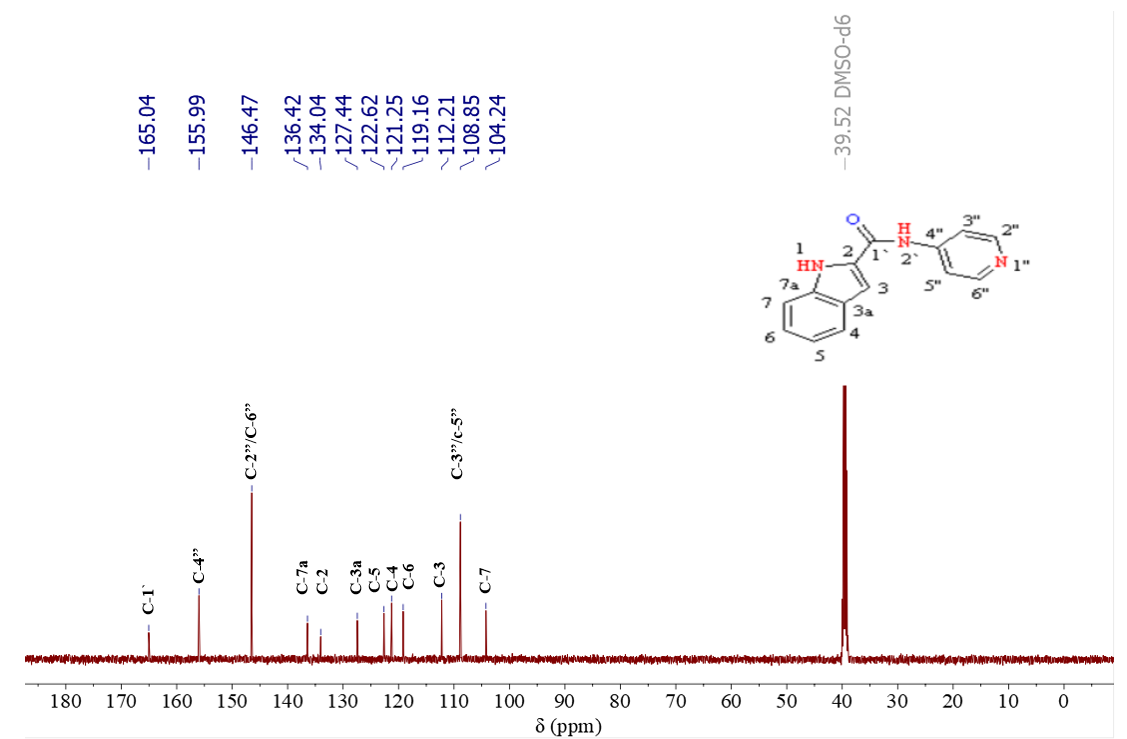


**The** **^13^C-NMR spectrum of compound 24.**


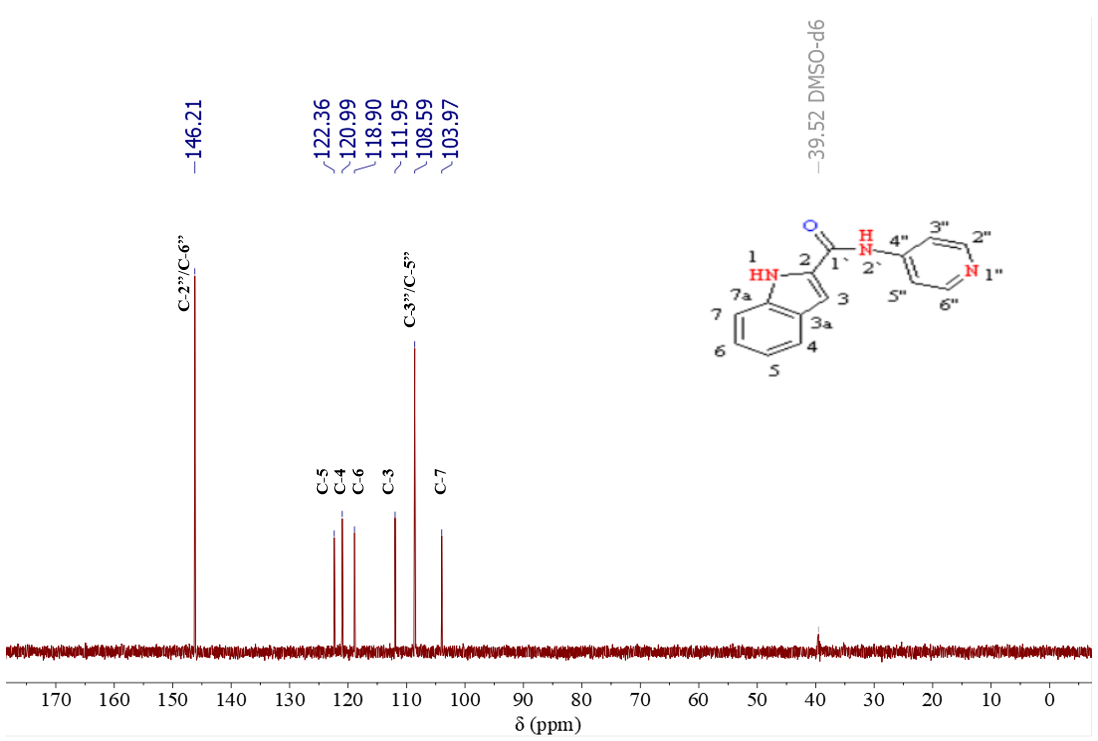


**The** **DEPT** **^13^C-NMR spectrum of compound 24.**

**Supplementary data charts (S# 17)**

**compounds (25)**


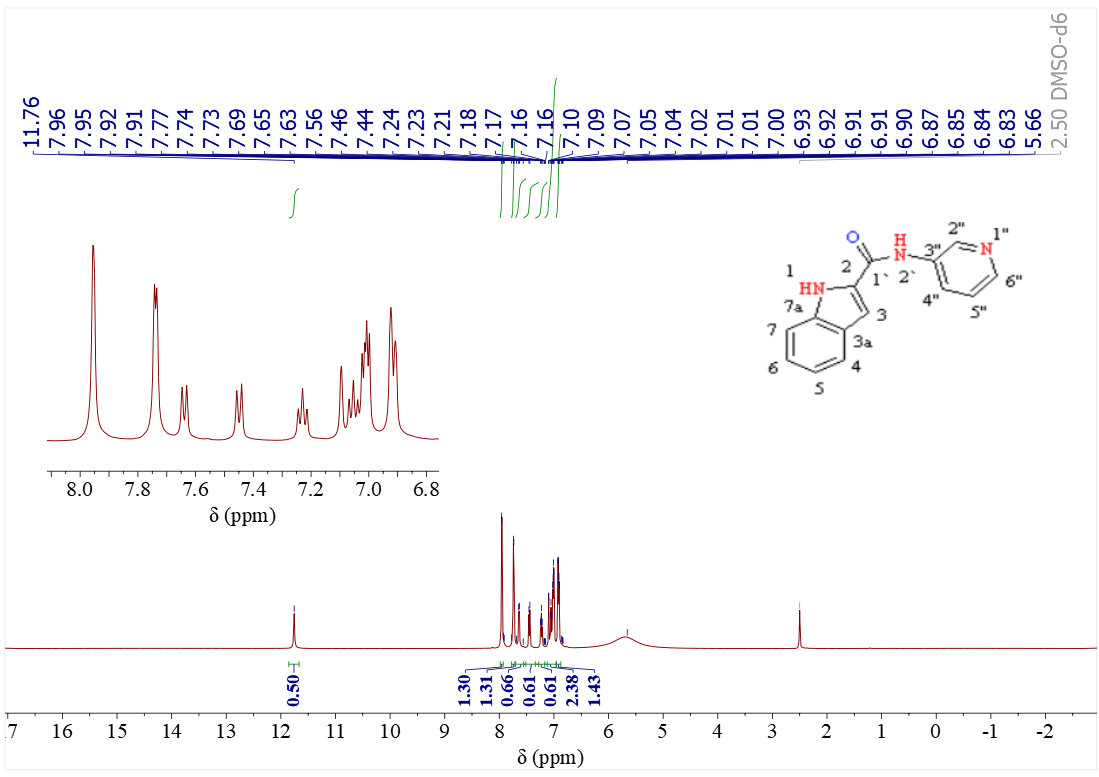


**The ^1^H-NMR spectrum of compound 25.**
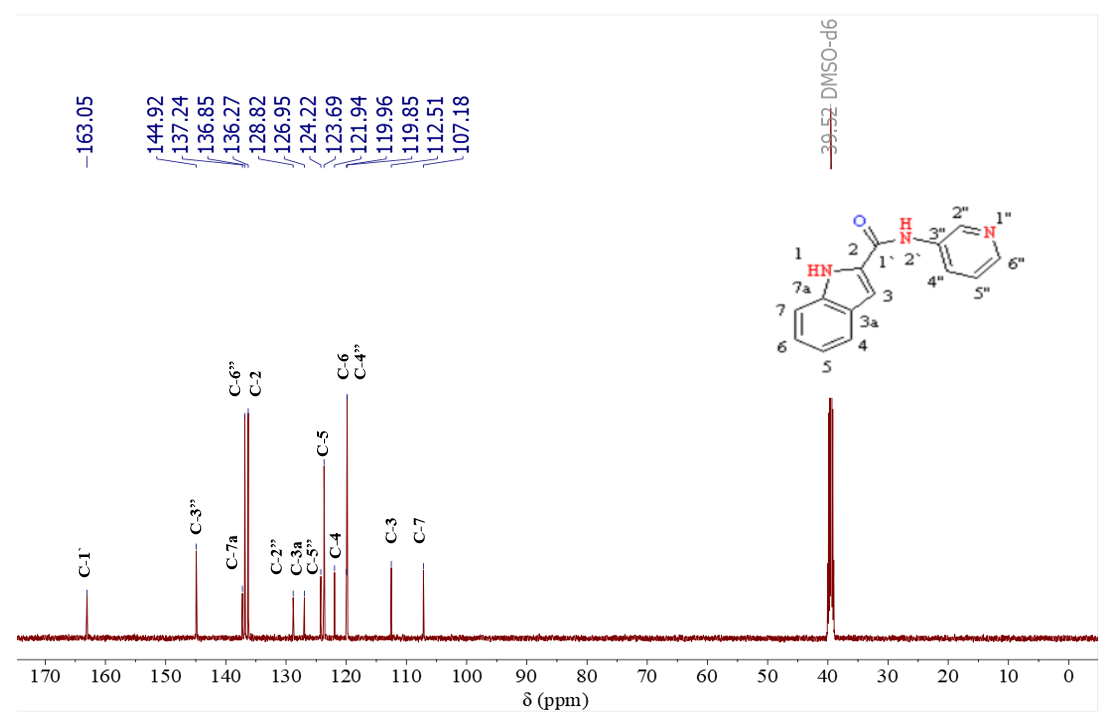


**The** **^13^C-NMR spectrum of compound 25.**


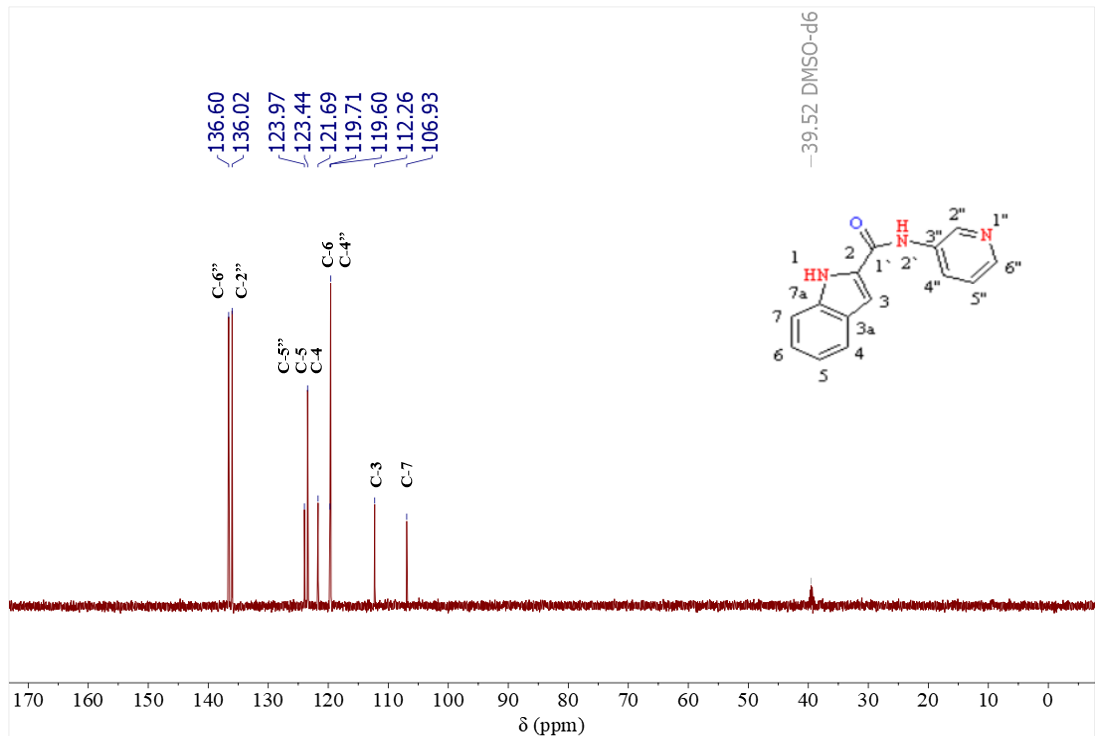


**The** **DEPT** **^13^C-NMR spectrum of compound 25.**

**Supplementary data charts (S# 18)**

**compounds (26)**

**
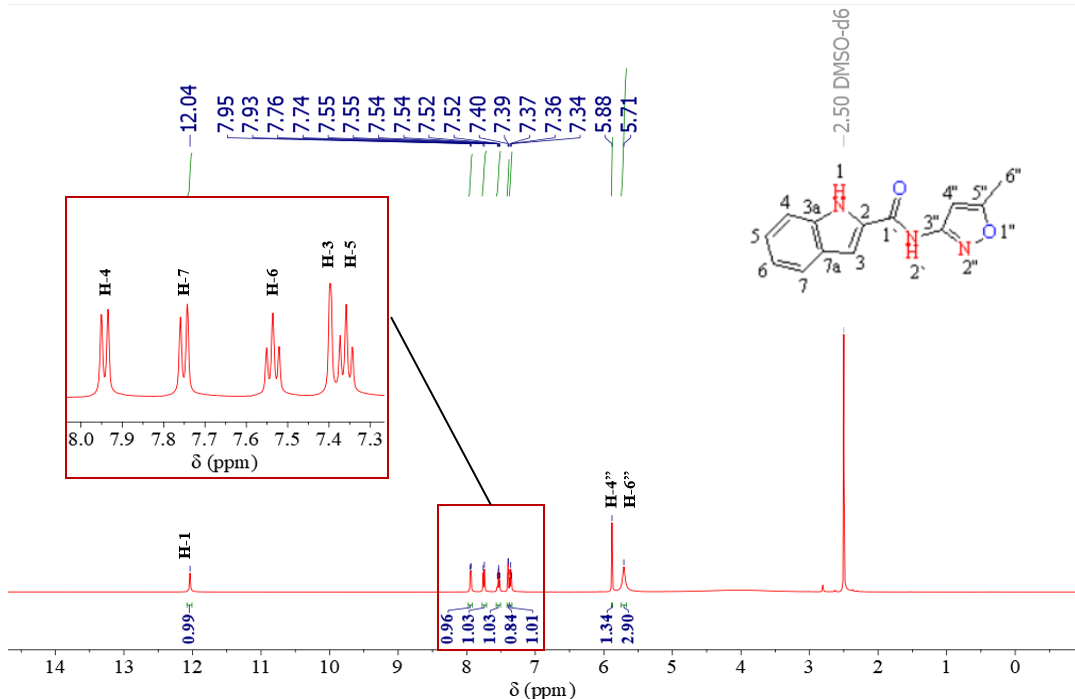
**

**The ^1^H-NMR spectrum of compound 26.**

**
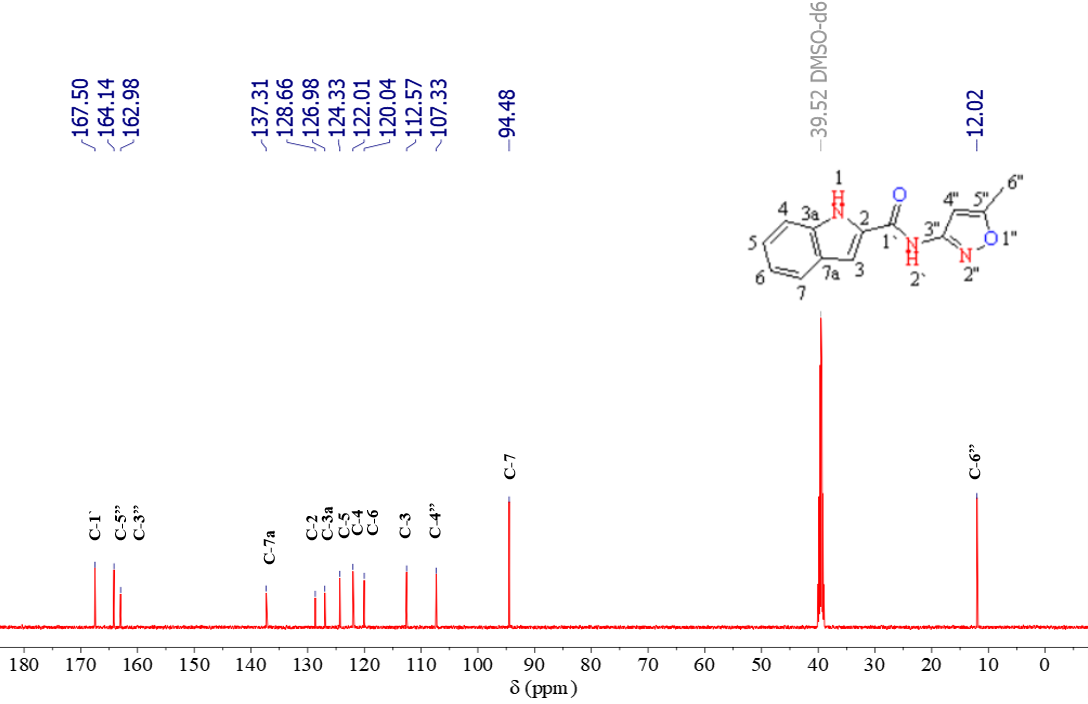
**

**The** **^13^C-NMR spectrum of compound 26.**

**
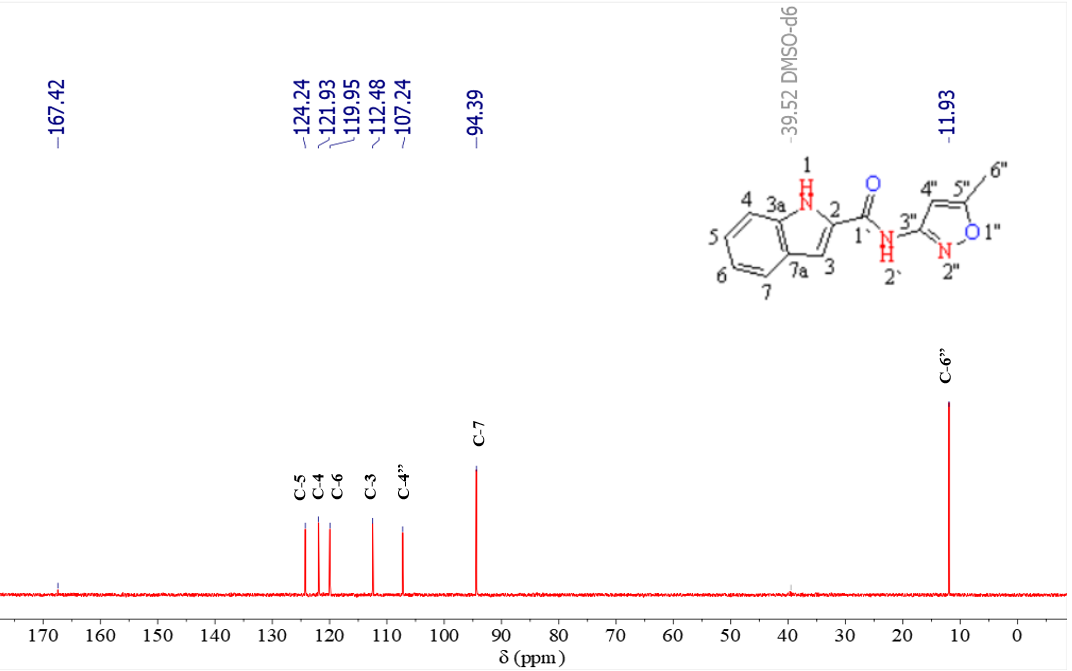
**

**The** **DEPT** **^13^C-NMR spectrum of compound 26.**

**Supplementary data charts (S# 19)**

**compounds (27)**

**
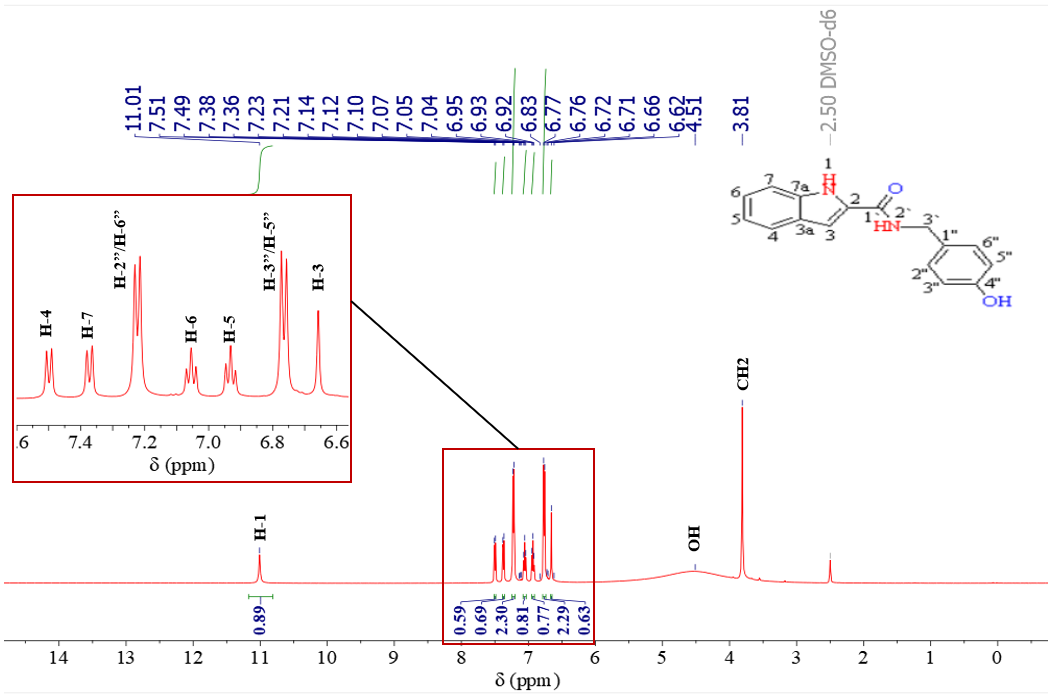
**

**The ^1^H-NMR spectrum of compound 27.**

**
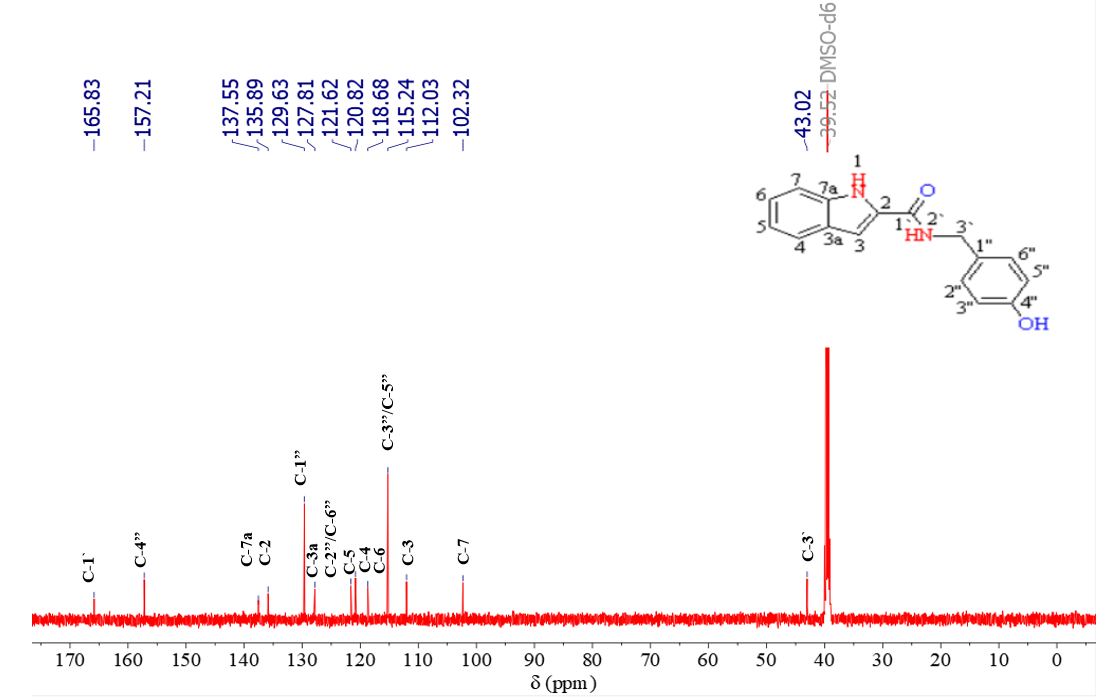
**

**The** **^13^C-NMR spectrum of compound 27.**

**
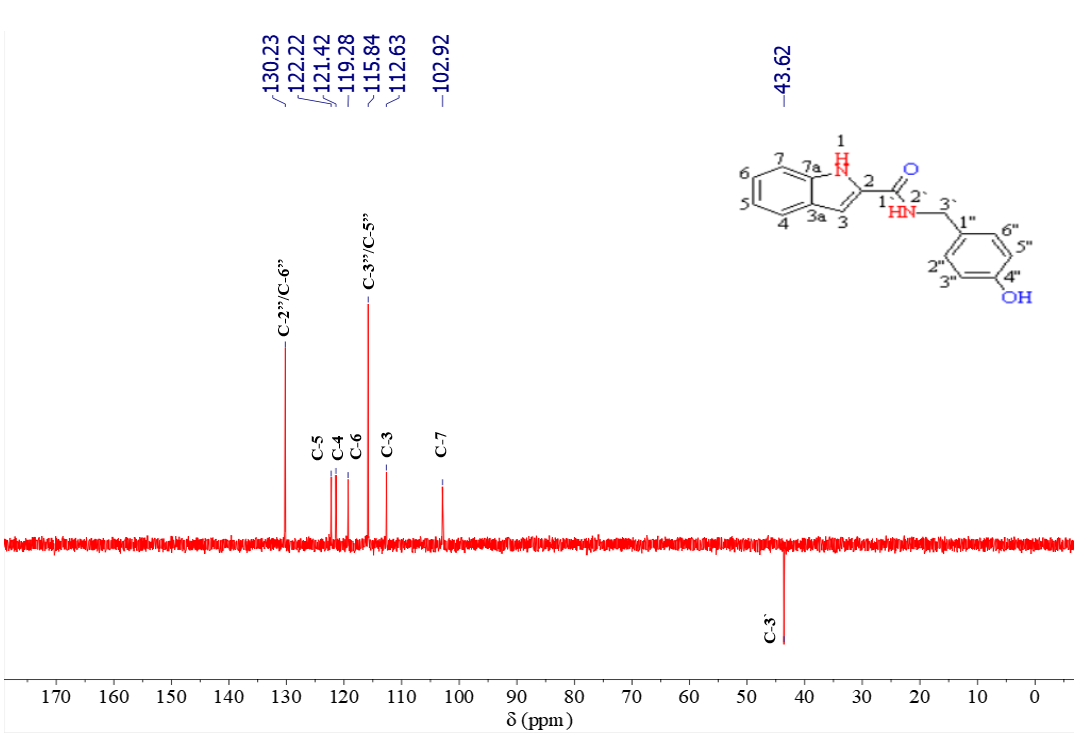
**

**The** **DEPT** **^13^C-NMR spectrum of compound 27.**

**Supplementary data charts (S# 20)**

**compounds (28)**


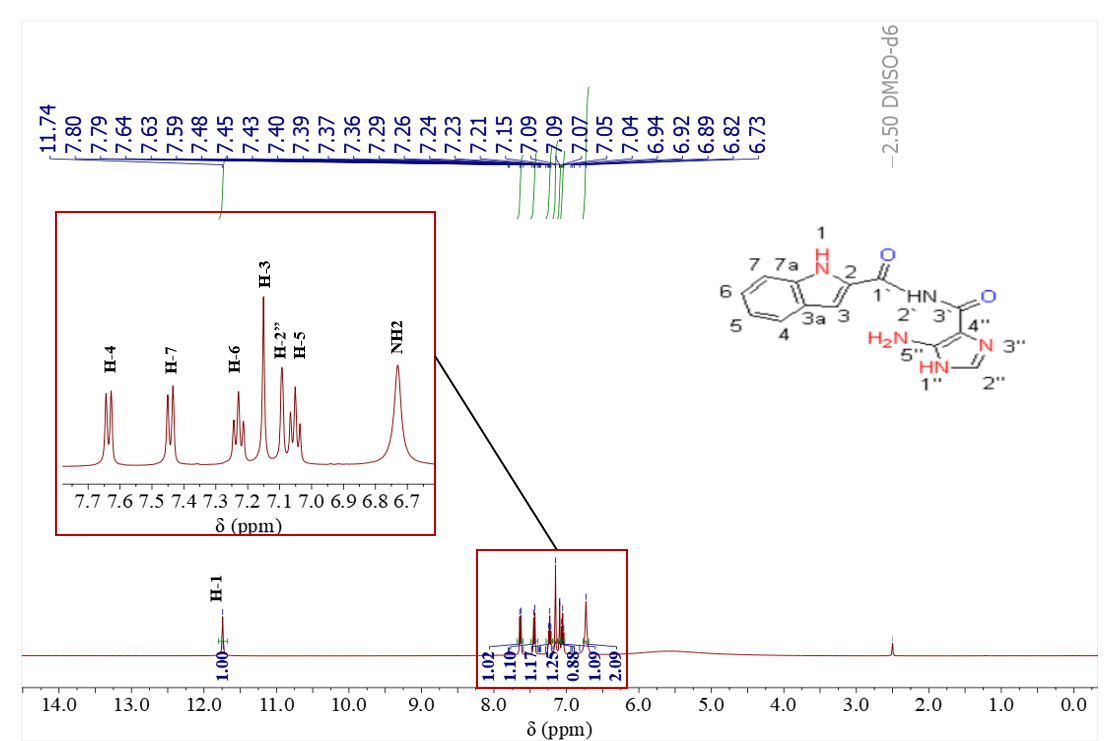


**The ^1^H-NMR spectrum of compound 28.**


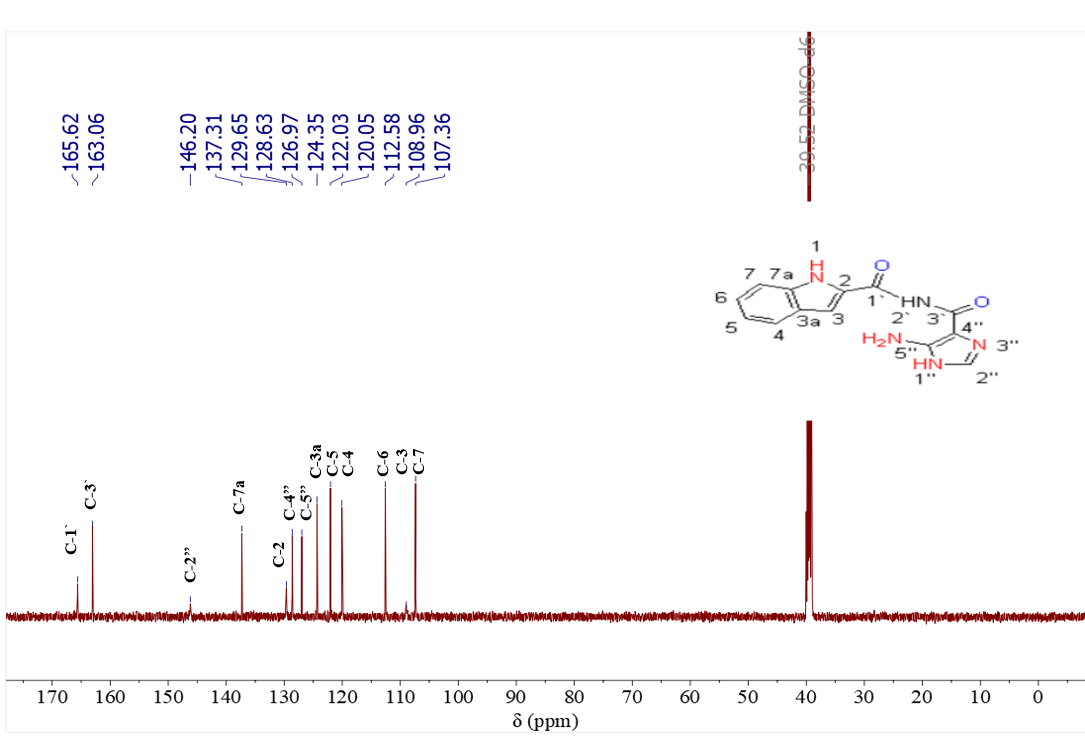


**The** **^13^C-NMR spectrum of compound 28.**

**Supplementary data charts (S# 21)**

**compounds (29)**

**
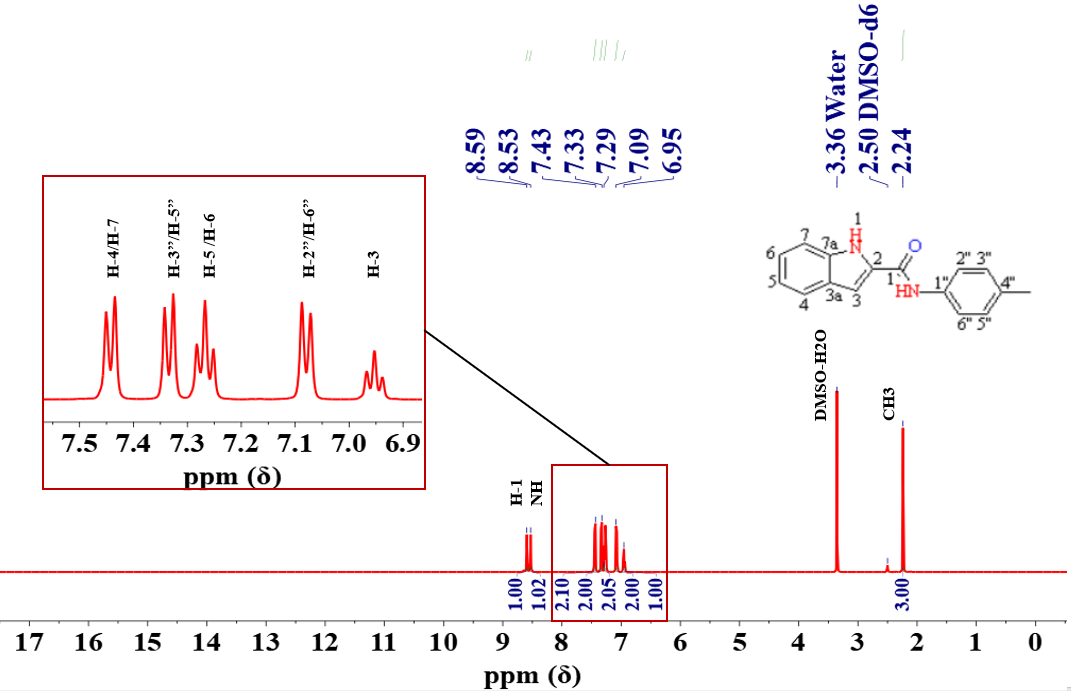
**

**The ^1^H-NMR spectrum of compound 29.**

**
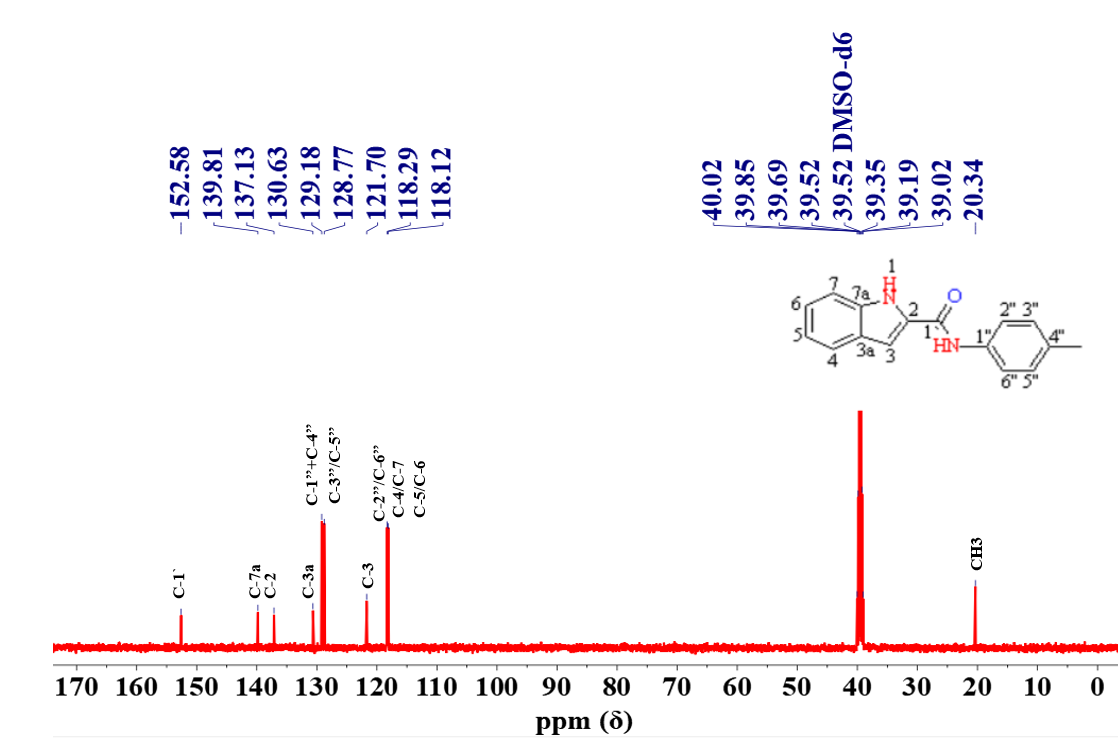
**

**The** **^13^C-NMR spectrum of compound 29.**

**
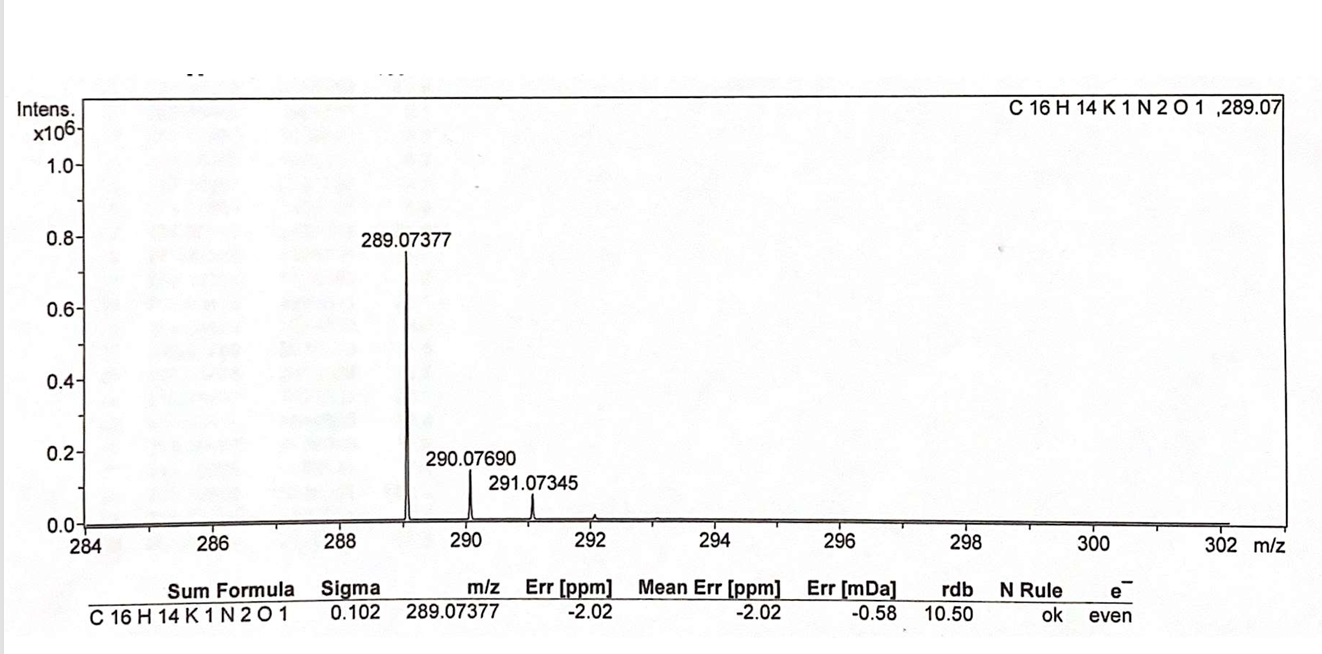
**

**The** **HRMS (ESI) spectrum of compound 29.**
