# Supplementary material for: Integrative computational, synthetic, experimental evaluation of targeted inhibitors against matrix metalloproteinase-9: Toward precision modulation of proteolytic activity
Source: PLoS One. 2026 Feb 17;21(2):e0337544. doi: 10.1371/journal.pone.0337544 (PMC12912705; doi:10.1371/journal.pone.0337544)
Supplement: S1 Table — (DOCX) [file pone.0337544.s002.docx]

**Table S1**. Reported selective MMP-9 inhibitors with their IC_50_ values.

| **No.** | **Inhibitor** | **MMP-9 IC_50_** | **Chemical Structure** | **References** |
| --- | --- | --- | --- | --- |
|  | **Compound i** | 0.2 nm |  | [1] |
|  | **Compound ii** | 0.2 nm |  | [2] |
|  | **Compound iii** | 0.01 nm |  | [3] |
|  | **Compound iv** | 0.9 nm |  | [4] |
|  | **Compound v** | 0.5 nm |  | [5] |
|  | **Compound vi** | 1.8 nm |  | [6] |
|  | **Compound vii** | 0.5 nm |  | [7] |
|  | **Compound viii** | 0.4 nm |  | [8] |
|  | **Compound ix** | 3.6 nm |  | [9] |
|  | **Compound x** | 3 nm |  | [10] |
|  | **Compound xi** | 492 nm |  | [11] |
|  | **Compound xii** | 4.5 nm |  | [12] |
|  | **Compound xiii** | 0.09 nm |  | [13] |
|  | **Compound xiv** | 1 nm |  | [14] |
|  | **Compound xv** | 13 nm |  | [15] |
|  | **Compound xvi** | 0.9 nm |  | [16] |
|  | **Compound xvii** | 3 nm |  | [17] |
|  | **Compound xviii** | 7.4 nm |  | [18] |
|  | **Compound xix** | 5.5 nm |  | [18] |
|  | **Compound xx** | 13.4 µm |  | [2] |
|  | **Compound xxi** | 26.9 µm |  | [19] |
|  | **Compound xxii** | 0.9 nm |  | [3] |
|  | **Compound xxiii** | 0.7 nm |  | [3] |
|  | **Compound xxiv** | 26 nm |  | [4] |
|  | **Compound xxv** | 26 nm |  | [20] |
|  | **Compound xxvi** | 17 nm |  | [20] |
|  | **Compound xxvii** | 0.6 nm |  | [5] |
|  | **Compound xxviii** | 9.10 nm |  | [6] |
|  | **Compound xxix** | 2.5 nm |  | [7] |
|  | **Compound xxx** | 1.3 nm |  | [7] |
|  | **Compound xxxi** | 13 µm |  | [8] |
|  | **Compound xxxii** | 52 nm |  | [9] |
|  | **Compound xxxiii** | 300 nm |  | [10] |
|  | **Compound xxxiv** | 4 nm |  | [21] |
|  | **Compound xxxv** | 120 µm |  | [22] |
|  | **Compound xxxvi** | 18000 nm |  | [22] |
|  | **Compound xxxvii** | 480 nm |  | [23] |
|  | **Compound xxxviii** | 270 nm |  | [24] |
|  | **Compound xxxix** | 60 nm |  | [11] |
|  | **Compound xl** | 2 nm |  | [25] |
|  | **Compound xli** | 100 nm |  | [26] |
|  | **Compound xlii** | 201 nm |  | [27] |
|  | **Compound xliii** | 1 nm |  | [27] |
