# Supplementary material for: Integrative computational, synthetic, experimental evaluation of targeted inhibitors against matrix metalloproteinase-9: Toward precision modulation of proteolytic activity
Source: PLoS One. 2026 Feb 17;21(2):e0337544. doi: 10.1371/journal.pone.0337544 (PMC12912705; doi:10.1371/journal.pone.0337544)
Supplement: S2 Table — (DOCX) [file pone.0337544.s003.docx]

**Table S2.** The Glide docking score (Kcal/mol) and interaction forces of the full match hits-based on virtual screening.

| **No.** | **# NSC Number** | **Glide Score** | **Interaction Forces** | **Chemical Class** | **Chemical structure** |
| --- | --- | --- | --- | --- | --- |
|  | **339919** | -10.88 | -Three H-Bonds with ASP235, and ALA191  -Two π-π stacking with PHE110 | Indole scaffold |  |
|  | **172777** | -10.28 | - Three H-Bonds with GLY233, ALA191, and ARG106  -One π-π stacking with PHE110 | Indole scaffold |  |
|  | **524593** | -9.40 | -Three H-Bonds with GLY233, ALA191, and GLY105  -Two π-π stacking with PHE110 | Indole scaffold |  |
|  | **166619** | -9.26 | -Three H-Bonds with ARG106, ALA191, and LEU234  -Three π-π stacking with TYR179 and PHE110 | Purine scaffold |  |
|  | **14076** | -9.76 | -One H-Bonds with ASP103  -One salt bridge with ASP235  -One π-π stacking with PHE110 | Acridine scaffold |  |
|  | **169127** | -9.18 | -Four H-Bonds with GLY105, ARG106, and GLY233  -One π-π stacking with HIS190 | Indole scaffold |  |
|  | **120956** | -9.09 | -Four H-Bonds with GLY105, ASP103, and ALA191  -Two π-π stacking with PHE110 and TYR179 | Purine scaffold |  |
|  | **122331** | -8.98 | -Two H-Bonds with ALA191, and ARG106  -Two π-π stacking with PHE110 | Benza-imidazole scaffold |  |
|  | **665810** | -8.95 | -Three H-Bonds with ASP235, and GLY233 | Butyric acid methyl ester |  |
|  | **524594** | -9.01 | -Three H-Bonds with ALA191, ARG106, and GLY233  -Two π-π stacking with PHE110 | Indole scaffold |  |
|  | **630321** | -9.06 | - Three H-Bonds with GLY105, ASP235, and ARG106  -Two π-π stacking with PHE110 and TYR179 | Urea scaffold |  |
|  | **302326** | -8.24 | -Three H-Bonds with ALA191, PRO102, and ASP103  -One π-π stacking with HIS230 | Sulphone-amide scaffold |  |
|  | **630295** | -8.86 | -One H-Bond with ALA191  -One π-π stacking with TYR179 | 2,5-dioxo-cyclohexyl |  |
|  | **9336** | -8.42 | - Three H-Bonds with ALA191, ARG106, and GLY105  -One π-π stacking with PHE110 | Acridine scaffold |  |
|  | **649993** | -8.96 | -One H-Bond with ARG106 | Bicyclo oct-3-ene-2,8-dione scaffold |  |
|  | **353299** | -8.83 | -Two H-Bonds with ALA191, and GLY233  -One π-π stacking with HIS230 | Pyrimidine scaffold |  |
|  | **12270** | -8.15 | -Three H-Bonds with ALA191, TYR179, and ARG106  -One pi-cation bond with TYR179  -One salt bridge with ASP235  -One π-π stacking HIS230 | Acridine scaffold |  |
|  | **278006** | -7.91 | -Two H-Bonds with ARG106  -One π-π stacking with PHE110 | Indole scaffold |  |
|  | **72138** | -7.87 | -Three π-π stacking with TYR179 and PHE110 | Indole scaffold |  |
|  | **106658** | -7.73 | -One H-Bond with ALA191  -Two π-π stacking with PHE110 and TYR179 | Pyrimidine scaffold |  |
|  | **682362** | -7.79 | -Two H-Bonds with ALA191, and ASP103  -One pi-cation bond with HIS230  -One salt bridge with ASP235  -One π-π stacking with PHE110 | Acridine scaffold |  |
|  | **647382** | -7.71 | -One H-Bond with ARG106  -Three π-π stacking with PHE110 and TYR179 | Indole  Scaffold |  |
|  | **351546** | -7.39 | -Two H-Bonds with ARG106, and GLY233 | Pyrimidine scaffold |  |
|  | **72122** | -7.80 | -One H-Bond with ARG106  -Three π-π stacking with PHE110 and TYR179 | Indole  Scaffold |  |
|  | **278006** | -8.11 | -Two H-Bonds with ARG106  -One π-π stacking with TYR179 | Indole scaffold |  |
|  | **346230** | -7.16 | -One π-π stacking with HIS230 | Sulphone amide |  |
|  | **265396** | -7.15 | -One H-Bond with ARG106  -Two π-π stacking with PHE110 and TYR179 | Purine scaffold |  |
|  | **621394** | -7.13 | -Two H-Bonds with ALA191, and ARG106 | Piperidine-2,6-dione scaffold |  |
|  | **671876** | -6.17 | -One H-Bond with ARG106  -Two π-π stacking with PHE110 and TYR179 | Triazolo-thiazole scaffold |  |
|  | **351543** | -5.97 | -Two H-Bonds with ARG106 and GLY233 | Pyrimidine scaffold |  |
|  | **279252** | -5.95 | -One H-Bond with GLY233  -One π-π stacking with PHE110 | Pyrimidine scaffold |  |
|  | **351545** | -5.94 | -Two H-Bonds with ARG106 and GLY233 | Pyrimidine scaffold |  |
|  | **48157** | -5.76 | -Two H-Bonds with ARG106  -One π-π stacking with PHE110 | Pyrrole scaffold |  |
|  | **116058** | -6.61 | -Two π-π stacking with PHE110 and TYR179 | Indole scaffold |  |
|  | **509305** | -6.72 | - Four H-Bonds with ASP103, ASP235, ALA191, and ARG106  -One pi-cation bond with HIS230  -One salt bridge with ASP235  -Two π-π stacking with PHE110 and TYR179 | 2-imino methyl-phenol scaffold |  |
|  | **29859** | -6.36 | -One H-Bond with ALA191  -Two π-π stacking with PHE110 and TYR179 | Indole scaffold |  |
|  | **76457** | -4.52 | -Three π-π stacking with PHE110 and PHE107  - One salt bridge with ASP235 | Indole  Scaffold |  |
