# Supplementary material for: Integrative computational, synthetic, experimental evaluation of targeted inhibitors against matrix metalloproteinase-9: Toward precision modulation of proteolytic activity
Source: PLoS One. 2026 Feb 17;21(2):e0337544. doi: 10.1371/journal.pone.0337544 (PMC12912705; doi:10.1371/journal.pone.0337544)
Supplement: S3 Table — (DOCX) [file pone.0337544.s004.docx]

**Table S3.**  The Glide docking score (kcal/mol) and interaction forces of the partial match hits-based on virtual screening.

| **No.** | **# NSC Number** | **Glide Score** | **Interaction Forces** | **Chemical Class** | **Chemical Structure** |
| --- | --- | --- | --- | --- | --- |
|  | **372327** | -11.97 | -Five H-Bonds with GLY105, ASP103, HIS230, and ASP235.  -One π-π stacking with HIS190 | Indole  Scaffold |  |
|  | **94506** | -11.94 | -Four H-Bonds with ALA191, GLY105, and ASP235.  -Two π-π stacking with TYR179 and PHE110  -One halogen bond with GLN77 | Piperazine Scaffold |  |
|  | **617942** | -11.72 | -Three H-Bonds with ALA191, ARG106, and ASP235  -Two π-π stacking with TYR179 and PHE110 | Benz-Imidazole  Scaffold |  |
|  | **673418** | -11.50 | -Six H-Bonds with GLN77, HIS236, ASP235, ASP103, and PRO102.  -Two π-π stacking with TYR179 and PHE110 | Quinoline Scaffold |  |
|  | **634823** | -11.38 | -Nine H-Bonds with ASP103, ASP235, GLY105, GLY233, ASP113, GLY263, and ALA191.  -One π-π stacking with HIS230. | 3-Hydroxy-Propionic Acid Scaffold |  |
|  | **403468** | -11.37 | -Two H-Bonds with ASP235 and ALA191  -Two π-π stacking with TYR179 and PHE110 | N-Phenyl-Hydrazide Scaffold |  |
|  | **86390** | -11.30 | -Three H-Bonds with GLY233, ASP235, and ALA191  -Three π-π stacking with TYR179, HIS230 and PHE110 | Pyrazole  Scaffold |  |
|  | **13040** | -11.16 | -Three H-Bonds with ASP103, ASP235, and ALA191.  -Two π-π stacking with TYR179 and PHE110 | Quenoline Scaffold |  |
|  | **617941** | -11.13 | -Three H-Bonds with ASP235, ARG106, and ALA191.  -Two π-π stacking with TYR179 and PHE110 | Benz-Imidazole  Scaffold |  |
|  | **620023** | -11.09 | -Four H-Bonds with GLY105, ARG106, ASP235, and ALA191.  -Two π-π stacking with TYR179 and PHE110 | Benz-Imidazole  Scaffold |  |
|  | **86172** | -10.95 | -Three H-Bonds with ARG106, and ALA191.  -Three π-π stacking with TYR179 and PHE110 | Benz-Imidazole  Scaffold |  |
|  | **617945** | -10.80 | -Three H-Bonds with ARG106, ASP235, and ALA191.  -Two π-π stacking with TYR179 and PHE110 | Benz-Imidazole  Scaffold |  |
|  | **108594** | -10.735 | -Three H-Bonds with PRO102, GLY105, and ALA191.  -Four π-π stacking with TYR179, HIS190, HIS230, and PHE110. | Purine  Scaffold |  |
|  | **74679** | -10.713 | -Two H-Bonds with GLY233 and ALA191  -Two π-π stacking with TYR179 and PHE110 | Bis-Phenylamide Scaffold |  |
|  | **617946** | -10.66 | -Three H-Bonds with ASP235, ARG106, and ALA191.  -One π-π stacking with PHE110 | Benz-Imidazole  Scaffold |  |
|  | **404808** | -10.64 | -Two H-Bonds with GLY233 and ALA191  -One π-π stacking with TYR179 | N1, N10-Diphenyldecanediamide |  |
|  | **327925** | -10.55 | -Two H-Bonds with GLY233 and ALA191  -Two π-π stacking with TYR179 and PHE110 | Purine  Scaffold |  |
|  | **14071** | -10.54 | -Three H-Bonds with GLY105, ASP103, and ARG106.  -One π-π stacking with PHE110 | Acridine  Scaffold |  |
|  | **143509** | -10.47 | -Two H-Bonds with ASP103, and ARG106.  -Two π-π stacking with PHE110 and TYR179. | Quinoline  Scaffold |  |
|  | **10619** | -10.44 | -Three H-Bonds with ASP235, PRO102, and ARG106.  -Two π-π stacking with PHE110 and TYR179. | 3-[(2-Hydroxy-Ethylamino)-Methyl]-Biphenyl-2-Ol |  |
|  | **138483** | -10.41 | -Four H-Bonds with ASP103, PRO102, and ALA191.  -One π-π stacking with TYR179. | Triazine  Scaffold |  |
|  | **172777** | -10.28 | -Three H-Bonds with GLY233, ARG106, and ALA191.  -One π-π stacking with PHE110 | Indole  Scaffold |  |
|  | **80445** | -10.27 | -Three H-Bonds with GLY233, ARG106, and ALA191  -One halogen bond with GLN108  -One π-π stacking with TYR179 | Quinazoline Scaffold |  |
|  | **684438** | -10.26 | -Five H-Bonds with ASP 259, GLY233, ASP103, HIS236, and HIS236. | Anthracene Scaffold |  |
|  | **8660** | -10.23 | -Two H-Bonds with ARG106 and ALA191.  -Three π-π stacking with PHE110, HIS230, and TYR179. | Naphthalene Scaffold |  |
|  | **254165** | -10.18 | -Six H-Bonds with ALA191, GLY233, ASP235, PRO102, and GLY105.  - Two π-π stacking with PHE110. | Indole Scaffold |  |
|  | **138474** | -10.16 | -Four H-Bonds with GLY233 and ALA191  -Two π-π stacking with PHE110, and TYR179. | Bis(3-Phenylthiourea) Scaffold |  |
|  | **684442** | -10.16 | -Seven H-Bonds with GLY233, ASP235, GLY105, and ASP103.  -One π-π stacking with PHE110. | Anthracene Scaffold |  |
|  | **14076** | -10.14 | -Three H-Bonds with ARG106, ASP103, and ALA191  -One pi-cation bond with HIS230  -One π-π stacking with PHE110. | Acridine  Scaffold |  |
|  | **280702** | -10.12 | -Two H-Bonds with ASP235 and ALA191  - Three π-π stacking with PHE110, and TYR179. | Indole Scaffold |  |
|  | **525457** | -10.10 | -Two H-Bonds with ASP235 and ALA191  -Two π-π stacking with PHE110 | Indole Scaffold |  |
|  | **51663** | -10.08 | -Four H-Bonds with ASP235, ASP103, ARG106, and ALA191  -One π-π stacking with HIS190 | Di-Mercapto Octane-Diyl) Bis Diphenol Scaffold |  |
|  | **610501** | -10.08 | -Two H-Bonds with ARG106, and ALA191  -Three π-π stacking with HIS230, TYR179, and PHE110 | Pyrazole Scaffold |  |
|  | **402196** | -10.06 | -Four H-Bonds with ASP235, ASP103, ARG106, and LEU234  -One pi-cation bond with HIS236 | Bis (Benzyl Amino) 1,4-Diol Scaffold |  |
|  | **8648** | -10.02 | -Two H-Bonds with ARG106 and ALA191  -Three π-π stacking with TYR179 and PHE110 | Bi-Indole]-3,3'-Diol  Scaffold |  |
|  | **128718** | -9.94 | -Two H-Bonds with ASP103 and ARG106  -Three π-π stacking with HIS230, TYR179, and PHE110 | Quinoline Scaffold |  |
|  | **364279** | -9.90 | -Four H-Bonds with ARG106, GLY233, and ALA191  -One π-π stacking with PHE110 | Indole Scaffold |  |
|  | **327926** | -9.88 | -Three H-Bonds with GLY105, HIS230, and ALA191  -One π-π stacking with PHE110 | Purine Scaffold |  |
|  | **656628** | -9.86 | -Two H-Bonds with GLY233 and ALA191  -Two π-π stacking with HIS230 and TYR179 | Quinoxaline Scaffold |  |
|  | **673414** | -9.84 | -Two H-Bonds with ASP103 and ASP235  -Two π-π stacking with PHE110 and TYR179 | Quinoline Scaffold |  |
|  | **11963** | -9.84 | -Three H-Bonds with ARG106 and ASP235  -Two π-π stacking with HIS230 and TYR179 | Bis (Benzyl-4-Chlorophenol) Scaffold |  |
|  | **617940** | -9.83 | -Two H-Bonds with ASP103  -One π-π stacking with PHE110 | Quinoline Scaffold |  |
|  | **34322** | -9.81 | -Three H-Bonds with ASP235, GLY105, and ARG106  -One π-π stacking with PHE110 | Chlorophenyl Aminoethanol Scaffold |  |
|  | **352272** | -9.79 | -Two H-Bonds with GLY233 and ALA191  -One π-π stacking with HIS230 | Pyrimidine Scaffold |  |
|  | **617947** | -9.79 | -One H-Bond with ALA191  -Two π-π stacking with TYR179 and PHE110 | Benz-Imidazole Scaffold |  |
|  | **673415** | -9.77 | -Three H-Bonds with ASP103, ASP235, and GLY105  - Two π-π stacking with TYR179 and PHE110 | Quinoline Scaffold |  |
|  | **109453** | -9.76 | -One H-Bond with ARG106  -Two π-π stacking with HIS230 and PHE110 | Quinoline Scaffold |  |
|  | **353681** | -9.75 | -One H-Bond with ALA191  -Two π-π stacking with TYR179 and PHE110 | Aminothiazolyl Benzene-1,2-Diol Scaffold |  |
|  | **201330** | -9.74 | -Two H-Bonds with GLY233 and ALA191  -Three π-π stacking with TYR179 and PHE110 | Indole  Scaffold |  |
|  | **656627** | -9.74 | -Two H-Bonds with GLY233 and ALA191 | Quinoxaline Scaffold |  |
|  | **97980** | -9.74 | -Three H-Bonds with GLY233 and ALA191  -One π-π stacking with TYR179 | Pyrimidine Scaffold |  |
|  | **135794** | -9.74 | -Two H-Bonds with GLY105 and ALA191  -One pi-cation bond with HIS230  -One π-π stacking with TYR179 | Quinoline Scaffold |  |
|  | **125868** | -9.73 | -One H-Bond with ALA191  -Three π-π stacking with TYR179 and PHE110 | Indole Scaffold |  |
|  | **3288** | -9.72 | -Four H-Bonds with PRO102, ASP235, ARG106, and ALA191  -Three π-π stacking with TYR179 and PHE110 | Benz-Imidazole Scaffold |  |
|  | **281311** | -9.71 | -Three H-Bonds with ARG106, GLY233, and ALA191  -One π-π stacking with TYR179 | Purine Scaffold |  |
|  | **365435** | -9.69 | -One H-Bond with ALA191  -Two π-π stacking with TYR179 and PHE110 | Piperidine  Di-Phenyl-Amine |  |
|  | **623292** | -9.67 | -Four H-Bonds with PRO102, ASP235, and GLY105  -Two π-π stacking with TYR179 and PHE110 | Pyrimidine Scaffold |  |
|  | **201331** | -9.67 | -Two H-Bonds with GLY233 and ALA191  -Three π-π stacking with TYR179 and PHE110 | Indole Scaffold |  |
|  | **371844** | -9.67 | - One H-Bond with ALA191  -Two π-π stacking with PHE110 and HIS230 | Benz-Imidazole Scaffold |  |
|  | **77506** | -9.66 | -Two H-Bonds with ARG106 and ALA191  -One π-π stacking with HIS230 | Benzo-Thiazoline Scaffold |  |
|  | **136326** | -9.66 | -One H-Bond with ALA191  -One π-π stacking with PHE110 | Di-Phenyl-Amine Scaffold |  |
|  | **337772** | -9.65 | -Two H-Bonds with GLY233 and ALA191  -Two π-π stacking with TYR179 and PHE110 | Purine Scaffold |  |
|  | **205792** | -9.61 | -Two H-Bonds with GLY233 and ALA191  -Three π-π stacking with TYR179, PHE110 and HIS230 | Di-Phenyl-Amine Scaffold |  |
|  | **237033** | -9.60 | -One H-Bond with ALA191  -Three π-π stacking with TYR179 and PHE110 | Indole Scaffold |  |
|  | **205834** | -9.59 | -Three H-Bonds with GLY233, ASP235, and ALA191  -Twp π-π stacking with TYR179 and PHE110 | Di-Phenyl-Amine Scaffold |  |
|  | **674087** | -9.57 | -Two H-Bonds with ASP235 and ASP103  -Two π-π stacking with TYR179, and PHE110 | Quinoline Scaffold |  |
|  | **663889** | -9.54 | -Three H-Bonds with GLY 100 and ALA191 | Pyrimidine Scaffold |  |
|  | **219865** | -9.51 | -One H-Bond with GLY233  -One π-π stacking with TYR179 | Benzyloxy Phenyl Scaffold |  |
|  | **337773** | -9.48 | -Two H-Bonds with GLY233, and ALA191  -One π-π stacking with PHE110 | Purine Scaffold |  |
|  | **358534** | -9.41 | -Four H-Bonds with HIS230, ARG106, and ALA191  - Two π-π stacking with PHE110 and TYR179 | Purine Scaffold |  |
|  | **123344** | -9.33 | -Three H-Bonds with LEU234, ARG106, and ALA191  - One π-π stacking with PHE110 | Quinoxaline Scaffold |  |
|  | **216170** | -9.27 | -Two H-Bonds with GLY233, and ALA191  -Two π-π stacking with PHE110 and TYR179 | Di-Phenyl-Amine Scaffold |  |
|  | **34320** | -9.26 | -Two H-Bonds with ARG106, and PRO102  - Four π-π stacking with HIS190, PHE110, and TYR179 | Benzo -Dioxole Scaffold |  |
|  | **17591** | -9.23 | -One H-Bond with ALA191  -Two π-π stacking with TYR179 and PHE110 | Benzophenoxazin-8-Amine Scaffold |  |
|  | **31701** | -9.19 | -Three H-Bonds with PRO102, ASP235, and ARG106  -One π-π stacking with PHE110 | (4-Methoxyphenyl)-2-Phenylethyl)Ethane-1,2-Diamine |  |
|  | **14072** | -9.14 | -Two H-Bonds with ASP235, and GLY105  -One π-π stacking with PHE110 | Acridine Scaffold |  |
|  | **665306** | -9.05 | -One H-Bond with ALA191  -Two π-π stacking with PHE110 | Indole Scaffold |  |
|  | **614992** | -9.01 | -Four H-Bonds with ARG106, and ALA191  -Three π-π stacking with PHE110 and TYR179 | Pyrimidine Scaffold |  |
|  | **47626** | -8.93 | -Two H-Bonds with HIS230, and ALA191  -One π-π stacking with PHE110 | Pyrimidine Scaffold |  |
|  | **685824** | -8.84 | -Three H-Bonds with ARG106 and ALA191  -Three π-π stacking with HIS230, PHE110, and TYR179 | Purine Scaffold |  |
|  | **215562** | -8.76 | -Two H-Bonds with GLY233, and ALA191  -Three π-π stacking with HIS230, PHE110, and TYR179 | Di-Phenyl-Amine  Scaffold |  |
|  | **639945** | -8.68 | -Two H-Bonds with ASP235, and ALA191  -Two π-π stacking with PHE110, and TYR179 | Benzo-Thiazin Scaffold |  |
|  | **146503** | -8.60 | -Two H-Bonds with ASP235, and GLY233  -Three π-π stacking with TYR179, and PHE110 | Carbazole Scaffold |  |
|  | **631566** | -8.53 | -One H-Bond with ALA191  -Two π-π stacking with TYR179, and PHE110 | M-Tolylamino-Dihydrothiazolyl Acetamide Scaffold |  |
|  | **404890** | -8.49 | - Two H-Bonds with ARG106, and ALA191  -Two π-π stacking with TYR179, and PHE110 | Benzo-Diazaborole Scaffold |  |
|  | **664711** | -8.47 | -Four H-Bonds with PRO102, ASP235, GLY233, and ARG106 | Indole Scaffold |  |
|  | **336000** | -8.45 | -One H-Bond with ALA191  -Two π-π stacking with PHE110  -Two salt bridges with ARG106, and ASP235 | Carbazole Scaffold |  |
|  | **123019** | -8.44 | -One H-Bond with ALA191  -One halogen bond with GLN108 | Bis(N-(2-Chlorobenzyl)Methanamine) Scaffold |  |
|  | **97964** | -8.40 | -Two H-Bonds with ALA191  -One π-π stacking with TYR179 | Pyrimidine Scaffold |  |
|  | **56908** | -8.38 | -Three H-Bonds with ASP235, and ALA191  -Two π-π stacking with PHE110 | Benz-Imidazole Scaffold |  |
|  | **622807** | -8.35 | -Two H-Bonds with ARG106  -Three π-π stacking with HIS230, and PHE110 | Indole Scaffold |  |
|  | **404892** | -8.34 | -Two H-Bonds with ARG106  -Three H-Bonds with HIS230, and PHE110 | Benzodiazaborole Scaffold |  |
|  | **98576** | -8.28 | -One H-Bond with ALA191  -Two π-π stacking with TYR179, and PHE110 | Pyridine Scaffold |  |
|  | **98567** | -8.22 | -One H-Bond with ALA191  -Two π-π stacking with TYR179, and PHE110 | Pyridine Scaffold |  |
|  | **201714** | -8.19 | -Two H-Bonds with ARG106  - One π-π stacking with PHE110 | Thiourea Scaffold |  |
|  | **142115** | -8.19 | -Two H-Bonds with ALA191  - One π-π stacking with TYR179 | Benz-Imidazole Scaffold |  |
|  | **682528** | -8.18 | -Three H-Bonds with ALA191, and ARG106  - Two π-π stacking with TYR179, and PHE110 | Purine Scaffold |  |
|  | **404798** | -8.17 | -One H-Bond with ALA191  - Two π-π stacking with TYR179, and PHE110 | 2-Phenyl Hydrazine Scaffold |  |
|  | **290675** | -8.15 | -Two H-Bonds with ALA191, and ARG106 | Tetra-Azine Scaffold |  |
|  | **321162** | -8.14 | -One H-Bond with ALA191  -Two π-π stacking with PHE110 | Benz-Imidazole Scaffold |  |
|  | **380509** | -8.13 | -One H-Bond with ALA191  -Two π-π stacking with PHE110 | Benz-Imidazole Scaffold |  |
|  | **152447** | -8.11 | -One H-Bond with PRO102  -Two π-π stacking with PHE110, and TYR179 | Pyridine Scaffold |  |
|  | **72116** | -8.08 | -One H-Bond with ARG106  -Three π-π stacking with PHE110, and TYR179 | Indole Scaffold |  |
|  | **225227** | -8.02 | -Two H-Bonds with ARG106, and GLY233  -Three π-π stacking with PHE110, and HIS230 | Quinoline Scaffold |  |
|  | **23672** | -7.98 | -Two H-Bonds with ALA191  -Three π-π stacking with PHE110, TYR179, and HIS230 | Pyrimidine Scaffold |  |
|  | **106506** | -7.97 | -One H-Bond with ALA191  -Two π-π stacking with PHE110 | Indole Scaffold |  |
|  | **290758** | -7.96 | -One H-Bond with ALA191  -One π-π stacking with PHE110 | Di Thio-Carbamate Scaffold |  |
|  | **689079** | -7.95 | -One H-Bond with ARG106  - Three π-π stacking with PHE110, and TYR179 | Indole Scaffold |  |
|  | **663988** | -7.89 | -Two H-Bonds with ALA191, and ARG106  - One π-π stacking with TYR179 | 2-Phenyl Hydrazinylidene |  |
|  | **211785** | -7.88 | -Two H-Bonds with ALA191, and ARG106  -Three π-π stacking with PHE110, and TYR179 | Purine Scaffold |  |
|  | **211040** | -7.87 | -Two H-Bonds with ALA191, and ARG106  -Three π-π stacking with PHE110, and TYR179 | Purine Scaffold |  |
|  | **27310** | -7.83 | -Two H-Bonds with ALA191, and GLY233  -Three π-π stacking with PHE110, and TYR179 | Purine Scaffold |  |
|  | **14125** | -7.82 | -One H-Bond with ALA191  -Two π-π stacking with PHE110, and TYR179 | Purine Scaffold |  |
|  | **35971** | -7.80 | - Two H-Bonds with ALA191, and HIS230  -Two π-π stacking with HIS230, and TYR179 | Indole Scaffold |  |
|  | **685838** | -7.76 | -One H-Bond with HIS230  -Two π-π stacking with HIS230, and TYR179 | Purine Scaffold |  |
|  | **3276** | -7.63 | -One H-Bond with ARG106  - Two π-π stacking with PHE110, and TYR179 | Indole Scaffold |  |
|  | **647382** | -7.61 | -One H-Bond with ARG106  - Three π-π stacking with PHE110, and TYR179 | Indole Scaffold |  |
|  | **283169** | -7.58 | -Two H-Bonds with ASP235, and ALA191  -One pi-cation bond with HIS236 | Indole Scaffold |  |
|  | **191423** | -7.57 | -Two H-Bonds with ARG106  - Two π-π stacking with PHE110, and TYR179 | Thiourea Scaffolds |  |
|  | **353884** | -7.51 | -Two H-Bonds with ALA191, and GLY233  -Three π-π stacking with PHE110, and TYR179 | Benz-Imidazole Scaffold |  |
|  | **282736** | -7.49 | -Two H-Bonds with ALA191, and ARG106  -Two π-π stacking with PHE110 | Purine Scaffold |  |
|  | **210554** | -7.48 | -Two H-Bonds with ALA191, and ARG106  -Three π-π stacking with PHE110, and TYR179 | Purine Scaffold |  |
|  | **114078** | -7.36 | -One H-Bond with ALA191  -Two π-π stacking with PHE110, and TYR179 | Pyridine Scaffold |  |
|  | **605412** | -7.36 | -One H-Bond with ALA191  -Two π-π stacking with PHE110 | Indole Scaffold |  |
|  | **118210** | -7.29 | -Two H-Bonds with ALA191, and ARG106  -Three π-π stacking with PHE110, and TYR179 | Purine Scaffold |  |
|  | **109173** | -7.28 | -Two H-Bonds with ALA191, and ARG106  -Three π-π stacking with PHE110, and TYR179 | Purine Scaffold |  |
|  | **165209** | -7.16 | -One H-Bond with ARG106  -Two π-π stacking with PHE110, and TYR179 | Indole Scaffold |  |
|  | **12454** | -7.09 | -One H-Bond with ARG106  -Two π-π stacking with PHE110 | Acridine Scaffold |  |
|  | **327373** | -7.04 | -One H-Bond with ALA191  -One π-π stacking with HIS230 | Dithio-Carbamate Scaffold |  |
|  | **616950** | -7.03 | -One H-Bond with ALA191  -One π-π stacking with HIS230 | Quinazoline Scaffold |  |
|  | **128807** | -6.97 | -One H-Bond with ARG106  -Three π-π stacking with HIS230, and PHE110 | Indazole Scaffold |  |
|  | **656708** | -6.86 | -One H-Bond with ARG106 | Pyrimidine Scaffold |  |
|  | **92588** | -6.75 | -Three H-Bonds with GLY233, ALA191, and GLN108  -Two π-π stacking with PHE110 | Pyrimidine Scaffold |  |
|  | **150911** | -6.60 | -One H-Bond with ARG106 | Oxazine Scaffolds |  |
|  | **150789** | -5.64 | -One H-Bond with ARG106  -One π-π stacking with HIS230 | Indole Scaffold |  |
|  | **152461** | -5.05 | -Two H-Bonds with GLY233, and ARG106  - Two π-π stacking with TYR179, and PHE110 | Pyridine Scaffold |  |
|  | **693338** | -4.44 | -One H-Bond with GLY233  - One π-π stacking with PHE110 | Pyrazole Scaffold |  |
