# Supplementary material for: Integrative computational, synthetic, experimental evaluation of targeted inhibitors against matrix metalloproteinase-9: Toward precision modulation of proteolytic activity
Source: PLoS One. 2026 Feb 17;21(2):e0337544. doi: 10.1371/journal.pone.0337544 (PMC12912705; doi:10.1371/journal.pone.0337544)
Supplement: S4 Table — (DOCX) [file pone.0337544.s005.docx]

**Table S4.** The Glide docking score (kcal/mol) and interaction forces of reported inhibitors against WT MMP-9 (PDB ID: 1GKC) and MUT MMP-9 (PDB ID:2OW1).

|  | | **MUT MMP-9**  **(2OW1)** | | **WT MMP-9**  **(1GKC)** | |  | |
| --- | --- | --- | --- | --- | --- | --- | --- |
| **No.** | **Inhibitor** | **Glide docking score**  **(kcal/mol)** | **Interaction Forces** | **Glide docking score**  **(kcal/mol)** | **Interaction Forces** | **Chemical Structure** | **Reference** |
|  | **Z 6** | -12.66 | - Two π-cation with Zn^+2^  -two coordination bonds with Zn^+2^  -Two π-π stacking with HIS411 and HIS401  -five H-bonds with GLN402, PRO421, ALA189, LEU188 | -6.86 | -one π-π stacking with TYR423  -two H-bonds with PRO421 and LEU188 |  | [28] |
|  | **Z 170** | -11.08 | -One coordination bond with ZN^+2^  -one π-π stacking with HIS401  -four H-bonds with ACE109, TYR420, LEU397, ALA189 | -5.99 | -two coordination bonds with Zn^+2^ ion  -one π-π stacking with HIS401  -Three H-bonds with GLU 402 and GLY 186 |  | [25] |
|  | **Z 141** | -10.74 | -One coordination bond and one salt bridge with ZN^+2^  -one π-cation with ZN^+2^  -Two H-bonds with LEU188, and ALA189. | -11.63 | -Two coordination bonds with Zn^+2^ ion  -one salt bridge with Zn^+2^  -one π-π stacking with HIS401  -One H-bond with PRO421 |  | [21] |
|  | **Z 133** | -10.69 | - one π-cation with Zn^+2^  -two coordination bonds with Zn^+2^  -one π-π stacking with HIS411  -three H-bonds with GLN402, ALA189, LEU188 | -4.53 | -one coordination bond with Zn^+2^ ion  -Two H-bonds with PRO421 |  | [29] |
|  | **Z 10** | -10.51 | - one π-cation with Zn^+2^  -two coordination bonds with Zn^+2^  -two π-π stacking with PHE110, and HIS401  -Four H-bonds with GLN402, ALA189, LEU188 | -6.36 | -one coordination bonds with Zn^+2^ ion  -one π-cation bond with Zn^+2^  -five H-bonds with LEU188, ALA189, GLU 402, and TYR423 |  | [1] |
|  | **Z 143** | -10.26 | - one π-cation with ZN^+2^  -two coordination bonds with ZN^+2^  -two H-bonds with GLN402, and ALA189 | -6.43 | -Two coordination bonds with Zn^+2^ ion  -one π-π stacking with HIS401  -Two H-bonds with ALA189 and GLU 402 |  | [21] |
|  | **Z 147** | -10.06 | - one π-cation with Zn^+2^  -two coordination bonds with Zn^+2^  -four H-bonds with GLN402, ALA189, LEU188 | -5.51 | -two coordination bonds with Zn^+2^ ion  -one π-π stacking with HIS401  -Four H-bonds with LEU188, GLU 402, and TYR420 |  | [3] |
|  | **Z 35** | -9.89 | - one π-cation with Zn^+2^  -two coordination bonds and one salt bridge with Zn^+2^  -two H-bonds with ALA189, and LEU188 | -11.25 | -One coordination bond with Zn^+2^ ion  -one salt bridge with Zn^+2^  -One π-cation bond with Zn^+2^  -Two H-bonds with PRO421 and LEU188 |  | [30] |
|  | **Z 14** | -9.52 | -One coordination bonds with Zn^+2^  -one H-bond with ALA189 | -5.70 | -one π-π stacking with TYR423  -Two H-bonds with LEU188 and ALA189 |  | [31] |
|  | **Z 28** | -9.50 | - one π-cation with Zn^+2^  -two coordination bonds with Zn^+2^  -two π-π stacking with HIS411 and HIS401  -three H-bonds with GLN402, ALA189, LEU188 | -6.89 | -Two coordination bonds with Zn^+2^ ion  -one π-π stacking with TYR423  -Three H-bonds with LEU188 and GLU 402 |  | (1) |
|  | **Z 71** | -9.40 | -One coordination bond with Zn^+2^  -one π-π stacking with HIS401  -three H-bonds with TYR420, ALA189, LEU188 | -6.91 | -Two coordination bonds with Zn^+2^ ion  -Two π-π stacking with HIS401 and TYR423  -Three H-bonds with GLU 402 and MET 422 |  | [19] |
|  | **Z 55** | -9.18 | - one π-cation with Zn^+2^  -two coordination bonds with Zn^+2^  -Two π-π stacking with HIS411 and HIS401  -Four H-bonds with GLN402, ALA189, LEU188 | -4.74 | -two coordination bonds with Zn^+2^ ion  -two π-cation bonds with Zn^+2^  -Two H-bonds with GLU 402 and ALA189 |  | [33] |
|  | **Z 1** | -9.09 | -two coordination bonds with Zn^+2^  -two H-bonds with GLN402, ALA189 | -6.06 | -two coordination bonds with Zn^+2^ ion  -sex H-bonds with LEU188, GLU 402, GLY 186, TYR423, and PRO421 |  | [34] |
|  | **Z 139** | -9.07 | - one π-cation with Zn^+2^  -two coordination bonds with Zn^+2^  -four H-bonds with GLN402, ALA189, LEU188 | -3.33 | -two coordination bonds with Zn^+2^ ion  -Three H-bonds with GLU 402 and ALA189 |  | [35] |
|  | **Z 136** | -8.97 | - one π-cation with ZN^+2^  -two coordination bonds with ZN^+2^  -five H-bonds with LEU397, GLN402, ALA189, LEU188 | -4.26 | -three coordination bonds with Zn^+2^ ion  -four H-bonds with ALA189, GLY 186, and TYR423 |  | [10] |
|  | **Z 124** | -8.88 | - one π-cation with Zn^+2^  -two coordination bonds with Zn^+2^  -one π-π stacking with HIS411  -two H-bonds with ALA189, LEU188 | -3.48 | -One H-bond with TYR420 |  | [5] |
|  | **Z 169** | -8.88 | - one π-cation with Zn^+2^  -one coordination bond with Zn^+2^  -one π-π stacking with HIS401  -two H-bonds with ALA189, HIS190 | -3.51 | -One coordination bond with Zn^+2^ ion  -Two H-bonds with LEU188TYR420 |  | [17] |
|  | **Z 132** | -8.79 | - one π-cation with Zn^+2^  -two coordination bonds with Zn^+2^  -Four H-bonds with GLN402, ALA189, LEU188 | -5.32 | -two coordination bonds with Zn^+2^ ion  -Four H-bonds with LEU188, GLU 402, and TYR423 |  | [36] |
|  | **Z 36** | -8.79 | -two coordination bonds with Zn^+2^  -three π-π stacking with TYR423, HIS401, PHE110  -three H-bonds with GLN402, ALA189 | -4.87 | -one π-π stacking with HIS401  -one H-bond with PRO421 |  | [16] |
|  | **Z 164** | -8.63 | - one π-cation with ZN^+2^  -one π-π stacking with HIS411  -One H-bond with PRO421 | -4.54 | -two coordination bonds with Zn^+2^ ion  -one H-bond with GLU 402 |  | [37] |
|  | **Z 167** | -8.63 | - one π-cation with ZN^+2^  -two coordination bonds with ZN^+2^  -one π-π stacking with HIS401  -three H-bonds with GLN402, ALA189, LEU188 | -6.28 | -Two coordination bonds with Zn^+2^ ion  -one π-π stacking with HIS401  -Three H-bonds with LEU188, ALA189, and TYR423 |  | [36] |
|  | **Z 142** | -8.60 | - one π-cation with ZN^+2^  -two coordination bonds with ZN^+2^  -two H-bonds with ALA189, LEU188 | -8.16 | -Two coordination bonds with Zn^+2^ ion  -one π-cation bond with Zn+2  -three H-bonds with LEU188, ALA189, and GLU 402 |  | [21] |
|  | **Z 152** | -8.52 | - one π-cation with ZN^+2^  -two coordination bonds with ZN^+2^  -three H-bonds with GLN402, ALA189, LEU188 | -7.11 | -Two coordination bonds with Zn^+2^ ion  -one π-π stacking with HIS401  -Two H-bonds with ALA189 and LEU188 |  | [38] |
|  | **Z 181** | -8.43 | - one π-cation with ZN^+2^  -two coordination bonds with ZN^+2^  -one π-π stacking with HIS401  -three H-bonds with GLN402, ALA189, LEU188 | -6.73 | -Two coordination bonds with Zn^+2^ ion  -one π-π stacking with HIS401  -One H-bond with LEU188 |  | [39] |
|  | **Z 125** | -8.40 | - one π-cation with Zn^+2^  -two coordination bonds with Zn^+2^  -three H-bonds with GLN402, ALA189, LEU188 | -2.74 | -One coordination bond with Zn^+2^ ion |  | [6] |
|  | **Z 163** | -8.31 | - one π-cation with ZN^+2^  -one coordination bond with ZN^+2^  -one π-π stacking with TYR423  -Two H-bonds with PRO421, LEU188 | -8.56 | -One coordination bond with Zn^+2^ ion  -one π-π stacking with HIS401  -three H-bonds with ARG 424, GLU 402, and ALA189 |  | [40] |
|  | **Z 13** | -8.26 | -one coordination bond with Zn^+2^  -one H-bond ALA189 | -5.34 | -one π-cation bond with Zn^+2^  -Two π-π stacking with HIS401 and TYR423  -One H-bond with PRO421 |  | [41] |
|  | **Z 123** | -8.22 | -one coordination bond with ZN^+2^ | -7.71 | -One coordination bond with Zn^+2^ ion  -one π-π stacking with HIS401  -One H-bond with LEU188 |  | [42] |
|  | **Z 33** | -8.16 | -one coordination bond with Zn^+2^  -three H-bonds with GLN402, ALA189, LEU188 | -7.26 | -Two coordination bonds with Zn^+2^ ion  -Four H-bonds with LEU188, ALA189, and GLU 402 |  | [43] |
|  | **Z 129** | -8.15 | -one π-π stacking with HIS411 | -5.88 | -Two π-π stacking with HIS401  -Three H-bonds with LEU 418, TYR420, and ALA189 |  | [8] |
|  | **Z 172** | -8.09 | -one π-cation with Zn^+2^  -two coordination bonds with Zn^+2^  -four H-bonds with GLN402, ALA189, LEU188 | -4.92 | -two coordination bonds with Zn^+2^ ion  -Two H-bonds with GLU 402, and ALA189 |  | [44] |
|  | **Z 70** | -8.07 | -One coordination bond  with Zn^+2^  -three H-bonds with TYR420, LEU418, ARG424 | -5.17 | -One coordination bond with Zn^+2^ ion  -Three H-bonds with GLU402, TYR420, and MET422 |  | [2] |
|  | **Z 2** | -7.91 | -one π-cation with Zn^+2^  -two coordination bonds with Zn^+2^  -one π-π stacking with HIS401  -three H-bonds with GLN402, ALA189, LEU188 | -4.60 | -two coordination bonds with Zn^+2^ ion  -one H-bond with ALA189 |  | [45] |
|  | **Z 148** | -7.81 | -one π-cation with Zn^+2^  -two coordination bonds with Zn^+2^  -Five H-bonds with PRO421, ALA189, LEU188, HIS190 | -5.55 | -One coordination bond with Zn^+2^ ion  -one π-π stacking with HIS401  -one H-bond with PRO421 |  | [46] |
|  | **Z 137** | -7.80 | -one π-cation with Zn^+2^  -two coordination bonds with Zn^+2^  -Four H-bonds with GLN402, ALA189, LEU188, PRO421 | -3.47 | -One coordination bond with Zn^+2^ ion  -Two H-bonds with PRO421 |  | [10] |
|  | **Z 173** | -7.78 | -one π-cation with ZN^+2^  -two coordination bonds with ZN^+2^  -two H-bonds with ALA189, LEU188 | -8.34 | -Two coordination bonds with Zn^+2^ ion  -one π-cation bond with Zn^+2^  -One π-π stacking with HIS411  -Two H-bonds with PRO421 and LEU188 |  | [47] |
|  | **Z 26** | -7.73 | -one π-cation with Zn^+2^  -two coordination bonds with Zn^+2^  -two H-bonds with ALA189, LEU188 | -4.20 | -two coordination bonds with Zn^+2^ ion  -one π-cation bond with Zn^+2^  -Two H-bonds with LEU188, GLU 402, and ALA189 |  | [48] |
|  | **Z 128** | -7.63 | -two coordination bonds with ZN^+2^  -one π-π stacking with TYR423  -two H-bonds with GLN402, ALA189 | -9.61 | -Two coordination bonds with Zn^+2^  ion  -one π-cation bond with Zn^+2^  -Four H-bonds with GLU 402, ALA189, and LEU188 |  | [7] |
|  | **Z 16** | -7.58 | -one π-cation with Zn^+2^  -one coordination bond with Zn^+2^  -one π-π stacking with HIS401  -four H-bonds with GLN402, LEU188, PRO421, HIS411 | -7.05 | -One coordination bond with Zn^+2^ ion  -one π-π stacking with TYR423  -Three H-bonds with PRO421, GLU 402, and LEU188 |  | [49] |
|  | **Z 4** | -7.57 | -two π-cation with Zn^+2^  -one coordination bond with Zn^+2^  -two π-π stacking with HIS401, HIS411  -two H-bonds with GLN402, ALA189 | -6.90 | -Two coordination bonds with Zn^+2^ ion  -Two π-π stacking with TYR179 and PHE 192  -Three H-bonds with PRO421, LEU188, and ALA189 |  | [50] |
|  | **Z 109** | -7.56 | -one π-cation with ZN^+2^  -two coordination bonds with ZN^+2^  -four H-bonds with GLN402, ALA189, LEU188 | -6.21 | -two coordination bonds with Zn^+2^ ion  -one π-cation bond with Zn^+2^  -one π-π stacking with HIS401  -five H-bonds with LEU188, GLU 402, and ALA189 |  | [3] |
|  | **Z 23** | -7.50 | -one π-cation with Zn^+2^  -One coordination bond with Zn^+2^  -one π-π stacking with HIS401  -three H-bonds with PRO421, ALA189, LEU188 | -4.08 | -One coordination bond with Zn^+2^ ion  -Three H-bonds with LEU188, PRO421, and ALA189 |  | [51] |
|  | **Z 131** | -7.46 | -Two π-π stacking with HIS401, TYR423  -One H-bond with PRO421 | -3.89 | -two coordination bonds with Zn^+2^ ion |  | [15] |
|  | **Z 184** | -7.42 | -One coordination bonds with ZN^+2^  -one π-π stacking with HIS401  -sex H-bonds with GLN402, ALA189, LEU188, PRO421, ACE109, ASP 410 | -6.48 | -Two coordination bonds with Zn^+2^ ion  -Three H-bonds with ALA189, GLY 186, and TYR423 |  | [52] |
|  | **Z 8** | -7.29 | -one π-π stacking with HIS401  -two H-bonds with LEU188, ACE109 | -3.44 | -two coordination bonds with Zn^+2^ ion |  | [53] |
|  | **Z 138** | -7.14 | -one π-cation with ZN^+2^  -One coordination bond with ZN^+2^  -three H-bonds with PRO421, ALA189, LEU188 | -6.95 | -Two coordination bonds with Zn^+2^ ion  -one π-cation bond with Zn^+2^  -four H-bonds with PRO421, LEU188, ALA189, and GLU 402 |  | [10] |
|  | **Z 17** | -7.06 | -One coordination bonds with Zn^+2^  -one π-π stacking with HIS401  -Four H-bonds with PRO421, GLN402, ALA189, LEU188 | -6.23 | -two coordination bonds with Zn^+2^ ion  -one π-π stacking with HIS401  -one H-bond with LEU188 |  | [50] |
|  | **Z 114** | -7.03 | -Two π-π stacking with HIS401, TYR179  -two H-bonds with ALA189, PRO421 | -8.05 | -Three H-bonds with PRO421, TYR420, and LEU188 |  | [41] |
|  | **Z 118** | -6.93 | -One coordination bond with ZN^+2^  -Two H-bonds with PRO421, TYR420 | -6.34 | -One coordination bond with Zn^+2^ ion  -Two π-π stacking with HIS401 and PHE 192 |  | [54] |
|  | **Z 161** | -6.91 | No interaction | -4.62 | -Three π-π stacking with HIS411, HIS401 and HIS 405  -One H-bond with GLU 402 |  | [55] |
|  | **Z 22** | -6.89 | -one π-π stacking with HIS401  -one H-bond with PRO421 | -6.36 | -one coordination bonds with Zn^+2^ ion  -Three H-bonds with LEU188, GLU 402, and TYR420 |  | [53] |
|  | **Z 69** | -6.79 | -two coordination bonds with Zn^+2^ | -7.29 | -One coordination bond with Zn^+2^ ion  -two H-bonds with GLU 402 |  | [56] |
|  | **Z 187** | -6.78 | -one π-cation with ZN^+2^  -two H-bonds with ALA189, LEU188 | -6.60 | -One coordination bonds with Zn^+2^ ion  -one π-π stacking with HIS401 |  | [57] |
|  | **Z 110** | -6.73 | -one π-π stacking with HIS401  -two H-bonds with LEU188, PRO421 | -5.22 | -One coordination bond with Zn^+2^ ion  -one π-π stacking with TYR423  -Three H-bonds with GLU 402, and ALA189 |  | [3] |
|  | **Z 145** | -6.62 | -two π-cation with Zn^+2^  -one π-π stacking with HIS411  -three H-bonds with ALA189, LEU188, ACE109 | -2.78 | -one π-π stacking with HIS401  -One H-bond with ASP 182 |  | [22] |
|  | **Z 168** | -6.57 | -one π-cation with Zn+2  -two coordination bonds with Zn+2  -three H-bonds with GLN402, ALA189, LEU188 | -5.42 | -two coordination bonds with Zn^+2^ ion  -Two H-bonds with LEU188 and GLU 402 |  | [17] |
|  | **Z 7** | -6.50 | -two coordination bonds with Zn^+2^  -two π-π stacking with TYR423, PHE110  -two H-bonds with GLN402, ALA189 | -7.11 | -Two coordination bonds with Zn^+2^ ion  -one π-π stacking with HIS401  -Two H-bonds with GLU 402 |  | [16] |
|  | **Z 150** | -6.44 | -one π-cation with ZN^+2^  -one coordination bonds with ZN^+2^  -one π-π stacking with HIS401  -three H-bonds with GLN402, ALA189, LEU188 | -5.09 | -One coordination bonds with Zn^+2^ ion  -one π-cation bond with Zn^+2^  -one H-bond with LEU188 |  | [58] |
|  | **Z 25** | -6.43 | -one coordination bond with Zn^+2^  -one π-π stacking with HIS401  -one H-bond with LEU188 | -8.06 | -one π-π stacking with HIS401  -three H-bonds with LEU188 and ALA189 |  | [59] |
|  | **Z 34** | -6.40 | -one π-cation with Zn^+2^  -Two π-π stacking with HIS401, TYR423 | -7.37 | -One coordination bond with Zn^+2^ ion  -one π-π stacking with HIS401 |  | [15] |
|  | **Z 116** | -6.37 | -one coordination bond with ZN^+2^  -one H-bond with LEU188 | -8.39 | -one π-π stacking with HIS401  -Three H-bonds with ALA189 and LEU188 |  | [60] |
|  | **Z 182** | -6.36 | -one coordination bond with ZN^+2^  -three π-π stacking with HIS401, HIS411, TYR423  -two H-bonds with ACE109, MET 422 | -4.64 | -two coordination bonds with Zn^+2^ ion |  | [61] |
|  | **Z 178** | -6.29 | -one π-cation with Zn^+2^  -two coordination bonds with Zn^+2^  -one π-π stacking with HIS401  -One H-bond with ALA189 | -2.19 | -one coordination bond with Zn^+2^ ion  -one π-π stacking with HIS401  -two H-bonds with GLU 402, and ALA191 |  | [51] |
|  | **Z 3** | -6.29 | -two coordination bonds with Zn^+2^  -three H-bonds with GLN402, PRO421, LEU188 | -5.29 | -two coordination bonds with Zn^+2^ ion  -Two H-bonds with ALA191 and TYR423 |  | [62] |
|  | **Z 146** | -6.27 | -One coordination bonds with ZN^+2^  -three H-bonds with GLN402, TYR420, PRO421 | -9.19 | -One coordination bond with Zn^+2^  ion  -four H-bonds with PRO421, LEU188, GLY 186, and GLU 402 |  | [63] |
|  | **Z 108** | -6.26 | -One π-cation with Zn^+2^  -One coordination bond with Zn^+2^  -Three H-bonds with GLN402, ALA189, LEU188 | -4.17 | -One coordination bond with Zn^+2^ ion  -Two salt bridges with Zn^+2^ and GLU 402  -One π-π stacking with HIS401  -One H-bond with TYR423 |  | [64] |
|  | **Z 149** | -6.26 | -One coordination bonds with ZN^+2^  -three H-bonds with GLN402, ALA189, PRO421 | -6.63 | -Two coordination bonds with Zn^+2^ ion  -Three H-bonds with GLU 402, GLY 186, and TYR423 |  | [65] |
|  | **Z 130** | -6.25 | -Three π-π stacking with HIS401, PHE110, TYR423  -One H-bonds with ALA189 | -6.79 | -One coordination bond with Zn^+2^ ion  -One H-bond with ALA189 |  | [15] |
|  | **Z 155** | -6.22 | -one π-π stacking with HIS401  -three H-bonds with MET 422, PRO421, TYR420 | -5.39 | -one coordination bond with Zn^+2^ ion  -one H-bond with TYR423 |  | [24] |
|  | **Z 89** | -6.18 | -Two π-π stacking with HIS401  -two H-bonds with PRO421, LEU188 | -4.67 | -One coordination bond with Zn^+2^ ion  -one H-bond with LEU188 |  |  |
|  | **Z 185** | -6.08 | -One coordination bonds with ZN^+2^  -two π-π stacking with HIS401, HIS411 | -7.61 | -One coordination bond with Zn^+2^ ion  -two π-π stacking with HIS401 and HIS411  -Three H-bonds with LEU188, ALA189, and GLU 402 |  | [66] |
|  | **Z 135** | -6.07 | -one π-π stacking with TYR423  -One H-bonds with ALA189 | -5.21 | -Two H-bonds with ARG 424 and ALA 417 |  | [9] |
|  | **Z 179** | -6.07 | -One coordination bond with ZN^+2^  -one π-π stacking with HIS401  -three H-bonds with GLN402, ARG 424, LEU188 | -7.14 | -One coordination bond with Zn^+2^ ion  -one π-π stacking with HIS401  -two H-bonds with PRO421 and ARG 424 |  | [67] |
|  | **Z 29** | -6.00 | -Two π-cation with Zn^+2^  -two coordination bonds with Zn^+2^  -three H-bonds with ALA189, LEU188 | -2.90 | -one coordination bonds with Zn^+2^ ion  -Two H-bonds with LEU188 and PRO421 |  | [68] |
|  | **Z 154** | -5.94 | -one π-cation with ZN^+2^  -three H-bonds with PRO421, LEU 418, ARG 424 | -5.62 | -one coordination bond with Zn^+2^ ion  -Two H-bonds with ALA189 and TYR423 |  | [24] |
|  | **Z 127** | -5.92 | -one coordination bond with ZN^+2^  -one π-π stacking with TYR423  -two H-bonds with GLN402, LEU188 | -8.57 | -Two coordination bonds with Zn^+2^  ion  -One H-bond with LEU188 |  | [7] |
|  | **Z 113** | -5.91 | -one π-cation with ZN^+2^  -one π-π stacking with HIS401  -two H-bonds with PRO421, LEU188 | -6.93 | -Two coordination bonds with Zn^+2^ ion  -Two π-π stacking with HIS411  -two H-bonds with TYR420 and MET 422 |  | [11] |
|  | **Z 27** | -5.68 | -one coordination bonds with Zn^+2^  -one π-π stacking with TYR423  -five H-bonds with GLN402, ALA189, LEU188 | -3.96 | -one coordination bonds with Zn^+2^ ion  -Two H-bonds with LEU188 and PRO421 |  | [69] |
|  | **Z 11** | -5.66 | -One H-bond with PRO421 | -6.13 | -Two coordination bonds with Zn^+2^ ion  -one π-π stacking with HIS401  -Three H-bonds with LEU188 and GLU 402 |  | [25] |
|  | **Z 15** | -5.66 | -three π-π stacking with HIS401, HIS411  -One H-bond with PRO421 | -7.86 | -One coordination bond with Zn^+2^ ion  -two π-π stacking with HIS401 and TYR423  -Three H-bonds with PRO421, GLU 402, and LEU188 |  | [70] |
|  | **Z 119** | -5.65 | -Two coordination bonds with ZN^+2^  -Three π-π stacking with HIS411, TYR179 | -7.29 | -Two coordination bonds with Zn^+2^ ion  -Two π-π stacking with HIS401  -One H-bond with GLU 402 |  | [54] |
|  | **Z 39** | -5.63 | -one π-cation with Zn^+2^  -two coordination bonds with Zn^+2^  -three H-bonds with GLN402, ALA189, LEU188 | -6.43 | -one coordination bonds with Zn^+2^ ion  -one π-cation bond with Zn^+2^  -One H-bond with LEU188 |  | [71] |
|  | **Z 171** | -5.62 | -one π-cation with ZN^+2^  -one π-π stacking with HIS401  -One H-bond with TYR420 | -5.76 | -One π-π stacking with HIS401  -one H-bond with LEU 418 |  | [72] |
|  | **Z 50** | -5.58 | -one coordination bond with Zn^+2^  -two π-π stacking with HIS411, TYR179  -three H-bonds with GLN402, ALA189, LEU188 | -6.13 | -One π-π stacking with HIS401  -Three H-bonds with LEU188, GLY 186, and TYR423 |  | [54] |
|  | **Z 162** | -5.56 | -One coordination bond  With Zn^+2^  -one π-π stacking with HIS401 | -4.59 | -one π-cation bond with Zn^+2^  -three π-π stacking with HIS401 and HIS411 |  | [40] |
|  | **Z 12** | -5.53 | -one π-cation with Zn^+2^  -two coordination bonds with Zn^+2^  -two H-bonds with ALA189, LEU188 | -5.72 | -two coordination bonds with Zn^+2^ ion  -one π-cation bond with Zn^+2^  -one π-π stacking with HIS401  -Three H-bonds with LEU188, GLU 402, and ALA189 |  | [73] |
|  | **Z 140** | -5.51 | -one π-cation with ZN^+2^  -two π-π stacking with HIS401, HIS411 | -4.11 | -two coordination bonds with Zn^+2^ ion |  | [21] |
|  | **Z 176** | -5.39 | -one π-cation with Zn^+2^  -two coordination bonds with Zn^+2^  -two H-bonds with ALA189, LEU188 | -5.06 | -two coordination bonds with Zn^+2^ ion  -one π-π stacking with HIS411  -one H-bond with LEU188 |  | [45] |
|  | **Z 37** | -5.34 | One π-cation with Zn^+2^  -two coordination bonds with Zn^+2^  -one π-π stacking with HIS TYR423  -two H-bonds with ALA189 | -5.69 | -Three coordination bonds with Zn^+2^ ion  -One H-bond with ALA189 |  | [70] |
|  | **Z 115** | -5.24 | -one π-cation with Zn^+2^  -Three, π-π stacking with HIS401, PHE110  -One H-bond with PRO421 | -4.05 | -one coordination bond with Zn^+2^ ion  -one π-cation bond with PHE 192 |  | [39] |
|  | **Z 41** | -5.24 | -one π-cation with Zn^+2^  -two coordination bonds with Zn^+2^  -two H-bonds with ALA189, LEU188 | -3.95 | -one coordination bonds with Zn^+2^ ion  -Three H-bonds with LEU188, TYR423, and PRO421 |  | [41] |
|  | **Z 117** | -5.14 | -One coordination bond with ZN^+2^  -Two π-π stacking with HIS411, TYR179  -One H-bond with TYR420 | -6.30 | -Two coordination bonds with Zn^+2^ ion  -One π-π stacking with HIS401 |  | [54] |
|  | **Z 19** | -5.10 | -one π-cation with Zn^+2^  -One coordination bonds with Zn^+2^  -one π-π stacking with HIS401  -five H-bonds with GLN402, ALA189, LEU188, PRO421 | -5.44 | -One coordination bonds with Zn^+2^ ion  -one π-cation bond with Zn^+2^  -one π-π stacking with HIS401  -Three H-bonds with LEU188, GLU 402, and PRO421 |  | [74] |
|  | **Z 160** | -5.01 | Two π-π stacking with HIS411, TYR423  -one H-bond with TYR420 | -5.65 | -one π-π stacking with HIS401  -One H-bond with TYR423 |  | [55] |
|  | **Z 9** | -4.95 | -one π-cation with Zn^+2^  -Two π-π stacking with HIS401, PHE110  -two H-bonds with ALA189, LEU188 | -6.11 | -two coordination bonds with Zn^+2^ ion  -one π-cation bond with Zn^+2^  -one π-π stacking with HIS401  -Three H-bonds with ALA189 and LEU188 |  | [17] |
|  | **Z 21** | -4.94 | -two π-π stacking with HIS401, TYR423 | -3.45 | -One coordination bonds with Zn^+2^ ion  -one H-bond with PRO421 |  | [75] |
|  | **Z 111** | -4.85 | -One π-cation with Zn^+2^  -One coordination bond with Zn^+2^  -one π-π stacking with TYR423  -three H-bonds with GLN402, ALA189, LEU188  -One halogen bond with ALA 417 | -7.72 | -one π-π stacking with TYR423  -Four H-bonds with TYR423, ALA189, LEU188, and GLY 186 |  | [76] |
|  | **Z 46** | -4.85 | -one π-cation with Zn^+2^  -one coordination bonds with Zn^+2^  -one π-π stacking with HIS411  -two H-bonds with ALA189, LEU188  -One halogen bond with ARG 424 | -6.36 | -one coordination bond with Zn^+2^ ion  -one π-cation bond with Zn^+2^  -Two π-π stacking with HIS401 and HIS411  -Two H-bonds with LEU188 and GLU 402 |  | [77] |
|  | **Z 45** | -4.83 | -one π-cation with Zn^+2^  -one coordination bond with Zn^+2^  -one π-π stacking with HIS411  -two H-bonds with ALA189, LEU188  -One halogen bond with ARG 424 | -6.36 | -one coordination bond with Zn^+2^ ion  -one π-cation bond with Zn^+2^  -Two π-π stacking with HIS401 and HIS411  -Two H-bonds with LEU188 and GLU 402 |  | [77] |
|  | **Z 122** | -4.82 | -two coordination bonds with ZN^+2^  -two H-bonds with GLN402, PRO421 | -6.41 | -four H-bonds with LEU188, GLU 402, PRO421, and TYR423 |  | [20] |
|  | **Z 112** | -4.79 | -one π-cation with Zn^+2^  -one π-π stacking with HIS401  -One H-bond with PRO421 | -3.48 | -two coordination bonds with Zn^+2^ ion  -two H-bonds with LEU188 and PRO421 |  | [78] |
|  | **Z 144** | -4.77 | -one coordination bonds with Zn^+2^  -one π-π stacking with TYR423 | -6.16 | -two coordination bonds with Zn^+2^ ion  -one H-bond with ALA189 |  | [22] |
|  | **Z 24** | -4.77 | -one π-cation with Zn^+2^  -one π-π stacking with HIS401 | -7.46 | -Three H-bonds with ALA189 and LEU188 |  | [51] |
|  | **Z 177** | -4.76 | -one π-cation with Zn^+2^ | -6.09 | -Two H-bonds with LEU188 and GLY 186 |  | [36] |
|  | **Z 165** | -4.73 | -one π-cation with Zn^+2^ | -4.79 | -Two H-bonds with GLU 402 and ALA189 |  | (136) |
|  | **Z 91** | -4.70 | -one π-π stacking with HIS401  -two H-bonds with ALA189, LEU188 | -7.05 | -One coordination bond with Zn^+2^ ion  -Two π-π stacking with HIS401 and TYR423  -One H-bond with TYR420 |  | [25] |
|  | **Z 159** | -4.68 | -one π-π stacking with HIS401  -one H-bond with TYR420 | -5.67 | -one π-cation bond with Zn^+2^  -two π-π stacking with HIS401 and HIS411  -One H-bond with ALA191  -three halogen bonds with LEU188 and ALA189 |  | [80] |
|  | **Z 183** | -4.61 | -One coordination bonds with ZN^+2^  -one π-π stacking with HIS411  -one H-bond with GLN402 | -6.59 | -Two coordination bonds with Zn^+2^ ion  -Six H-bonds with LEU188, GLU 402, GLY 186, TYR423, and PRO421 |  | [72] |
|  | **Z 153** | -4.59 | -Three π-π stacking with HIS411, HIS401, PHE110  -One H-bond with HIS190 | -4.68 | -one coordination bond with Zn^+2^ ion  -two π-π stacking with HIS 405 and HIS411 |  | [23] |
|  | **Z 158** | -4.49 | One π-cation with ZN^+2^  -Four π-π stacking with HIS411, HIS401, TYR423  -One H-bonds with ALA189 | -4.86 | -Three π-π stacking with HIS401 and HIS411  -One H-bond with ALA189 |  | [81] |
|  | **Z 38** | -4.43 | -one π-π stacking with TYR179  -One H-bond with LEU188 | -5.11 | -One coordination bonds with Zn^+2^ ion  -one π-cation bond with Zn^+2^  -one π-π stacking with HIS401  -Two H-bonds with ALA191 |  | [82] |
|  | **Z 44** | -4.42 | -one π-cation with Zn^+2^  -one π-π stacking with HIS401  -three H-bonds with ALA189, LEU188, PRO421 | -5.04 | -one coordination bond with Zn^+2^ ion  -one π-π stacking with HIS401 |  | [77] |
|  | **Z 156** | -4.28 | -two π-π stacking with HIS411, PHE110  -two H-bonds with HIS411, ASP 410 | -4.35 | -one π-cation bond with Zn^+2^  -two π-π stacking with HIS401 and HIS411  -One H-bond with TYR423 |  | (146) |
|  | **Z 30** | -4.19 | -one π-cation with Zn^+2^  -Two π-π stacking with HIS411  -one H-bond with PRO421 | -4.76 | -one H-bond with TYR 393 |  | [83] |
|  | **Z 157** | -4.10 | -two H-bonds with ASP 410, HIS411 | -4.58 | -two coordination bonds with Zn^+2^ ion  -one π-π stacking with HIS411  -one H-bond with TYR423 |  | [3] |
|  | **Z 151** | -4.08 | -one π-cation with ZN^+2^  -one π-π stacking with HIS401  -One H-bond with HIS411 | -8.35 | -four H-bonds with ALA189, LEU188, and TYR423 |  | [61] |
|  | **Z 126** | -3.89 | No interaction | -2.95 | -One coordination bond with Zn^+2^ ion |  | [6] |
|  | **Z 134** | -3.89 | -One coordination bond with ZN^+2^  -one π-π stacking with HIS401  -one H-bond with GLN402 | -8.37 | -One coordination bond with Zn^+2^ ion  -one π-π stacking with HIS401  -two H-bonds with TYR423 and GLY 186 |  | [84] |
|  | **Z 175** | -3.88 | -two coordination bonds with ZN^+2^  -one π-π stacking with PHE110  -Four H-bonds with ALA189, LEU188, PRO421, GLN402 | -6.83 | -Two coordination bonds with Zn^+2^ ion  -Three H-bonds with PRO421, GLU 402, and LEU188 |  | [39] |
|  | **Z 49** | -3.52 | -one π-cation with HIS401  -One coordination bond with Zn^+2^  -Three π-π stacking with HIS401, HIS411, PHE110 | -5.64 | -one π-cation bond with Zn^+2^  -one π-π stacking with HIS401  -Two H-bonds with LEU188 and PRO421 |  | [18] |
|  | **Z 121** | -3.49 | -One coordination bonds with ZN^+2^  -two H-bonds with PRO421, GLN402 | -6.60 | -one π-cation bond with Zn^+2^  -Two π-π stacking with HIS411 and HIS401  -Three H-bonds with LEU188 and PRO421 |  | [20] |
|  | **Z 31** | -3.21 | -One coordination bonds with Zn^+2^  -one π-π stacking with HIS411  -One H-bond with HIS411 | -4.18 | -Two H-bonds with GLY186 and TYR423 |  | [85] |
|  | **Z 43** | -2.96 | One π-cation with Zn^+2^  -two coordination bonds with Zn^+2^  -three H-bonds with ALA189, LEU188, PRO421 | -6.08 | -two coordination bonds with Zn^+2^ ion  -one π-cation bond with Zn^+2^  -Three H-bonds with LEU188 and ALA189 |  | [17] |
|  | **Z 120** | -2.92 | -One coordination bond with ZN^+2^  -Two H-bonds with PRO421, GLN402 | -5.63 | -Two H-bonds with LEU188 and GLY 186 |  | [86] |
|  | **Z 40** | -2.83 | -one π-cation with Zn^+2^  -two H-bonds with ALA189, LEU188 | -5.24 | -One coordination bonds with Zn^+2^ ion  -one π-π stacking with HIS411  -four H-bonds with LEU188, GLU 402, and HIS411 |  | [23] |
|  | **Z 180** | -2.82 | -one π-π stacking with HIS401  -three H-bonds with ALA189, PRO421, GLN402 | -7.50 | -One π-cation bond with Zn^+2^  -Two π-π stacking with HIS411 and HIS401  -four H-bonds with GLU 402, ALA191, ALA189, and LEU188 |  | [87] |
|  | **Z 32** | -2.64 | One π-cation with Zn^+2^  -one π-π stacking with HIS411  -One H-bonds with ALA189 | -3.49 | -One coordination bonds with Zn^+2^ ion  -one π-cation bond with Zn^+2^  -one H-bond with PRO421 |  | [69] |
|  | **Z 47** | -2.42 | One π-cation with HIS401  -one π-π stacking with HIS411 | -4.92 | -one π-π stacking with HIS401  -one H-bond with PRO421 |  | [18] |
|  | **Z 166** | -1.97 | One π-cation with Zn^+2^  -one coordination bond with Zn^+2^  -one π-π stacking with HIS401  -two H-bonds with ALA189, GLN402  -One halogen bond with HIS411 | -3.87 | -One coordination bond with Zn^+2^ ion  -two π-π stacking with HIS401 and TYR423  -one H-bond with PRO421 |  | [88] |
|  | **Z 48** | -1.62 | One π-cation with HIS401  -one π-π stacking with HIS401  -One coordination bond with Zn^+2^ | -4.64 | -two salt bridges with Zn^+2^ ion  -two π-π stacking with HIS401 and HIS411  -Three H-bonds with LEU188, ALA191, and PRO421 |  | [18] |

References:

1. Yamamoto M, Tsujishita H, Hori N, Ohishi Y, Inoue S, Ikeda S, et al. Inhibition of Membrane-Type 1 Matrix Metalloproteinase by Hydroxamate Inhibitors:  An Examination of the Subsite Pocket. J Med Chem. 1998;41: 1209–1217. doi:10.1021/jm970404a

2. Gao Q, Wang Y, Hou J, Yao Q, Zhang J. Multiple receptor-ligand based pharmacophore modeling and molecular docking to screen the selective inhibitors of matrix metalloproteinase-9 from natural products. J Comput Aided Mol Des. 2017;31: 625–641. doi:10.1007/s10822-017-0028-3

3. Lenci E, Innocenti R, Di Francescantonio T, Menchi G, Bianchini F, Contini A, et al. Identification of highly potent and selective MMP2 inhibitors addressing the S1’ subsite with d-proline-based compounds. Bioorg Med Chem. 2019;27: 1891–1902. doi:10.1016/j.bmc.2019.03.043

4. Erdeljac N, Thiehoff C, Jumde RP, Daniliuc CG, Höppner S, Faust A, et al. Validating the 1,2-Difluoro Motif As a Hybrid Bioisostere of CF3 and Et Using Matrix Metalloproteinases As Structural Probes. J Med Chem. 2020;63: 6225–6237. doi:10.1021/acs.jmedchem.0c00648

5. Chollet A-M, Le Diguarher T, Murray L, Bertrand M, Tucker GC, Sabatini M, et al. General synthesis of α-substituted 3-bisaryloxy propionic acid derivatives as specific mmp inhibitors. Bioorg Med Chem Lett. 2001;11: 295–299. doi:10.1016/S0960-894X(00)00646-6

6. Pikul S, Ohler NE, Ciszewski G, Laufersweiler MC, Almstead NG, De B, et al. Potent and Selective Carboxylic Acid-Based Inhibitors of Matrix Metalloproteinases. J Med Chem. 2001;44: 2499–2502. doi:10.1021/jm015531s

7. Sørensen MD, Blaehr LKA, Christensen MK, Høyer T, Latini S, Hjarnaa P-JV, et al. Cyclic phosphinamides and phosphonamides, novel series of potent matrix metalloproteinase inhibitors with antitumour activity. Bioorg Med Chem. 2003;11: 5461–5484. doi:10.1016/j.bmc.2003.09.015

8. Wang X, Choe Y, Craik CS, Ellman JA. Design and synthesis of novel inhibitors of gelatinase B. Bioorg Med Chem Lett. 2002;12: 2201–2204. doi:10.1016/s0960-894x(02)00365-7

9. Ikura M, Nakatani S, Yamamoto S, Habashita H, Sugiura T, Takahashi K, et al. Discovery of a new chemical lead for a matrix metalloproteinase inhibitor. Bioorg Med Chem. 2006;14: 4241–4252. doi:10.1016/j.bmc.2006.01.059

10. Leutert T, Grob JE, Tommasi RA, Pusateri EE, Honda A. Selective hydroxamate based MMP inhibitors. US8232427B2, 2012. Available: https://patents.google.com/patent/US8232427B2/en

11. Bianchini F, Calugi C, Ruzzolini J, Menchi G, Calorini L, Guarna A, et al. A study of a D-proline peptidomimetic inhibitor of melanoma and endothelial cell invasion through activity towards MMP-2 and MMP-9. MedChemComm. 2015;6: 277–282. doi:10.1039/C4MD00287C

12. Sawa M, Kiyoi T, Kurokawa K, Kumihara H, Yamamoto M, Miyasaka T, et al. New Type of Metalloproteinase Inhibitor:  Design and Synthesis of New Phosphonamide-Based Hydroxamic Acids. J Med Chem. 2002;45: 919–929. doi:10.1021/jm0103211

13. Moriyama H, Tsukida T, Inoue Y, Kondo H, Yoshino K, Nishimura S-I. Structure–activity relationships of azasugar-based MMP/ADAM inhibitors. Bioorg Med Chem Lett. 2003;13: 2737–2740. doi:10.1016/S0960-894X(03)00530-4

14. Cook GR, Manivannan E, Underdahl T, Lukacova V, Zhang Y, Balaz S. Synthesis and evaluation of novel oxazoline MMP inhibitors. Bioorg Med Chem Lett. 2004;14: 4935–4939. doi:10.1016/j.bmcl.2004.07.023

15. Le Diguarher T, Chollet A-M, Bertrand M, Hennig P, Raimbaud E, Sabatini M, et al. Stereospecific synthesis of 5-substituted 2-bisarylthiocyclopentane carboxylic acids as specific matrix metalloproteinase inhibitors. J Med Chem. 2003;46: 3840–3852. doi:10.1021/jm0307638

16. Yang S-M, Scannevin RH, Wang B, Burke SL, Huang Z, Karnachi P, et al. β-*N*-Biaryl ether sulfonamide hydroxamates as potent gelatinase inhibitors: Part 2. Optimization of α-amino substituents. Bioorg Med Chem Lett. 2008;18: 1140–1145. doi:10.1016/j.bmcl.2007.11.129

17. Behrends M, Wagner S, Kopka K, Schober O, Schäfers M, Kumbhar S, et al. New matrix metalloproteinase inhibitors based on γ-fluorinated α-aminocarboxylic and α-aminohydroxamic acids. Bioorg Med Chem. 2015;23: 3809–3818. doi:10.1016/j.bmc.2015.03.078

18. Ayoup MS, Fouad MA, Abdel-Hamid H, Ramadan ES, Abu-Serie MM, Noby A, et al. Battle tactics against MMP-9; discovery of novel non-hydroxamate MMP-9 inhibitors endowed with PI3K/AKT signaling attenuation and caspase 3/7 activation *via* Ugi bis-amide synthesis. Eur J Med Chem. 2020;186: 111875. doi:10.1016/j.ejmech.2019.111875

19. Hou J, Zou Q, Wang Y, Gao Q, Yao W, Yao Q, et al. Screening for the selective inhibitors of MMP-9 from natural products based on pharmacophore modeling and molecular docking in combination with bioassay experiment, hybrid QM/MM calculation, and MD simulation. J Biomol Struct Dyn. 2019;37: 3135–3149. doi:10.1080/07391102.2018.1509019

20. Fink CA, Carlson JE, Boehm C, McTaggart P, Qiao Y, Doughty J, et al. Design and synthesis of thiol containing inhibitors of matrix metalloproteinases. Bioorg Med Chem Lett. 1999;9: 195–200. doi:10.1016/S0960-894X(98)00716-1

21. Zhang Y-M, Fan X, Yang S-M, Scannevin RH, Burke SL, Rhodes KJ, et al. Syntheses and in vitro evaluation of arylsulfone-based MMP inhibitors with heterocycle-derived zinc-binding groups (ZBGs). Bioorg Med Chem Lett. 2008;18: 405–408. doi:10.1016/j.bmcl.2007.10.049

22. Nuti E, Cuffaro D, Bernardini E, Camodeca C, Panelli L, Chaves S, et al. Development of Thioaryl-Based Matrix Metalloproteinase-12 Inhibitors with Alternative Zinc-Binding Groups: Synthesis, Potentiometric, NMR, and Crystallographic Studies. J Med Chem. 2018;61: 4421–4435. doi:10.1021/acs.jmedchem.8b00096

23. Topai A, Breccia P, Minissi F, Padova A, Marini S, Cerbara I. In silico scaffold evaluation and solid phase approach to identify new gelatinase inhibitors. Bioorg Med Chem. 2012;20: 2323–2337. doi:10.1016/j.bmc.2012.02.010

24. Barbay JK, Leonard KA, Zhang Y, Tounge BA, Wang A, Hawkins M, et al. Bis heteroaryl inhibitors of pro-matrix metalloproteinase activation. US20120129897A1, 2012. Available: https://patents.google.com/patent/US20120129897A1/en

25. Nuti E, Cantelmo AR, Gallo C, Bruno A, Bassani B, Camodeca C, et al. N-O-Isopropyl Sulfonamido-Based Hydroxamates as Matrix Metalloproteinase Inhibitors: Hit Selection and in Vivo Antiangiogenic Activity. J Med Chem. 2015;58: 7224–7240. doi:10.1021/acs.jmedchem.5b00367

26. Nuti E, Cuffaro D, D’Andrea F, Rosalia L, Tepshi L, Fabbi M, et al. Sugar-Based Arylsulfonamide Carboxylates as Selective and Water-Soluble Matrix Metalloproteinase-12 Inhibitors. ChemMedChem. 2016;11: 1626–1637. doi:10.1002/cmdc.201600235

27. Tochowicz A, Maskos K, Huber R, Oltenfreiter R, Dive V, Yiotakis A, et al. Crystal structures of MMP-9 complexes with five inhibitors: contribution of the flexible Arg424 side-chain to selectivity. J Mol Biol. 2007;371: 989–1006. doi:10.1016/j.jmb.2007.05.068

28. Yamamoto S, Nakatani S, Ikura M, Sugiura T, Nishita Y, Itadani S, et al. Design and synthesis of an orally active matrix metalloproteinase inhibitor. Bioorg Med Chem. 2006;14: 6383–6403. doi:10.1016/j.bmc.2006.05.040

29. Rossello A, Nuti E, Carelli P, Orlandini E, Macchia M, Nencetti S, et al. N-i-Propoxy-N-biphenylsulfonylaminobutylhydroxamic acids as potent and selective inhibitors of MMP-2 and MT1-MMP. Bioorg Med Chem Lett. 2005;15: 1321–1326. doi:10.1016/j.bmcl.2005.01.024

30. Zhang Y-M, Fan X, Chakaravarty D, Xiang B, Scannevin RH, Huang Z, et al. 1-Hydroxy-2-pyridinone-based MMP inhibitors: Synthesis and biological evaluation for the treatment of ischemic stroke. Bioorg Med Chem Lett. 2008;18: 409–413. doi:10.1016/j.bmcl.2007.10.045

31. Kiyama R, Tamura Y, Watanabe F, Tsuzuki H, Ohtani M, Yodo M. Homology Modeling of Gelatinase Catalytic Domains and Docking Simulations of Novel Sulfonamide Inhibitors. J Med Chem. 1999;42: 1723–1738. doi:10.1021/jm980514x

32. Tamura Y, Watanabe F, Nakatani T, Yasui K, Fuji M, Komurasaki T, et al. Highly Selective and Orally Active Inhibitors of Type IV Collagenase (MMP-9 and MMP-2):  N-Sulfonylamino Acid Derivatives. J Med Chem. 1998;41: 640–649. doi:10.1021/jm9707582

33. Hanessian S, MacKay DB, Moitessier N. Design and Synthesis of Matrix Metalloproteinase Inhibitors Guided by Molecular Modeling. Picking the S1 Pocket Using Conformationally Constrained Inhibitors. J Med Chem. 2001;44: 3074–3082. doi:10.1021/jm010096n

34. Rowsell S, Hawtin P, Minshull CA, Jepson H, Brockbank SMV, Barratt DG, et al. Crystal structure of human MMP9 in complex with a reverse hydroxamate inhibitor. J Mol Biol. 2002;319: 173–181. doi:10.1016/S0022-2836(02)00262-0

35. Wagner S, Breyholz H-J, Law MP, Faust A, Höltke C, Schröer S, et al. Novel fluorinated derivatives of the broad-spectrum MMP inhibitors N-hydroxy-2(R)-[[(4-methoxyphenyl)sulfonyl](benzyl)- and (3-picolyl)-amino]-3-methyl-butanamide as potential tools for the molecular imaging of activated MMPs with PET. J Med Chem. 2007;50: 5752–5764. doi:10.1021/jm0708533

36. Chen C, Yang X, Fang H, Hou X. Design, synthesis and preliminary bioactivity evaluations of 8-hydroxyquinoline derivatives as matrix metalloproteinase (MMP) inhibitors. Eur J Med Chem. 2019;181: 111563. doi:10.1016/j.ejmech.2019.111563

37. Nicolotti O, Catto M, Giangreco I, Barletta M, Leonetti F, Stefanachi A, et al. Design, synthesis and biological evaluation of 5-hydroxy, 5-substituted-pyrimidine-2,4,6-triones as potent inhibitors of gelatinases MMP-2 and MMP-9. Eur J Med Chem. 2012;58: 368–376. doi:10.1016/j.ejmech.2012.09.036

38. Wang J, O’Sullivan S, Harmon S, Keaveny R, Radomski MW, Medina C, et al. Design of Barbiturate–Nitrate Hybrids that Inhibit MMP-9 Activity and Secretion. J Med Chem. 2012;55: 2154–2162. doi:10.1021/jm201352k

39. Tauro M, Laghezza A, Loiodice F, Piemontese L, Caradonna A, Capelli D, et al. Catechol-based matrix metalloproteinase inhibitors with additional antioxidative activity. J Enzyme Inhib Med Chem. 2016;31: 25–37. doi:10.1080/14756366.2016.1217853

40. Jackson PF, Maharoof USM, Leonard KA, Baxter E, Tounge BA, Hawkins M. Benzothiazolyl inhibitors of pro-matrix metalloproteinase activation. WO2012162463A1, 2012. Available: https://patents.google.com/patent/WO2012162463A1/en

41. Hugenberg V, Hermann S, Galla F, Schäfers M, Wünsch B, Kolb HC, et al. Radiolabeled hydroxamate-based matrix metalloproteinase inhibitors: How chemical modifications affect pharmacokinetics and metabolic stability. Nucl Med Biol. 2016;43: 424–437. doi:10.1016/j.nucmedbio.2016.03.005

42. Foley LH, Palermo R, Dunten P, Wang P. Novel 5,5-disubstitutedpyrimidine-2,4,6-triones as selective MMP inhibitors. Bioorg Med Chem Lett. 2001;11: 969–972. doi:10.1016/S0960-894X(01)00104-4

43. Zask A, Gu Y, Albright JD, Du X, Hogan M, Levin JI, et al. Synthesis and SAR of bicyclic heteroaryl hydroxamic acid MMP and TACE inhibitors. Bioorg Med Chem Lett. 2003;13: 1487–1490. doi:10.1016/S0960-894X(03)00127-6

44. Yan Y, Chen X, Yang X, Zhang J, Xu W, Zhang Y. Synthesis of chiral ND-322, ND-364 and ND-364 derivatives as selective inhibitors of human gelatinase. Bioorg Med Chem. 2015;23: 6632–6640. doi:10.1016/j.bmc.2015.09.013

45. Mukherjee A, Adhikari N, Jha T. A pentanoic acid derivative targeting matrix metalloproteinase-2 (MMP-2) induces apoptosis in a chronic myeloid leukemia cell line. Eur J Med Chem. 2017;141: 37–50. doi:10.1016/j.ejmech.2017.09.052

46. Mangiatordi GF, Guzzo T, Rossano EC, Trisciuzzi D, Alberga D, Fasciglione G, et al. Design, Synthesis, and Biological Evaluation of Tetrahydro-β-carboline Derivatives as Selective Sub-Nanomolar Gelatinase Inhibitors. ChemMedChem. 2018;13: 1343–1352. doi:10.1002/cmdc.201800237

47. Beutel B, Daniliuc CG, Riemann B, Schäfers M, Haufe G. Fluorinated matrix metalloproteinases inhibitors--Phosphonate based potential probes for positron emission tomography. Bioorg Med Chem. 2016;24: 902–909. doi:10.1016/j.bmc.2016.01.017

48. Wang J, Medina C, Radomski MW, Gilmer JF. N-Substituted homopiperazine barbiturates as gelatinase inhibitors. Bioorg Med Chem. 2011;19: 4985–4999. doi:10.1016/j.bmc.2011.06.055

49. Selivanova SV, Stellfeld T, Heinrich TK, Müller A, Krämer SD, Schubiger PA, et al. Design, Synthesis, and Initial Evaluation of a High Affinity Positron Emission Tomography Probe for Imaging Matrix Metalloproteinases 2 and 9. J Med Chem. 2013;56: 4912–4920. doi:10.1021/jm400156p

50. K. Halder A, Mallick S, Shikha D, Saha A, D. Saha K, Jha T. Design of dual MMP-2/HDAC-8 inhibitors by pharmacophore mapping, molecular docking, synthesis and biological activity. RSC Adv. 2015;5: 72373–72386. doi:10.1039/C5RA12606A

51. Wada CK, Holms JH, Curtin ML, Dai Y, Florjancic AS, Garland RB, et al. Phenoxyphenyl Sulfone N-Formylhydroxylamines (Retrohydroxamates) as Potent, Selective, Orally Bioavailable Matrix Metalloproteinase Inhibitors. J Med Chem. 2002;45: 219–232. doi:10.1021/jm0103920

52. Hirayama R, Yamamoto M, Tsukida T, Matsuo K, Obata Y, Sakamoto F, et al. Synthesis and biological evaluation of orally active matrix metalloproteinase inhibitors. Bioorg Med Chem. 1997;5: 765–778. doi:10.1016/s0968-0896(97)00028-x

53. Beutel B, Song J, Konken CP, Korpos E, Schinor B, Gerwien H, et al. New in Vivo Compatible Matrix Metalloproteinase (MMP)-2 and MMP-9 Inhibitors. Bioconjug Chem. 2018;29: 3715–3725. doi:10.1021/acs.bioconjchem.8b00618

54. Ayoup MS, Abu-Serie MM, Awad LF, Teleb M, Ragab HM, Amer A. Halting colorectal cancer metastasis via novel dual nanomolar MMP-9/MAO-A quinoxaline-based inhibitors; design, synthesis, and evaluation. Eur J Med Chem. 2021;222: 113558. doi:10.1016/j.ejmech.2021.113558

55. Jackson PF, Manthey C, Rhodes K, Scannevin R, Leonard KA, Barbay JK, et al. Dérivés de thiazole en tant qu’inhibiteurs de pro-métalloprotéinases de matrice. WO2012162468A1, 2012. Available: https://patents.google.com/patent/WO2012162468A1/fr

56. Kalva S, Azhagiya Singam ER, Rajapandian V, Saleena LM, Subramanian V. Discovery of potent inhibitor for matrix metalloproteinase-9 by pharmacophore based modeling and dynamics simulation studies. J Mol Graph Model. 2014;49: 25–37. doi:10.1016/j.jmgm.2013.12.008

57. Laghezza A, Luisi G, Caradonna A, Di Pizio A, Piemontese L, Loiodice F, et al. Virtual screening identification and chemical optimization of substituted 2-arylbenzimidazoles as new non-zinc-binding MMP-2 inhibitors. Bioorg Med Chem. 2020;28: 115257. doi:10.1016/j.bmc.2019.115257

58. Choi S-S, Ji A-R, Yu S-W, Cho B-H, Park J-D, Park J-H, et al. Inhibition of Invasion and Capillary-like Tube Formation by Retrohydroxamate-based MMP Inhibitors. Bull Korean Chem Soc. 2011;32: 2032–2038. doi:10.5012/bkcs.2011.32.6.2032

59. Breyholz H-J, Schäfers M, Wagner S, Höltke C, Faust A, Rabeneck H, et al. C-5-Disubstituted Barbiturates as Potential Molecular Probes for Noninvasive Matrix Metalloproteinase Imaging. J Med Chem. 2005;48: 3400–3409. doi:10.1021/jm049145x

60. Wang D, Pei P, Shea FF, Bissonnette C, Nieto K, Din C, et al. Fenretinide combines perturbation of signaling kinases, cell-extracellular matrix interactions and matrix metalloproteinase activation to inhibit invasion in oral squamous cell carcinoma cells. Carcinogenesis. 2022;43: 851–864. doi:10.1093/carcin/bgac070

61. Marques SM, Tuccinardi T, Nuti E, Santamaria S, André V, Rossello A, et al. Novel 1-Hydroxypiperazine-2,6-diones as New Leads in the Inhibition of Metalloproteinases. J Med Chem. 2011;54: 8289–8298. doi:10.1021/jm200593b

62. Levin JI, Chen J, Du M, Hogan M, Kincaid S, Nelson FC, et al. The discovery of anthranilic acid-Based MMP inhibitors. Part 2: SAR of the 5-position and P11 groups. Bioorg Med Chem Lett. 2001;11: 2189–2192. doi:10.1016/S0960-894X(01)00419-X

63. Marques SM, Nuti E, Rossello A, Supuran CT, Tuccinardi T, Martinelli A, et al. Dual inhibitors of matrix metalloproteinases and carbonic anhydrases: iminodiacetyl-based hydroxamate-benzenesulfonamide conjugates. J Med Chem. 2008;51: 7968–7979. doi:10.1021/jm800964f

64. Zhang H, Wang X, Mao J, Huang Y, Xu W, Duan Y, et al. Synthesis and biological evaluation of novel benzofuroxan-based pyrrolidine hydroxamates as matrix metalloproteinase inhibitors with nitric oxide releasing activity. Bioorg Med Chem. 2018;26: 4363–4374. doi:10.1016/j.bmc.2018.06.023

65. Nuti E, Casalini F, Avramova SI, Santamaria S, Fabbi M, Ferrini S, et al. Potent arylsulfonamide inhibitors of tumor necrosis factor-alpha converting enzyme able to reduce activated leukocyte cell adhesion molecule shedding in cancer cell models. J Med Chem. 2010;53: 2622–2635. doi:10.1021/jm901868z

66. Sang QX, Jia MC, Schwartz MA, Jaye MC, Kleinman HK, Ghaffari MA, et al. New thiol and sulfodiimine metalloproteinase inhibitors and their effect on human microvascular endothelial cell growth. Biochem Biophys Res Commun. 2000;274: 780–786. doi:10.1006/bbrc.2000.3212

67. Nuti E, Panelli L, Casalini F, Avramova SI, Orlandini E, Santamaria S, et al. Design, Synthesis, Biological Evaluation, and NMR Studies of a New Series of Arylsulfones As Selective and Potent Matrix Metalloproteinase-12 Inhibitors. J Med Chem. 2009;52: 6347–6361. doi:10.1021/jm900335a

68. Cheng M, De B, Pikul S, Almstead NG, Natchus MG, Anastasio MV, et al. Design and Synthesis of Piperazine-Based Matrix Metalloproteinase Inhibitors. J Med Chem. 2000;43: 369–380. doi:10.1021/jm990366q

69. Levin JI, DiJoseph JF, Killar LM, Sung A, Walter T, Sharr MA, et al. The synthesis and biological activity of a novel series of diazepine MMP inhibitors. Bioorg Med Chem Lett. 1998;8: 2657–2662. doi:10.1016/S0960-894X(98)00473-9

70. Zhang Y-M, Fan X, Xiang B, Chakravarty D, Scannevin R, Burke S, et al. Synthesis and SAR of α-sulfonylcarboxylic acids as potent matrix metalloproteinase inhibitors. Bioorg Med Chem Lett. 2006;16: 3096–3100. doi:10.1016/j.bmcl.2006.03.065

71. Wilson LJ, Wang B, Yang S-M, Scannevin RH, Burke SL, Karnachi P, et al. Discovery of novel Cobactin-T based matrix metalloproteinase inhibitors via a ring closing metathesis strategy. Bioorg Med Chem Lett. 2011;21: 6485–6490. doi:10.1016/j.bmcl.2011.08.068

72. Wang L, Li X, Zhang S, Lu W, Liao S, Liu X, et al. Natural products as a gold mine for selective matrix metalloproteinases inhibitors. Bioorg Med Chem. 2012;20: 4164–4171. doi:10.1016/j.bmc.2012.04.063

73. María Zapico J, Puckowska A, Filipiak K, Coderch C, Pascual-Teresa B de, Ramos A. Design and synthesis of potent hydroxamate inhibitors with increased selectivity within the gelatinase family. Org Biomol Chem. 2015;13: 142–156. doi:10.1039/C4OB01516A

74. Adhikari N, Halder AK, Mallick S, Saha A, Saha KD, Jha T. Robust design of some selective matrix metalloproteinase-2 inhibitors over matrix metalloproteinase-9 through in silico/fragment-based lead identification and *de novo* lead modification: Syntheses and biological assays. Bioorg Med Chem. 2016;24: 4291–4309. doi:10.1016/j.bmc.2016.07.023

75. Hanessian S, Bouzbouz S, Boudon A, Tucker GC, Peyroulan D. Picking the S1, S1’ and S2’ pockets of matrix metalloproteinases. A niche for potent acyclic sulfonamide inhibitors. Bioorg Med Chem Lett. 1999;9: 1691–1696. doi:10.1016/s0960-894x(99)00259-0

76. Miyanaga S, Sakurai H, Saiki I, Onaka H, Igarashi Y. Synthesis and evaluation of myxochelin analogues as antimetastatic agents. Bioorg Med Chem. 2009;17: 2724–2732. doi:10.1016/j.bmc.2009.02.040

77. Hariono M, Nuwarda RF, Yusuf M, Rollando R, Jenie RI, Al-Najjar B, et al. Arylamide as Potential Selective Inhibitor for Matrix Metalloproteinase 9 (MMP9): Design, Synthesis, Biological Evaluation, and Molecular Modeling. J Chem Inf Model. 2020;60: 349–359. doi:10.1021/acs.jcim.9b00630

78. Zhang Y, Tounge BA, Wang A, Hawkins M, Leonard KA, Barbay JK, et al. Pyridyl-thiazolyl inhibitors of pro-matrix metalloproteinase activation. US20120129843A1, 2012. Available: https://patents.google.com/patent/US20120129843A1/en

79. Shi Z-H, Li N-G, Shi Q-P, Tang H, Tang Y-P, Li W, et al. Synthesis and structure–activity relationship analysis of caffeic acid amides as selective matrix metalloproteinase inhibitors. Bioorg Med Chem Lett. 2013;23: 1206–1211. doi:10.1016/j.bmcl.2013.01.027

80. Wang A, Zhang Y, Leonard KA, Hawkins M, Tounge BA, Maharoof USM, et al. Tricyclic inhibitors of pro-matrix metalloproteinase activation. US20120129811A1, 2012. Available: https://patents.google.com/patent/US20120129811A1/en

81. Leonard KA, Zhang Y, Tounge BA, Wang A, Hawkins M, Jackson PF, et al. Fused heteroaryl inhibitors of pro-matrix metalloproteinase activation. US20120129872A1, 2012. Available: https://patents.google.com/patent/US20120129872A1/en

82. Becker DP, Barta TE, Bedell LJ, Boehm TL, Bond BR, Carroll J, et al. Orally Active MMP-1 Sparing α-Tetrahydropyranyl and α-Piperidinyl Sulfone Matrix Metalloproteinase (MMP) Inhibitors with Efficacy in Cancer, Arthritis, and Cardiovascular Disease. J Med Chem. 2010;53: 6653–6680. doi:10.1021/jm100669j

83. Pikul S, Dunham KM, Almstead NG, De B, Natchus MG, Taiwo YO, et al. Heterocycle-based MMP Inhibitors with P2′ Substituents. Bioorg Med Chem Lett. 2001;11: 1009–1013. doi:10.1016/S0960-894X(01)00137-8

84. Ananthan S. Nonpeptide inhibitors of matrix metalloproteinases. EP1735274B1, 2009. Available: https://patents.google.com/patent/EP1735274B1/en

85. Baxter AD, Bhogal R, Bird J, Keily JF, Manallack DT, Montana JG, et al. Arylsulphonyl hydroxamic acids: potent and selective matrix metalloproteinase inhibitors. Bioorg Med Chem Lett. 2001;11: 1465–1468. doi:10.1016/S0960-894X(01)00259-1

86. Yang L, Ma X, Guo K, Li J, Zhang C, Wu L. Dual-functional antitumor conjugates improving the anti-metastasis effect of combretastatin A4 by targeting tubulin polymerization and matrix metalloproteinases. Eur J Med Chem. 2022;238: 114439. doi:10.1016/j.ejmech.2022.114439

87. Agamennone M, Belov DS, Laghezza A, Ivanov VN, Novoselov AM, Andreev IA, et al. Fragment-Based Discovery of 5-Arylisatin-Based Inhibitors of Matrix Metalloproteinases 2 and 13. ChemMedChem. 2016;11: 1892–1898. doi:10.1002/cmdc.201600266

88. Freskos JN, Asmelash B, Gaston KR, Karwa A, Marzan TA, Nickols MA, et al. Design and synthesis of MMP inhibitors with appended fluorescent tags for imaging and visualization of matrix metalloproteinase enzymes. Bioorg Med Chem Lett. 2013;23: 5566–5570. doi:10.1016/j.bmcl.2013.08.050

89. Ronsisvalle S, Panarello F, Longhitano G, Siciliano EA, Montenegro L, Panico A. Natural Flavones and Flavonols: Relationships among Antioxidant Activity, Glycation, and Metalloproteinase Inhibition. Cosmetics. 2020;7: 71. doi:10.3390/cosmetics7030071

90. Zhu Y, Huang R-Z, Wang C-G, Ouyang X-L, Jing X-T, Liang D, et al. New inhibitors of matrix metalloproteinases 9 (MMP-9): Lignans from Selaginella moellendorffii. Fitoterapia. 2018;130: 281–289. doi:10.1016/j.fitote.2018.09.008

91. Aranapakam V, Davis JM, Grosu GT, Baker, Ellingboe J, Zask A, et al. Synthesis and Structure−Activity Relationship of N-Substituted 4-Arylsulfonylpiperidine-4-hydroxamic Acids as Novel, Orally Active Matrix Metalloproteinase Inhibitors for the Treatment of Osteoarthritis. J Med Chem. 2003;46: 2376–2396. doi:10.1021/jm0205550
