# Supplementary material for: Integrative computational, synthetic, experimental evaluation of targeted inhibitors against matrix metalloproteinase-9: Toward precision modulation of proteolytic activity
Source: PLoS One. 2026 Feb 17;21(2):e0337544. doi: 10.1371/journal.pone.0337544 (PMC12912705; doi:10.1371/journal.pone.0337544)
Supplement: Attachment — Submitted filename: PONE-D-25-47277_comments.pdf [file pone.0337544.s007.pdf]

The manuscript PONE-D-25-47277 presents a new approach to developing a pharmacophore model for inhibitors of matrix metalloproteinase-9 (MMP-9). The development of inhibitors of MMP-9 would benefit therapeutic approaches to rheumatoid arthritis, atherosclerosis, and cancer metastasis. The authors have identified several potential lead compounds through computational docking and assayed their inhibitor activity.

The research and approach to this problem are significant advancements in knowledge. The manuscript is recommended for publication after major revisions, particularly to the narrative and figures for the Results and Discussion section.

A minor correction is needed on line 441 for the inhibitor concentration, should be 50 μM.

Figure 4 should be remade to match the proportions of figure 3. The images appear to be stretched and squashed by inappropriate resizing.

The descriptions on line 558 about "lung cancer cell lines" and on line 565 seem inaccurate regarding molecular modeling for the "anticancer effect of MMP-9 inhibitors against lung cancer cell lines." The phrasing seems unnecessary and is not mentioned elsewhere and the no assays using lung cancer cell lines were included in this study. Only the colorimetric inhibitor screening assay was reported in this manuscript.

The remainder of the Results and Discussion starting on line 588 needs to be carefully rewritten by the authors. Several mistakes were found by this reviewer and there are likely more problems that should be addressed in a major revision of this section.

The paragraph starting on line 588 seems incomplete and does not identify important amino acids that are referenced in the following paragraph. On line 594, reference 65 seems incorrect as note in more detail below since the reference study focused on antibodies and allosteric inhibition of MMP-9. The description of a "another" study starting on line 596 does not include a reference to that study. On line 604, "docking study" starts an incomplete sentence. The reference 65 describes the antigenic epitope residues of Arg162, Glu111, Asp113 and Ile198, but made no mention of any residues near the substrate binding site. Their assays suggest that the antibodies are non-competitive and allosteric inhibitors. On line 609, the experimental crystal structure (1GKC) is incorrectly presented as a docking study.

The results of the molecular docking of synthesized compounds and commercially available compounds is not presented in a way that can be appreciated by the reader (note there are no line numbers in this portion of the manuscript). The description of compound 8 docking into MMP-9 (1GKC) highlights hydrogen bonding interactions with Arg424, Leu188 and Pro421. In figure 6B, compound 8 is not close enough to Arg424 to form a hydrogen bond and neither Leu188 nor Pro421 are shown in the figure. If these interactions are only apparent after Glide of induced fit modeling, a figure of showing the modeled MMP-9 structure should be included. Similarly, for compound 27, there are hydrogen bonds (inconsistently called H-bonds) to Pro421, Leu188, Tyr393 and Tyr420 described in the docking results. In figure 7B, neither Pro421 nor

Leu188 are visible and both Tyr393 and Tyr420 appear too far away. Again a figure of the docking model should be shown to highlight these interactions which may not be apparent in the superposition with the crystal structure. Lastly, the docking of the commercially available compounds NSC3276 and NSC123019 both appear to have steric clashes with the backbone of Met 422 in figure 8. Again, this may be an artifact of the superposition and should be addressed by include figures of the docked protein structures.

The supplementary tables S4 and S5 cite references that are not specified in the manuscript bibliography. The bibliography for the supplementary material appears to have been omitted from the submission.

Please note that the figure legends appearing within the narrative appears to be an formatting mistake and detracts from the review process.
