# Supplementary material for: Integrative computational, synthetic, experimental evaluation of targeted inhibitors against matrix metalloproteinase-9: Toward precision modulation of proteolytic activity
Source: PLoS One. 2026 Feb 17;21(2):e0337544. doi: 10.1371/journal.pone.0337544 (PMC12912705; doi:10.1371/journal.pone.0337544)
Supplement: Attachment — Submitted filename: Response to reviwer_PONE-D-25-47277_October_29th.docx [file pone.0337544.s009.docx]

**Confirmation Letter**

Dear Esteemed Editor,

Thank you for your letter and constructive comments concerning our manuscript entitled " **Integrative computational, synthetic, experimental evaluation of targeted inhibitors against Matrix Metalloproteinase-9: Toward precision modulation of proteolytic activity**” Reference#: PONE-D-25-47277. We have carefully addressed your comments and made major corrections, which we hope meet with your approval. Please consider our point-by-point responses to the reviewers in the following texts. All the changes have been considered and highlighted in yellow color in the manuscript.

**Reviewer #1:**

This study identifies potential inhibitors of matrix metalloproteinase-9 (MMP-9), a zinc-dependent enzyme linked to various diseases. Several compounds demonstrated over 60% inhibition, with compounds 2 and 20 showing IC_50_ values of 28.59 μM and 30.82 μM. Computational docking aligned with experimental validation, confirming specificity, potency, and key biological interactions.

However, there are several shortcomings in this work that need to be addressed. The authors should carefully revise the manuscript for clarity, correct grammatical and typographical errors, ensure consistent formatting, and eliminate all proofreading issues before it can be considered further.

- We sincerely thank the esteemed reviewer for this recognition and valuable recommendation.

**1- Reviewer Comment: Many chemical structures are not geometrically accurate and need to be redrawn.**

- **Author Response:** We appreciate the reviewer for highlighting this significant point. All chemical structures have been updated and redrawn to guarantee precise geometry. The corrected chemical structures were included and highlighted in the main manuscript.

**2- Reviewer Comment: The amide bonds in all chemical structures should ideally be shown in the anti- conformation, which is more stable than the syn- conformation.**

- **Author Response:** Thank you for your valuable comment. All chemical structures in the manuscript have been modified to depict amide bonds in a more stable, anti-conformation form. The revised structures were added to the manuscript.

**3- Reviewer Comment: Abbreviations need to be consistent throughout the manuscript.**

- **Author Response:** We thank the reviewer for noting this point. All abbreviations are now defined at their first appearance and consistently listed throughout the manuscript.

**4- Reviewer Comment:**  **Use either hours, hr, or h for reaction time, not a mix.**

- **Author Response:** The unit of the reaction time has been standardized throughout the manuscript and is now consistently presented as hr.

**5- Reviewer Comment: Temperature should be written as “X** **ºC” (with a space), not “X****ºC.”**

- **Author Response:** Thank you for your helpful comment. A space has now been inserted between all the temperature symbols in the manuscript and the numbers.

**6- Reviewer Comment: “Diethyl ether” should be written without a hyphen.**

- **Author Response:** The chemical nomenclature of dimethyl ether has been adjusted throughout the manuscript from diethyl-ether to diethyl ether.

**7- Reviewer Comment:** **“Rf” should be written correctly in the experimental details.**

- **Author Response:** We thank the reviewer for this comment; the experimental details were revised and corrected to improve consistency throughout the manuscript.

**8- Reviewer Comment: The (M+Na)^+^ values in compound 8 need correction for significant figures.**

- **Author Response:** We appreciate the reviewer's observation. The (M+Na)^+^ value has been corrected to ensure consistent significant figures, and this correction has been applied to all other HRMS results for uniformity.

**9- Reviewer Comment: In the general methods, the stoichiometry of amines is written as 1:1 eq. This should be clarified and corrected.**

- **Author Response:** Regarding the synthetic methods, they have been performed using an equimolar ratio (1:1) of the nucleus and the corresponding amine derivatives. The practical reason behind this, is the stoichiometry of the reaction, in which the reaction involves simple one-to-one coupling; therefore, the use of equimolar amounts is considered correct. Additionally, it reduces side reactions associated with excess amine, thereby reducing impurity formation and the need for complex purification.
- **Here are the corrected and clarified sentences added to the manuscript**: “Then, a corresponding amine derivative (4i-xi) was dissolved in an anhydrous CHCl_3_ (15 mL) and added portion-wise to the reaction in an equimolar (1:1) ratio relative to the nucleus.” “Then, a corresponding amine derivative (31i-x) was dissolved in an anhydrous CHCl_3_ (15 mL) and added to the reaction mixture portion-wise in an equimolar (1:1) ratio relative to the nucleus, and the solution was refluxed for 24 hr.”

**10- Reviewer Comment: The use of 6 equivalents of pyridine is unusually large compared to the amine. The authors should justify this choice, as typically a 1:1 ratio of base/amine is sufficient.**

- **Author Response:** We appreciate the reviewer's important comment. In our synthesis, pyridine was used in sixfold excess relative to the amine for several reasons. Firstly, it acts as a base to neutralize acidic by-products generated during the reaction. Secondly, since amine derivatives are bulky groups, the excess pyridine helps catalyze the process by enhancing nucleophilic attack and overcoming the steric hindrance caused by the bulky amines, thereby ensuring complete conversion and a higher product yield.

**11- Reviewer Comment: In the synthesis section, the statement “the product was collected after solvent evaporation, followed by recrystallization with CHCl3/n-hexane (19:20)” seems questionable. The solvent ratio should be verified.**

- **Author Response:** We appreciate the reviewer's comment for pointing this out. The notation “19:20” actually cites previous studies on the synthetic procedure, not a solvent ratio. We apologize for this error. When updating references in Zotero, this one was not revised, leading to the incorrect format. To prevent confusion, it has now been corrected in the revised manuscript as a proper reference, appearing as “[21,22]” following to the journal's guidelines.

**12- Reviewer Comment: In Scheme 2, the condition “2) 80 °C, 2 hours” is unclear. Please clarify what it refers to.**

- **Author Response:** The formation of acyl (indole-2-carbonyl chloride) using oxalyl chloride, anhydrous chloroform, and a few drops of DMF was performed under a reflux system at 80 °C for 2 hours. The scheme legend has been updated in the revised and highlighted in the manuscript to clarify this point.

**13- Reviewer Comment: In Scheme 2, the condition “CHCl_3_, pyridine, 80 °C, 24 hr” is problematic since chloroform boils at 61 ºC. The authors should clarify whether this refers to reflux conditions. The same applies to Scheme 1.**

- **Author Response:** We sincerely appreciate the reviewer’s valuable comments aimed at improving the quality of our manuscript. The synthetic reactions involving CHCl₃ were conducted under a reflux setup to prevent solvent loss and local overheating. This configuration maintains the reaction at the solvent’s boiling point, ensuring efficient heat transfer and complete conversion of the starting materials. Refluxing also promotes the forward direction of the reaction, particularly for sterically hindered amine derivatives, thereby improving yields.

As noted, the reactions employed a co-solvent system consisting of chloroform with a few drops of DMF. The small amount of DMF slightly increases the boiling point of the mixture, and therefore the reflux temperature was maintained at approximately 80 °C to ensure continuous boiling and homogeneous mixing. This temperature reflects the actual reflux condition of the CHCl₃/DMF system under our experimental setup rather than the boiling point of pure chloroform (≈61 °C). The scheme legends have been revised to specify the reflux conditions used in this study clearly.

**14- Reviewer Comment: The target compounds are incorrectly referred to as “benzoyl chloride” and “indole-2-carbonyl chloride.” These are amides, not acyl chlorides. The nomenclature must be corrected.**

- **Author Response:** The nomenclature for the two synthesized scaffolds has been revised to identify the target compounds, benzamide and 1H-indole-2-carboxamide, correctly, and this revision has been implemented throughout the manuscript.

**15- Reviewer Comment:**  **In Table 6, % inhibition and IC_50_ values are reported with inconsistent significant figures. These should be standardized.**

- **Author Response:** We are grateful to the reviewer for this observation. The %inhibition and IC_50_ values in Table 6 were standardized and rounded to 2 decimal places to reflect the precision of MMP-9 inhibition accurately.

**Reviewer #2:**

The manuscript PONE-D-25-47277 presents a new approach to developing a pharmacophore model for inhibitors of matrix metalloproteinase-9 (MMP-9). The development of inhibitors of MMP-9 would benefit therapeutic approaches to rheumatoid arthritis, atherosclerosis, and cancer metastasis. The authors have identified several potential lead compounds through computational docking and assayed their inhibitor activity.

The research and approach to this problem are significant advancements in knowledge. The manuscript is recommended for publication after major revisions, particularly to the narrative and figures for the Results and Discussion section.

- We sincerely thank the esteemed reviewer for this recognition and valuable recommendation.

**1-Reviwer Comment:** **A minor correction is needed on line 441 for the inhibitor concentration, should be 50** μ**M.**

- **Author Response:** We thank the reviewer for pointing this out. The concentration was corrected with the proper unit.

**2- Reviewer Comment:** **Figure 4 should be remade to match the proportions of figure 3. The images appear to be stretched and squashed by inappropriate resizing.**

- **Author Response:** We thank the esteemed reviewer for this valuable comment. We manipulated the image in Horizontal and Longitudinal positions for clarification purposes. The respectful reviewer can assign the appropriate image.

**Figure 4.** MMP-9 pharmacophore model with purchased partial-match hits: (A) NSC3276; (**B**) **NSC123019**. Aro stands for the aromatic ring, Hyd: hydrophobic, Acc: acceptor, Don: donor group. Picture made by MOE.

**Figure 4.** MMP-9 pharmacophore model with purchased partial-match hits: (A) NSC3276; (**B**) **NSC123019**. Aro stands for the aromatic ring, Hyd: hydrophobic, Acc: acceptor, Don: donor group. Picture made by MOE.

**3-** **Reviewer Comment:** **The descriptions on line 558 about “lung cancer cell lines” and on line 565 seem inaccurate regarding molecular modeling for the “anticancer effect of MMP-9 inhibitors against lung cancer cell lines.” The phrasing seems unnecessary and is not mentioned elsewhere and the no assays using lung cancer cell lines were included in this study. Only the colorimetric inhibitor screening assay was reported in this manuscript.**

- **Author Response:** We thank the reviewer for this important observation. We apologize for the inaccurate description of the experimental evaluation. The experimental evaluation was conducted using a colorimetric MMP-9 inhibitor activity screening assay kit, not with a cancer cell line. This point has been corrected and clearly specified in the manuscript.

**4-** **Reviewer Comment:** The remainder of the Results and Discussion starting on line 588 needs to be carefully rewritten by the authors. Several mistakes were found by this reviewer and there are likely more problems that should be addressed in a major revision of this section. The paragraph starting on line 588 seems incomplete and does not identify important amino acids that are referenced in the following paragraph. On line 594, reference 65 seems incorrect as note in more detail below since the reference study focused on antibodies and allosteric inhibition of MMP-9. The description of a “another” study starting on line 596 does not include a reference to that study. On line 604, “docking study” starts an incomplete sentence. The reference 65 describes the antigenic epitope residues of Arg162, Glu111, Asp113 and Ile198, but made no mention of any residues near the substrate binding site. Their assays suggest that the antibodies are non-competitive and allosteric inhibitors. On line 609, the experimental crystal structure (1GKC) is incorrectly presented as a docking study.

**Author Response:** We thank the reviewer for their careful observation. The results and discussion sections have been reorganized for better clarity and logical flow. Currently, the results emphasize the key amino acid residues in the MMP-9 binding pocket (for both wild and mutant forms) before discussing their importance in relation to other cited computational studies. We also apologize for including reference 65, which describes MMP-9 inhibitor binding in the allosteric site—an area unrelated to our focus on active site inhibition—so it has been revised and removed from the discussion. Additionally, after rearranging the paragraph, the correct reference for the study mentioned is now properly cited. Regarding the study in line 609, we agree that the 1GKC structure is an experimentally determined crystal structure, not a docking study. Thus, the paragraph was updated to accurately state that we use the 1GKC crystal structure to validate our docking protocol rather than our docking results.

**5-** **Reviewer Comment:** The results of the molecular docking of synthesized compounds and commercially available compounds is not presented in a way that can be appreciated by the reader (note there are no line numbers in this portion of the manuscript). The description of compound 8 docking into MMP-9 (1GKC) highlights hydrogen bonding interactions with Arg424, Leu188 and Pro421. In figure 6B, compound 8 is not close enough to Arg424 to form a hydrogen bond and neither Leu188 nor Pro421 are shown in the figure. If these interactions are only apparent after Glide of induced fit modeling, a figure of showing the modeled MMP-9 structure should be included. Similarly, for compound 27, there are hydrogen bonds (inconsistently called H-bonds) to Pro421, Leu188, Tyr393 and Tyr420 described in the docking results. In figure 7B, neither Pro421 nor Leu188 are visible and both Tyr393 and Tyr420 appear too far away. Again, a figure of the docking model should be shown to highlight these interactions which may not be apparent in the superposition with the crystal structure. Lastly, the docking of the commercially available compounds NSC3276 and NSC123019 both appear to have steric clashes with the backbone of Met 422 in figure 8. Again, this may be an artifact of the superposition and should be addressed by include figures of the docked protein structures.

**Author Response:** We thank the respectful reviewer for this valuable comment and for carefully examining the docking figures. **We have edited the 3D ligand/receptor interactions poses in Figures (5, 6, 7, and 8) to address the comments**. The docking results were generated using the Glide and Induced Fit Docking (IFD) protocols implemented in the Schrödinger Suite. The 2D ligand interaction is derived using the script “Ligand interaction in MAESTRO in Schrodinger enterprise” for the top-docked pose, while the 3D complex is visualized in PYMOL in Schrodinger enterprise. We acknowledge that minor differences in apparent residue proximity can arise when switching between the 2D ligand-interaction maps and 3D structural representations. These differences are due to differences in projection, scaling, and orientation, rather than inaccuracies in the docking output. Specifically, the IFD protocol allows for local flexibility in both the ligand and nearby side chains, which can slightly shift residue positions relative to the static crystal structure. This explains why residues such as Arg424, Leu188, and Pro421 (for compound 8), or Pro421, Leu188, Tyr393, and Tyr420 (for compound 27) may appear differently in the static overlay figures but are within hydrogen-bonding distance in the optimized IFD complex. Additionally, steric clashes are evaluated based on the highest docked pose that fits the binding site. We did not include all amino acids to simplify and clarify. Ultimately, the proximity or distance of amino acids cannot be generalized since Schrodinger algorithms are highly accurate.

**Figure 5.** The binding domains of (**A**) WT MMP-9 (PDB ID: 1GKC) and (**B**) MUT MMP-9 (PDB ID: 2OW1) accommodate the verified series. Carbon (C) atoms are depicted in grey color, N blue color, and O red color. H bonds and valence were removed for clarification. Zinc metal is presented as a sphere (metallic color). Picture visualized by PYMOL[61].

**Figure 6.** The binding domains of wild MMP-9 (PDB ID: 1GKC). (**A**) Orientation of IF docked poses of benzoyl chloride compounds and (**B**) overlaying of the IF docked pose of **compound 8** with the co-crystallized ligand (NFH) (yellow color). Carbon (C) atoms are depicted in grey colors, N in blue color, and O in red color. **Compound 5** depicted in cyan color, **compound 6** magenta color, **compound 8** in orange color, **compound 9** in green color, **compound 10** in blue color, **compound 11** in purple-blue color, **compound 14** in pink color, **compound 7** in deep blue color, **compound 13** in deep cyan color, **compound 12** in sand color, and **compound 15** in red color. H bonds and valence were removed for clarification. Zinc metal is presented as a sphere (metallic color). Picture visualized by PYMOL [61].

**Figure 7.** The binding domains of wild MMP-9 (PDB ID: 1GKC). (**A**) Orientation of induced-fit docked poses of Indole-2-carbonyl chloride compounds and (**B**) overlaying of the induced-fit docked pose of **compound 27** with co-crystalized ligand (NFH) (yellow color). Carbon (C) atoms are depicted in grey colors, N in blue color, and O in red color. **Compound 29** is depicted in marine color, **compound 20** in green color, **compound 21** in red color, **compound 22** in blue color, **compound 23** in magenta color, **compound 24** in cyan color, **compound 25** in orange color, **compound 26** in purple color, **compound 27** in pink color, and **compound 28** in deep teal color. H bonds and valence were removed for clarification. Zinc metal is presented as a sphere (metallic color). Picture visualized by PYMOL [61].

**Figure 8.** The orientation of induced-fit docked poses of **NSC3276** and **NSC123019** with co-crystalized ligand (NFH) (yellow color) in 1GKC. Carbon (C) atoms are depicted in grey colors, N in blue color, and O in red color. **NSC3276** is depicted in magenta color and **NSC123019** in cyan color. Zinc metal is presented as a sphere (metallic color). H bonds and valence were removed for clarification. Picture visualized by PYMOL [61].

**6-** **Reviewer Comment:** **The supplementary tables S4 and S5 cite references that are not specified in the manuscript bibliography. The bibliography for the supplementary material appears to have been omitted from the submission.**

**Author Response:** Tables S4 and S5 present the docking results for approximately 150 previously reported MMP-9 inhibitors. To keep the main manuscript concise, these tables have been moved to the supplementary materials and clearly cited in the main manuscript. Additionally, the references bibliography is now included in the supplementary file to ensure proper documentation.

**7- Reviewer Comment:** **Please note that the figure legends appearing within the narrative appears to be a formatting mistake and detracts from the review process.**

**Author Response:** As per PLOS ONE manuscript guidelines, figure legends should be placed immediately after the paragraph where they are first cited. We have formatted the manuscript accordingly to adhere to these guidelines. Additionally, we double-checked all figure legends to ensure they are correctly positioned and do not disrupt the narrative flow. We attempted to increase the spacing between paragraph and figure legends to minimize interruptions and facilitate a smoother review process.
